# Genetic Adaptation to Brackish Water and Spawning Season in European Cisco

**DOI:** 10.1111/mec.70094

**Published:** 2025-09-03

**Authors:** Qiaoling Deng, Jake Goodall, Mikaela Bergenius Nord, Ignas Bunikis, Arianna Cocco, Bo Delling, Elisabet Einarsdottir, Julia Heintz, Henrik Lantz, Kerstin Lindblad‐Toh, Mai‐Britt Mosbech, Remi‐Andre Olsen, Stefan Palm, Mats E. Pettersson, Martin Pippel, Lucile Soler, Anti Vasemägi, Olga Vinnere Pettersson, Leif Andersson

**Affiliations:** ^1^ Department of Medical Biochemistry and Microbiology Uppsala University Uppsala Sweden; ^2^ SciLifeLab Uppsala University Uppsala Sweden; ^3^ The Key Laboratory of Aquatic Biodiversity and Conservation of Chinese Academy of Sciences Institute of Hydrobiology, Chinese Academy of Sciences Wuhan Hubei China; ^4^ Department of Aquatic Resources, Institute of Marine Research Swedish University of Agricultural Sciences Lysekil Sweden; ^5^ SciLifeLab Genomics Platform, NGI, Department of Immunology, Genetics and Pathology Uppsala University Uppsala Sweden; ^6^ Department of Zoology Swedish Museum of Natural History Stockholm Sweden; ^7^ SciLifeLab, Department of Gene Technology KTH‐Royal Institute of Technology Solna Sweden; ^8^ SciLifeLab, National Bioinformatics Infrastructure Sweden (NBIS), Department of Medical Biochemistry and Microbiology Uppsala University Uppsala Sweden; ^9^ Broad Institute of MIT and Harvard Cambridge Massachusetts USA; ^10^ SciLifeLab, Department of Biochemistry and Biophysics Stockholm University Solna Sweden; ^11^ Department of Aquatic Resources, Institute of Freshwater Research Swedish University of Agricultural Sciences Drottningholm Sweden; ^12^ SciLifeLab, National Bioinformatics Infrastructure Sweden (NBIS), Department of Cell and Molecular Biology Uppsala University Uppsala Sweden; ^13^ Department of Aquaculture Estonian University of Life Sciences, Institute of Veterinary Medicine and Animal Sciences Tartu Estonia; ^14^ Department of Veterinary Integrative Biosciences, College of Veterinary Medicine and Biomedical Sciences Texas A&M University College Station Texas USA

**Keywords:** European cisco, genetic adaptation, photoperiodism, population structure, whole genome sequencing

## Abstract

How species adapt to diverse environmental conditions is essential for understanding evolution and the maintenance of biodiversity. The European cisco ( *Coregonus albula* ) is a salmonid that occurs in both fresh and brackish water, and this together with the presence of sympatric spring‐ and autumn‐spawning lacustrine populations provides an opportunity for studying the genetics of adaptation in relation to salinity and timing of reproduction. Here, we present a high‐quality reference genome of the European cisco based on PacBio HiFi long read sequencing and HiC‐directed scaffolding. We generated low‐coverage whole‐genome sequencing data from 336 individuals across 12 population samples to explore population structure and genetics of ecological adaptation. We found a major subdivision between two groups of populations most likely reflecting colonisation from different glacial refugia. Within the two major groups, we detected further genetic differentiation between spring‐ and autumn‐spawning populations and between populations from freshwater lakes, rivers and brackish water (Bothnian Bay). A genome‐wide screen for genetic differentiation among populations identified a set of outlier SNPs strongly correlated with spawning timing and salinity. Several of the genes associated with spawning time, including *BHLHE40*, *TIMELESS* and *CPT1A*, have previously been shown to have a role in circadian rhythm biology. As many as 17 loci were associated with genetic differentiation between populations reproducing in fresh and brackish water. This study provides insights into the genomic basis of ecological adaptation in European cisco with implications for sustainable fishery management.

## 1 Introduction

How species adapt to diverse environmental conditions is essential for understanding evolution and the maintenance of biodiversity. A widely distributed species that occurs across environmental gradients must tolerate diverse conditions. This can be accomplished by individual‐level phenotypic plasticity (Enbody et al. 2021; Salisbury and Ruzzante 2022) and may lead to genetic adaptation to local environmental conditions (Barrett and Schluter 2008; Savolainen et al. 2013; Berg et al. 2015; Han et al. 2020). The relative importance of adaptive divergence vs. phenotypic plasticity is still not well understood (Ghalambor et al. 2007; Fox et al. 2019), but whole genome comparison of populations adapted to different environments is a promising strategy to reveal the importance of genetic adaptation.

Ecotypic differentiation, involving changes in morphology, feeding and spawning behaviour, is common among salmonids, making them excellent systems for studying the processes of local adaptation and speciation (Garcia de Leaniz et al. 2007; Fraser et al. 2011; Salisbury and Ruzzante 2022). Additionally, characterising the population structure and the genomic basis of ecotypic differentiation in these species has cultural and socio‐economic implications as they are an important food source in many parts of the world and often support significant commercial and recreational fishing activities. Anthropogenic pressures and climate change constitute threats to the sustainable maintenance of populations (Elliott and Bell 2011; Hansen et al. 2009; Wenger et al. 2011; Kangur et al. 2020).

The European cisco ( *Coregonus albula* ) inhabits many freshwater lakes in Northern Europe, with a preference for deep and oligotrophic environments (Vuorinen and Lankinen 1978; Sendek 2021; Karjalainen et al. 2022). It also occurs in the brackish Baltic Sea basin, particularly in the northern Bothnian Bay where it constitutes the basis for a substantial fishery (Bergenius et al. 2013; Lehtonen et al. 2023) and in the eastern part of the Gulf of Finland (Sendek 2012; Lehtonen et al. 2023). In these regions, with a salinity of only about 3‰, both anadromous and coastal spawning ciscoes occur, the latter of which are adapted to reproducing in the brackish sea water. This ecological adaptation may be relatively recent, since the brackish Baltic Sea has only existed for 8000 years following the last glaciation (Andrén et al. 2011).

Although autumn spawning is the norm in European cisco, some populations reproduce during winter or spring (e.g., Huitfeldt‐Kaas 1927). Sympatric populations with different spawning periods (spring vs. autumn) sometimes occur within the same lake, as documented in Germany, Finland and Sweden (Svärdson 1979; Vuorinen et al. 1981; Schulz et al. 2006), most likely reflecting genetic differences in photoperiodic regulation of reproduction as documented in other vertebrates (Chen et al. 2020). For instance, spring‐ and autumn‐spawning Atlantic herring show strong genetic differentiation at a handful of loci (Han et al. 2020).

The occurrence of European ciscoes reproducing in either freshwater or brackish environments, along with the species' flexibility in spawning period, including the existence of sympatric spring‐ and autumn‐spawning populations, presents an ideal situation for population genetic studies of adaptive evolution. Previous genetic comparisons based on mitochondrial DNA and microsatellites have demonstrated postglacial independent origins of sympatric spring‐ and autumn‐spawning populations (Schulz et al. 2006; Delling et al. 2014; Mehner et al. 2021). However, the genomic architecture underlying ecological adaptation in European cisco is largely unknown, although a previous study using reduced representation sequencing (RAD‐seq) found genetic differentiation between freshwater Kalix River and other sampling locations in the Bothnian Bay area (López et al. 2022). This study detected 41 SNPs putatively associated with genomic regions under divergent selection. However, the sparseness of markers assessed in this study, together with the lack of a reference genome, has precluded further characterisation of these putative signals of selection.

**FIGURE 1:**
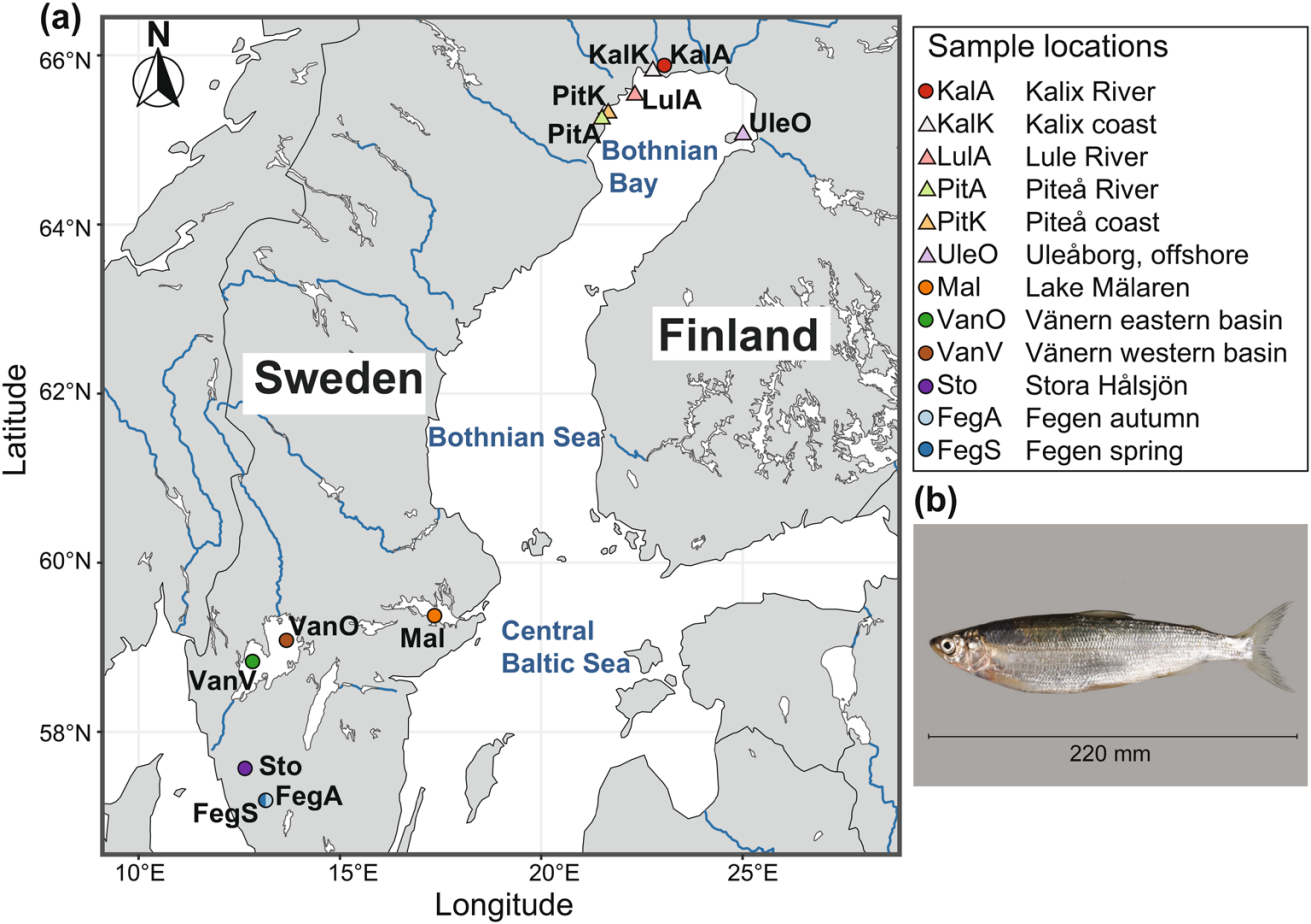
Sample information. (a) Sample locations. Geographic locations of the 12 European cisco population samples used in the study. In Lake Fegen, two distinct populations were sampled: spring‐ and autumn‐spawning populations. Triangle and circle represent brackish and freshwater populations, respectively. The map was derived from open‐source maps available in the rnaturalearth (R package: https://github.com/ropensci/rnaturalearth). (b) Image of a European cisco individual from Lake Mälaren. Photo: Bo Delling.

Here, we provide a high‐quality reference genome of the European cisco and low‐coverage whole‐genome sequencing data of 336 individuals from 12 populations spanning from south‐central freshwater lakes in Sweden to the Bothnian Bay area (Figure 1, Table S1). This study represents a major contribution to available genomic resources in European cisco and reveals a large number of loci showing genetic differentiation between populations adapted to fresh and brackish water conditions as well as between spring and autumn spawners.

## 2 Methods

### 2.1 Sample Collection

Details on samples and populations analysed in this study are given in Table S1 and Figure 1. All individuals are of Swedish origin except for one population (Uleåborg/Oulu population from the Finnish coast). We aimed to collect spawning fish to ensure that samples could provide a valid baseline. The reference individual for genome sequencing was a male individual from Lake Mälaren (Sweden).

### 2.2 Genome Assembly

#### 2.2.1 Preparation of High Molecular Weight (HMW) DNA

HMW DNA from spleen tissue was extracted using the Monarch HMW DNA Extraction Kit for Tissue (NEB, #T3060S/L) following the protocol described in the instruction manual (Version 1.0_10/20). Extracted DNA was subject to a clean‐up procedure using phenol:chloroform:isoamylalcohol (25:24:1) in MaXtract High Density phase lock tubes (Qiagen Cat #129056). DNA was then purified with chloroform:isoamylalcohol (24:1) and a high‐salt/low ethanol precipitation using 0.3× vol of 99% ethanol, which precipitates polysaccharides while gDNA remains in solution. DNA was precipitated using 1.7× vol 99% ethanol, washed twice with 70% ethanol, and eluted in low TE‐buffer (10 mM Tris, 0.1 mM EDTA). Additional DNA from brain tissue of the same individual was extracted using the manual Smart DNA prep kit (Analytical Jena, Cat #845‐KS‐80000109) according to the manufacturer's instructions (Issue 02/2018).

#### 2.2.2 RNA Preparation

RNA was extracted from cryo‐ground *Coregonus albula* tissues using the TRIzol Reagent and Phasemaker Tubes Complete System (Invitrogen Cat #A33250) following the Invitrogen user guide (Pub. No. MAN0016163 Rev. A.0) except for steps 2a and 3d, which were omitted. RNA was resuspended in 87.5 μL RNase‐free water and immediately subjected to DNase treatment followed by purification according to the RNeasy Micro Handbook (pages 74 and 53). The eluted RNA was stored at −70°C until processed further.

#### 2.2.3 PacBio HiFi Sequencing

In total 5 PacBio SMRTbell libraries were constructed using the SMRTbell Express Template Prep Kit 2.0 & SMRTbell Enzyme Clean up Kit following the instructions described in ‘Procedure & Checklist—Preparing HiFi SMRTbell Libraries using SMRTbell Express Template Prep Kit 2.0’ (PN 101–853‐100 Version 03 [January 2020]). In brief, genomic DNA was sheared into ~20 kb fragments using the Megaruptor 3 (Diagenode). After removal of ssDNA overhangs, DNA damage repair and end‐repair/A‐tailing, DNA fragments were ligated to hair‐pin adaptors to generate SMRTbell libraries for circular consensus sequencing. The libraries were then subjected to nuclease treatment before they were size selected using the SageELF system (SageScience). The fractions with suitable fragment length obtained during size selection went on to sequencing. Primer annealing and polymerase binding was performed using the Sequel II binding kit 2.0 and Sequencing Primer v2. In total 9 Sequel SMRT Cells 8 M v3 were sequenced either on the Sequel II or Sequel IIe system, using Sequel II Sequencing Plate 2.0, On‐Plate Loading Concentration of 80–150 pM, movie time 24 h and pre‐extension time 2 h.

#### 2.2.4 Iso‐Seq Sequencing

RNA extracted from spleen, brain, retinae, skin, liver, gills and testis was pooled equimolarly into one sample of which one IsoSeq SMRTbell library was prepared as described in ‘Procedure & Checklist—Iso‐Seq Express Template Preparation for Sequel and Sequel II Systems’ (PN 101–763‐800 Version 02, October 2019) using the NEBNext Single Cell/Low Input cDNA Synthesis & Amplification Module (New England Biolabs), the Iso‐Seq Express Oligo Kit (PacBio), ProNex beads (Promega) and the SMRTbell Express Template Prep Kit 2.0 (PacBio). 300 ng of total RNA was used for cDNA Synthesis followed by 12 cycles of cDNA Amplification. In the purification step of the amplified cDNA, the standard workflow was applied (sample is composed primarily of transcripts centered around 2 kb). After purification the amplified cDNA went into SMRTbell library construction. Primer annealing and polymerase binding was performed using the Sequel II binding kit 2.0 and Sequencing Primer v4. Finally, two Sequel SMRT Cells 8 M v3 were sequenced, one on Sequel II and one on Sequel IIe using Sequel II Sequencing Plate 2.0, On‐Plate Loading Concentration of 80 pM, movie time 24 h and pre‐extension time 2 h.

#### 2.2.5 Omni‐C

35 mg of frozen, grounded liver tissue was defrosted and crosslinked for 10 min in 3 mM disuccinimidyl glutarate (DSG), followed by an additional crosslinking of 10 min in 1% Formaldehyde. A 1 mL syringe and a 200 μM/50 μM filter were used to remove debris from tissue. The digestion conditions for the chromatin were determined by titration of the kit‐provided Nuclease Enzyme Mix. Proximity ligation, consisting of End‐Polishing, Bridge Ligation, Intra‐Aggregate ligation and Crosslink Reversal, was performed as described by the supplier using the Omni‐C Kit (Dovetail, DG‐REF‐001, DG‐REF‐002). The chromatin was purified using AMPure XP beads (Beckman Coulter, A63882). 150 ng of proximity‐ligated chromatin was used as input for library preparation using reagents from NEBNext Ultra II DNA Library prep kit for Illumina (NEB, E745S). The indexing PCR step used NEBNext Multiplex Oligos for Illumina Index Primers Set 1 (NEB, E7335S) and indexed libraries were subjected to 12 amplification cycles prior to DNA cleanup using AMPure XP beads. The final libraries were analysed for fragment length and concentration using a Bioanalyzer DNA high sensitivity chip and Qubit high sensitivity dsDNA. Samples were sequenced on an Illumina NovaSeq6000 with a 151 nt(Read1)‐10 nt(Index1)‐10 nt(Index2)‐151 nt(Read2) setup using the ‘NovaSeqXp’ workflow in ‘S4’ mode flowcell. Sequencing yielded 1323 M read‐pairs of raw data. Bcl to FastQ conversion was performed using bcl2fastq_v2.20.0.422 from the CASAVA software suite. The quality scale used was Sanger/phred33/Illumina 1.8+.

#### 2.2.6 De Novo Assembly and Scaffolding

Assembly was carried out with Hifiasm v0.16.1 (Cheng et al. 2021) and haplotypic duplications were identified and removed with purge_dups (Guan et al. 2020). For pre‐processing of OmniC reads, the ‘mapping_pipeline’ from Arima Genomics (v. ‘02/08/2019’) (https://github.com/ArimaGenomics/mapping_pipeline) was used. It entails mapping of reads to the contig assembly and read deduplication and filtering. A draft scaffolding was generated using YaHS (v. ‘1.2a.1.patch’) (Zhou et al. 2023) and further refined manually, that is, manually curated, using Juicebox (v. 1.11.08; Durand et al. 2016) and finalised using the YaHS ‘juicer post’ command.

The degree of synteny between our assembly and three previously reported *Coregonus* assemblies were analysed using Circos plots created with JupiterPlot (v1.1, https://github.com/JustinChu/JupiterPlot) using the following arguments: ‘minBundleSize = 400,000, gScaff = 1, maxGap = 400,000, ng = 0, labels = both’.

#### 2.2.7 Manual Curation

Files for manual curation were generated using the Earth‐Biogenome‐Project‐pilot nextflow pipeline (Binzer‐Panchal et al. 2024). Manual curation was performed using HiGlass (v1.13.0) (Kerpedjiev et al. 2018), PretextView (0.2.5) (Harry 2022) and the rapid curation tools (GitLab 2024).

#### 2.2.8 Genome Annotation

Genome annotation relies heavily on high‐quality evidence data. In this study, protein sequences were obtained from the UniProt Swiss‐Prot database (568,363 proteins) (UniProt Consortium 2023), along with additional datasets specific to Salmoniformes including Atlantic salmon ( *Salmo salar* ) (14,121 and 42,182 proteins, respectively). RNA‐seq data of gills, liver, skin and spleen from the IsoSeq sequencing were assembled using fastp (Chen et al. 2018) (v0.23.2), HISAT2 (Kim et al. 2015) (v2.1.0) and StringTie (Pertea et al. 2015) (v2.2.1), following a Nextflow (Di Tommaso et al. 2017) (v22.10.1) in‐house pipeline (Binzer‐Panchal et al. 2021).

A species‐specific repeat library was constructed using the RepeatModeler package (Flynn et al. 2020) (v2.0.2a) to exclude any nucleotide motif stemming from low‐complexity coding sequences. Repeat sequences were identified using RepeatMasker (Smit et al. 2013) (v4.1.2_p1) and RepeatRunner (Smith et al. 2007) to analyse highly divergent repeats and retro‐element coding regions.

Gene builds were computed using the MAKER pipeline (Holt and Yandell 2011) (v3.01.02). The gene annotation process consisted of two primary steps: (1) an evidence‐based build, where transcript alignments and reference proteins were used to generate consensus gene structures, and (2) an ab initio build, which leveraged evidence alignments along with a curated ab initio profile. Augustus (Stanke et al. 2006) was trained for ab initio gene prediction using an in‐house pipeline (Binzer‐Panchal et al. 2021).

Functional annotation of genes and transcripts was performed using the translated CDS features of each coding transcript. BLAST (Altschul et al. 1990) (v2.9.0) and InterProScan (Jones et al. 2014) (v5.59–91.0) were used to infer canonical protein names and functional predictions, with the results parsed and reconciled into a final set of annotations using an in‐house pipeline (Binzer‐Panchal et al. 2021).

The final annotation was evaluated using an in‐house Perl script (Dainat et al. 2023). To improve the quality of gene models, evidence‐based and ab initio annotations are usually combined, using one as the base and supplementing it with the other for loci missing in the base set. However, in this case, combining the runs did not lead to improvements in the annotation, and as it was the best one of the two, the ab initio annotation was retained as the final version. Genes without functional annotations (i.e., without domain or name) were removed from the final annotation, provided that this did not affect BUSCO scores (Waterhouse et al. 2018).

### 2.3 Population Genomics

#### 2.3.1 Low Coverage Whole Genome Sequencing

Genomic DNA was extracted with the DNeasy 96 Blood and Tissue kit (QIAGEN GmbH, Hilden, Germany). DNA concentration and purity were assessed with the Nanodrop 1000; DNA integrity was checked by running a 2 μL aliquot of each sample on an agarose gel. The DNA was then diluted to a final concentration of 10 ng/μL. Libraries for whole genome sequencing were prepared with a tagmentation approach as previously described (Picelli et al. 2014; Goodall et al. 2024). Briefly, Tn5 transposase (Tn5 Tnp) was used to fragment gDNA and tag it with mosaic end primers, subsequently used to append Illumina indexes with a combinatorial barcoding strategy. Since the chemistry of sequencing consisted of paired‐end sequencing with a read length of 150 bp, several rounds of optimization were performed to find the reaction conditions which gave a peak insert size of 300 bp. The conditions that were screened were reaction volumes, Tn5 Tnp concentration, DNA quantity, tagmentation time, PCR enzyme and library size selection strategy. The final tagmentation reactions were carried out for 7 min at +55°C in a total volume of 20 μL with 20 ng of gDNA template and 192 ng of Tn5 Tnp. The limited‐cycle PCR had a total volume of 50 μL, of which 25 μL were from the previous tagmentation reaction (20 μL tagmentation +5 μL 0.2% SDS), 20 μL from the PCR mix and 5 μL from the unique combination of i5 and i7 Illumina indexes from the Illumina Nextera Library Prep Kits for each library sample. Both the KAPA HiFi PCR Kit and the KAPA HiFi HotStart PCR Kit (Kapa Biosystems Pty, Cape Town, South Africa) were used, with the latter kit giving slightly longer insert sizes compared to the former. The AMPure XP paramagnetic beads (Beckman Coulter Inc., Brea, California, USA) were used to purify the libraries and enrich for the desired size. Since the mosaic end primers and Illumina indexes add 137 bp to the DNA fragments originating from the native sample, libraries with peaks of at least 440 bp were considered satisfactory. A double‐sided size selection was performed according to Bruinsma et al. (2018), and two samples of each cisco population were analysed at the TapeStation 4150 (Agilent Technologies, Waldbronn, Germany). The bulk of individual libraries were checked for concentration with the TECAN infinite M200 microplate reader (Tecan Austria GmbH, Grödig, Austria) employing the Qubit HS reagents (Life Technologies Corporation, Eugene, Oregon). Libraries were then pooled and the concentration of the pools was measured with the KAPA Library Quant Kit (Kapa Biosystems Pty, Cape Town, South Africa). The sequencing was performed in Uppsala by the SNP and SEQ Technology Platform by using three lanes of an S4 flow cell with a NovaSeq 6000 instrument.

#### 2.3.2 Read Mapping

Before mapping, we trimmed potential adapter contamination and assessed raw data quality using Trimmomatic v0.39 (Bolger et al. 2014). We aligned the trimmed data to the European cisco reference genome using ‘bwa mem‐M’ v0.7.15 (Li 2013) with default settings. We deduplicated and clipped overlapping read pairs using the MarkDuplicates from PicardTools v1.92 (https://broadinstitute.github.io/picard/) and ‘bam clipOverlap’ command from bamUtil v1.0.15 (Jun et al. 2015). We realigned indels processed with ‘RealignerTargetCreator’ and ‘IndelRealigner’ from GATK 3.8–0. We sorted and indexed the resulting bam alignment files using Samtools v1.12 (Li et al. 2009).

#### 2.3.3 Genotype Likelihood Estimation and Minor Allele Frequency Cutoff

Given that genomic complexity combined with low to intermediate sequencing depth can lead to mismapping and spurious SNP calls that distort population genetic estimates (Dallaire et al. 2023), we first examined the depth distribution using ANGSD ‘‐doQsDist 1 ‐doDepth 1 ‐doCounts 1’. Depth filter thresholds were set to average depth ±50% (from 0.5× to 1.5× the average sequencing depth). We used ANGSD v0.933 (Korneliussen et al. 2014) to estimate genotype likelihoods for all analyses, because our low sequencing coverage approach prohibits calling genotypes. Instead, we used genotype likelihoods to estimate population allele frequencies (based on 18 to 30 individuals per location; Table S1). We used the following parameters for all runs:

‘‐uniqueOnly 1 ‐remove_bads 1 ‐ only_proper_pairs 0 ‐trim 0 ‐GL 2.’ Depending on the analysis, we used the ‘‐doMajorMinor 4 ‐minMaf 0.05’ combination to generate lists of positions with minor allele frequency (MAF) > 5%, for all chromosomes and scaffolds. These were then supplied as the ‘‐sites’ arguments for genotype likelihood, and population‐wise allele frequency calculations, while diversity, Tajima's *D* and *d*_*xy*_ calculation used all observed positions.

#### 2.3.4 Population Structure

To investigate genome‐wide differentiation patterns among and within populations, we divided all samples into different contrasting groups based on geographical distance, spawning season and ecotypes. To control for linkage disequilibrium (LD), we used PLINK 1.9 to perform LD pruning by removing SNPs with a variance inflation factor greater than two (VIF > 2) in 100‐SNP sliding windows shifted by five SNPs per iteration. The resulting LD‐pruned SNP set was subsequently used for downstream analyses including PCA, admixture and selection scans. We performed a principal components analysis (PCA) of sequence variation for each contrasting group using PCAngsd v0.982 (Meisner and Albrechtsen 2018). We ran PCAngsd using a downsampled list of 22.9 million sites (using the MAF > 0.05 filter) and supplied the ‐SNP_pval 1e^−6^ to ANGSD to include only high‐confidence variants. We ran PCAngsd to infer admixture proportions using the ‐admix option; PCAngsd can automatically infer the most likely number of ancestry clusters. Additionally, we manually set the number of clusters to range from 2 to 10 using the ‐admix_K option. The results obtained for the best K using ‐admix_K and the automatic inference were qualitatively consistent. Only the results corresponding to the best K are presented for population structure analysis. The covariance matrix output by PCAngsd was utilised to visualise principal components, and eigenvectors were calculated for the covariance matrix using the eigen function in R v4.3.1 (R Core Team 2023).

The frequencies for each contrast component were calculated using ANGSD ‘–doMajorMinor 4’ on the sites that exceeded the study‐wide 5% MAF threshold (see above). This implies we cannot detect differentiation that occurs exclusively in rare SNPs with a frequency below this threshold.

#### 2.3.5 Genome‐Wide Screen for Genetic Differentiation

We performed this screen to identify SNPs showing genetic differentiation in different contrasts. We estimated average allele frequencies in different population groups, using genotype likelihoods, and tested for genetic differentiation in four contrasts: (a) Lake Fegen and Stora Hålsjön vs. all other population samples. (b) Spring spawners vs. autumn spawners in Lakes Fegen and Stora Hålsjön. (c) freshwater Kalix River vs. all other population samples from the Bothnian Bay area (riverine and coastal). (d) freshwater lakes Vänern + Mälaren vs. population samples from the Bothnian Bay area (riverine and coastal) after excluding Kalix River. We calculated *p* values for each contrast using the following command:

angsd ‐yBin $GWAS_DIR/$BAM_TARGET.ybin ‐doAsso 1 ‐out $OUT_DIR/${OUTPUT}_${CHUNK_ID} ‐ref $REFGENOME ‐fai $REF_INDEXED ‐doMajorMinor 4 ‐doMaf 1 ‐bam $GWAS_DIR/$GWAS_NAMES ‐nInd x ‐gl 2 ‐sites $SITES ‐nThreads 8.

We applied Bonferroni correction (alpha = 0.05) to control for multiple SNP tests, calculated separately for each analysis. We set the threshold for calling outlier SNPs at the −log_10_(*p*) value corresponding to the top 0.01% (Figure S1). Only genes containing SNPs that exceed the significance threshold (top 0.01%, as shown in Figures S2–S5) were highlighted as candidate genes. We performed a genome‐wide compilation of independent signals of differentiation (Figures S2–S5), defined as having at least a 500 kb gap to serve as a conservative estimate of the minimum number of independent signals to avoid splitting a single signal.

#### 2.3.6 Nucleotide Diversity

We calculated thetas using allele frequencies calculated from a call set that was not filtered for MAF cutoffs, because any MAF cutoff will bias site frequency spectrum‐based diversity estimates. We first estimated the average minimum and maximum sequencing depths (individual min_depth and max_depth) per sample based on depth filter statistics across all individuals. The sequencing depth thresholds for each group were then calculated by multiplying these individual values by the number of individuals in the group (i.e., MINDEPTH = individual min_depth × number of individuals; MAXDEPTH = individual max_depth × number of individuals). We calculated sample allele frequencies per sampling locality using the following command:

angsd ‐bam $POP ‐anc $REFGENOME ‐fai $REF_INDEXED ‐rf $CHUNK_DIR/$CHUNK_NAMES ‐doSaf 1 ‐GL 1 ‐P 8 ‐doCounts 1 ‐setMinDepth $MINDEPTH ‐setMaxDepth $MAXDEPTH ‐setMinDepthInd 0.25 ‐minMapQ 30 ‐minQ 20 ‐remove_bads 1 ‐minInd 10 ‐skipTriallelic 1 ‐uniqueOnly 1 ‐dumpCounts 2 ‐doMaf 1 ‐doMajorMinor 1 ‐out $OUT_DIR/${OUTPUT}_${CHUNK_ID}.

Our filters were selected to remove extreme outliers in sequencing depth and to remove spurious alignments and low‐quality sites. Next, we used the ANGSD realSFS command to generate the folded SFS by supplying the ‐anc with the reference genome and applying ‐fold 1 to realSFS. Pairwise nucleotide diversity was calculated in 10‐kb non‐overlapping windows and averaged per population, divided by the number of sites per window to recover an unbiased diversity estimate.

For *F*_ST_, we estimated the 2D SFS using the command ‘realSFS’ and employed it as a prior to calculate *F*_ST_ for each variable site using the commands ‘realSFS fst’ and ‘realSFS print’ and custom scripts (see ‘Code Availability’ for access to all custom scripts), and in 10‐kb moving windows with the command ‘realSFS fst stats2.’ We calculated the average *F*_ST_ for the first four chromosomes to represent the overall genome, in order to save memory and reduce computational load.

To characterise the genomic landscape of differentiation among populations, we calculated the estimated absolute sequence divergence (*d*_*xy*_) (Nei and Li 1979; Burri 2017). *d*_*xy*_ measures genetic sequence divergence between populations and indicates whether populations are more or less diverse compared to each other. We used the 2d‐SFS as input to calculate *d*_*xy*_ using a public python script (https://github.com/ivanliu3/asfsp).

#### 2.3.7 Functional Annotation of Associated Genes

To understand the genetic basis of reproductive behaviour and salinity adaptation, we first identified outlier windows from population contrasts, with SNPs exceeding the significance threshold (Figure S1) with BEDTools v2.29.2 (Quinlan and Hall 2010). We determined whether the SNPs fell within genes including 5 kb upstream and 3 kb downstream of gene sequences; otherwise, they were denoted as intergenic. This approach is based on the expectation that sequence alterations can directly impact phenotypic variation by modifying protein sequences or indirectly by influencing the expression of nearby genes, usually the closest ones. Next, we extracted the protein sequences of all associated genes using SeqKit/2.4.0 (Shen et al. 2024). Lastly, all protein sequences were annotated, and a Gene Ontology enrichment analysis was carried out using the online tool KOBAS (http://bioinfo.org/kobas) (Bu et al. 2021).

## 3 Results

### 3.1 Genome Assembly and Annotation

We generated 137 Gb of sequencing data from 9 PacBio HiFi SMRT cells and 399 Gb of Hi‐C data (Figure S6) to construct a high‐quality genome assembly. The final assembly comprised 1574 scaffolds with a scaffold N50 of 54.8 Mb and a total assembly size of 2540 Mb (Table 1). More than 89% of the assembled genome could be assigned to the 40 chromosomes (Figure S6), indicating a high level of structural completeness. The quality of the assembly was validated using Merqury (Rhie et al. 2020), which estimated a quality value (QV) of 53.8, corresponding to approximately four errors per megabase. Despite the overall high accuracy, the assembly posed challenges due to the polyploid nature of the organism. Haplotype phasing in the contig assembly was suboptimal, leading to residual haplotypic duplications. To address these issues, extensive manual curation was performed, requiring an average of 276 interventions per gigabase. The genome assembly's completeness was further evaluated using BUSCO, revealing that 97.7% of the 3640 BUSCO groups were complete, with 53.1% being single‐copy and 44.6% duplicated. Only 0.9% of the BUSCO groups were fragmented, and 1.4% were missing. The GC content of the assembly was 44.0%. This high‐quality assembly serves as a valuable resource for further genomic studies.

**TABLE 1:** Genome assembly metrics for *Coregonus albula* , fCorAlb1.

**Assembly metrics**	
Number of scaffolds	1574
Scaffold N50 (Mb)	54.8
Scaffold L50	19
Number of contigs	7994
Contig N50 (Kb)	850
Contig L50	683
Total size (Mb)	2540
GC content (%)	44.0
% assigned to chromosomes	89.6
**BUSCO analysis**	
Complete (%)	97.7
Complete and single‐copy (%)	53.1
Complete and duplicated (%)	44.6
Fragmented (%)	0.9
Missing (%)	1.4
Total BUSCO groups	3640
**Merqury analysis**	
Quality value	53.78
Completeness	84.30

*Note:* BUSCO scores based on the actinopterygii_odb10 lineage (creation date: 05‐Dec‐2024) and BUSCO version v5.3.2.

We performed assembly quality control based on read depth analysis as previously described (De‐Kayne et al. 2020). We mapped all short reads from whole genome sequencing of 336 samples (next section) to the reference assembly (Figure S7). This analysis revealed that chromosomes 34, 36 and 37 have a two‐fold higher sequence coverage compared with the rest of the genome, strongly suggesting that these represent collapsed tetrasomic regions of the genome. This information is important to take into account when interpreting population data generated using this assembly.

We compared the degree of synteny between our assembly and three previously reported *Coregonus* spp. genome assemblies (*Coregonus* sp. ‘Balchen’, *C. clupeaformis* and *C. artedi* ) (De‐Kayne et al. 2020; Mérot et al. 2023; Backenstose et al. 2024) using Circos plots (Figures S8–S10). The majority of the chromosomes show a high degree of similarity of chromosome structure across the four assemblies. However, more pronounced differences were noted on chromosomes 30, 34, 36 and 37. The result for the latter three is most likely related to the aforementioned collapsed tetrasomic regions in the *Coregonus albula* genome. In fact, chromosomes 34, 36 and 37 in our assembly correspond to scaffolds 32, 38 and 22, respectively, in the assembly of *Coregonus* sp. ‘Balchen’ and these three scaffolds were also identified as collapsed tetrasomic chromosomes (De‐Kayne et al. 2020). In contrast, the corresponding regions of two other scaffolds in the *Coregonus* sp. ‘Balchen’ assembly, scaffold 28 and 36, reported as collapsed duplicated chromosomes (De‐Kayne et al. 2020), did not show elevated sequence coverage in the *Coregonus albula* genome (parts of chromosomes 27 and 30, and chromosome 22; Figures S7 and S8).

MAKER was employed to annotate repeats and genes in the *Coregonus albula* assembly (see Section 2). Repeat masking identified 2,872,454 repeats, which cover 56.6% of the genome. The majority of these repeats were TC1/Mariner (17.2%), simple repeats, or classified as unknown. In comparison, RepeatRunner, utilising the MAKER TE library, detected 61,907 repeats, covering only 1.9% of the genome.

The final gene annotation identified 51,040 genes and 125,922 mRNAs. BUSCO analysis of the annotation revealed that 89.8% of BUSCOs were complete, as expected, a lower percentage compared with BUSCO results for the genome assembly before annotation (Table 1). Of these, 36% were duplicated, while 3.3% were fragmented and 6.9% were missing. Functional annotation linked 99% of the genes and 98% of the mRNAs to protein domains across multiple databases, including Pfam, CDD and Gene3D. Gene annotation resulted in 83% of genes and 87% of mRNAs being assigned a gene name (Table S2); almost all of these had an open reading frame exceeding 200 bp. Out of those genes, 4201 were single exon genes and the other 46,839 genes have a mean of 10.2 exons per gene. The interquartile range (IQR) of gene lengths, corresponding to the middle 50% of values, spanned from 2415 to 22,882.75 base pairs (Figure S11). We functionally annotated genes using InterProScan version 91.0 (against all 21 databases available) and used blast to find gene names.

The initial gene count was higher than expected. To address this, genes without functional annotations (i.e., domains or names from interproscan or blast) were removed from the final dataset, an action that did not affect the BUSCO results. Only 571 single genes were removed out of the 8856 genes removed, and none of the exons had any functional annotation at all. The final gene count is slightly higher than anticipated for this species. Salmonid species typically have between 40,000 and 50,000 genes, as shown in Table 2. The gene count observed in *Coregonus albula* is not unexpected, given the potential for some remaining duplications in the final assembly and possible overpredictions during the annotation process.

**TABLE 2:** Gene counts in genome assemblies for various salmonids (Ensembl).

Species	Version name	No. of genes
European cisco ( *Coregonus albula* )	fCorAlb1	51,040
Atlantic salmon ( *Salmo salar* )	Ssal_v3.1	47,205
Brown trout ( *Salmo trutta* )	fSalTru1.1	43,935
Coho salmon ( *Oncorhynchus kisutch* )	Okis_V2	43,940
Rainbow trout ( *Oncorhynchus mykiss* )	USDA_OmykA_1.1	48,326
Danube salmon ( *Hucho hucho* )	ASM331708v1	50,114

### 3.2 Low‐Coverage Whole Genome Sequencing

Low‐coverage whole‐genome sequencing produced 7 billion reads across 336 individuals (Figure S12). The average genome depth of mapped reads after quality filtering was 1.08× (Table S1). We called variants across 2,572,320,831 sites (including variant and invariant sites). After quality filtering and SNP scoring, we identified 22,916,748 biallelic SNPs among all 336 individual samples. After depth filtering (Figure S13) and applying a minor allele frequency (MAF) filter, 12,463,256 SNPs were retained. Following linkage disequilibrium (LD) pruning, 3,307,760 SNPs remained for downstream PCA and Admixture analyses.

Population genomic statistics revealed moderate nucleotide diversity within populations, *π*, in the range of 0.0028–0.0050 (Table S1). The individuals from the Lule River exhibited the highest diversity (*π* = 0.0050), closely followed by other samples from the Bothnian Bay area (riverine and coastal). Lowest diversities were exhibited by Stora Hålsjön (one population; *π* = 0.0028) and Lake Fegen (two sympatric populations; *π* = 0.0030 and 0.0031), whereas Lake Vänern and Mälaren displayed intermediate levels of nucleotide diversity (Table S1).

### 3.3 Detection of Population Structure

**FIGURE 2:**
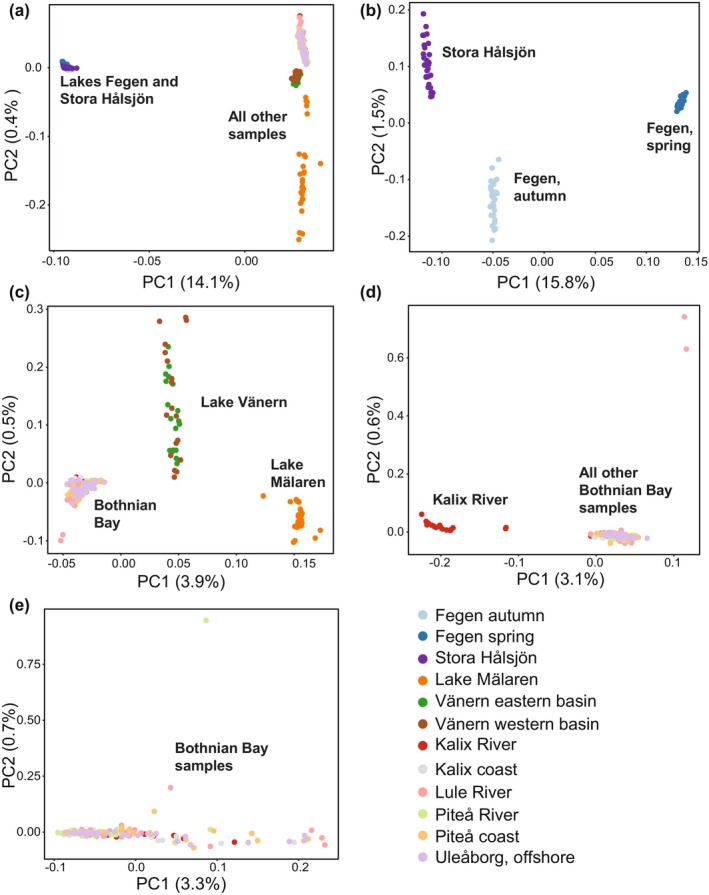
Principal component analysis of European cisco populations. (a) Based on all populations. (b) Using only samples from Lakes Fegen and Stora Hålsjön. (c) All other samples after excluding those from Lakes Fegen and Stora Hålsjön. (d) Using only Bothnian Bay samples. The two points in the upper right corner are two of the samples from Lule River. (e) Using all Bothnian Bay samples after excluding 21 samples from Kalix River that stand out in panel (d) and two samples from Lule River (upper right corner in panel d).

Principal component analysis (PCA) using ANGSD v0.933 (Korneliussen et al. 2014) based on genotype likelihoods of all called SNPs revealed distinct patterns of genetic differentiation among European cisco populations in Sweden (Figure 2). The primary division was observed between Lake Fegen and Stora Hålsjön populations in south‐west Sweden and all other populations (Figure 2a). A discernible population structure between spring‐ and autumn‐spawning populations from Lake Fegen and Stora Hålsjön was also noted; together, PC1 and PC2 explained 17.3% of the variation among all individuals (Figure 2b). These patterns were further supported by Admixture analysis (Figure S14).

After excluding the population samples from Lake Fegen and Stora Hålsjön, a clear distinction was noted between samples from freshwater lakes in south‐central Sweden (Lake Vänern and Lake Mälaren) and the Bothnian Bay region in Northern Sweden and Finland (Figure 2c). Within the latter region, a clear separation exists between most individuals from the freshwater location Kalix River and all other population samples from the Bothnian Bay area (riverine and coastal) forming a third cluster (Figure 2d). Nine out of 30 individuals from Kalix River grouped with the samples from the Bothnian Bay area, and these are referred to as ‘Kalix mix’ in the subsequent analysis. In addition, two out of 30 individuals from Lule River were far away from all other Bothnian Bay individuals in the PCA plot (Figure 2d), suggesting additional genetic heterogeneity in the region. These two samples were excluded from further analysis. We found no discernible genetic differentiation between samples from the Swedish and Finnish coast (Figure 2e), contrary to a previous result based on RAD‐seq that noted a minute genetic differentiation (*F*_ST_ = 0.05%–0.10% in pairwise comparisons), which still reached statistical significance (López et al. 2022).

The *d*_*xy*_ value between Lake Fegen/Stora Hålsjön and all other population samples was 0.5%, indicating moderate genetic divergence (Figure S15). The corresponding *F*_ST_ between these two groups reached 0.163 (16.3%), further indicating significant divergence between the two groups. The *F*_ST_ value between spring‐ and autumn‐spawning populations in Lake Fegen and Stora Hålsjön was 12.3%, reflecting strong genetic differentiation. In contrast, genetic differentiation among all other populations, after excluding Lake Fegen and Stora Hålsjön, was low, with an average *F*_ST_ ranging from 1.0% to 2.3% (Figure S15). The minor variation in *d*_*xy*_ indicates that these populations are all closely related. Thus, the relatively large variation among population comparisons in *F*_ST_ implies that genetic drift is a major factor behind those population contrasts with high genome‐wide *F*_ST_.

### 3.4 Genetic Signatures of Ecological Adaptation

To explore the genetic architecture underlying ecological adaptation in the European cisco, we formed super‐pools of populations representing the major groups detected using the PCA analysis (Figure 2) and performed the following genome‐wide contrasts: (i) populations from Lakes Fegen + Stora Hålsjön vs. all other population samples; (ii) spring spawners vs. autumn spawners in Lakes Fegen and Stora Hålsjön; and (iii) two distinct ‘fresh vs. brackish water’ contrasts, freshwater Kalix River vs. all other population samples from the Bothnian Bay area (riverine and coastal) as well as freshwater lakes Vänern + Mälaren vs. population samples from the Bothnian Bay area (riverine and coastal) after excluding Kalix River.

#### 3.4.1 Lakes Fegen + Stora Hålsjön vs. All Other Population Samples

**FIGURE 3:**
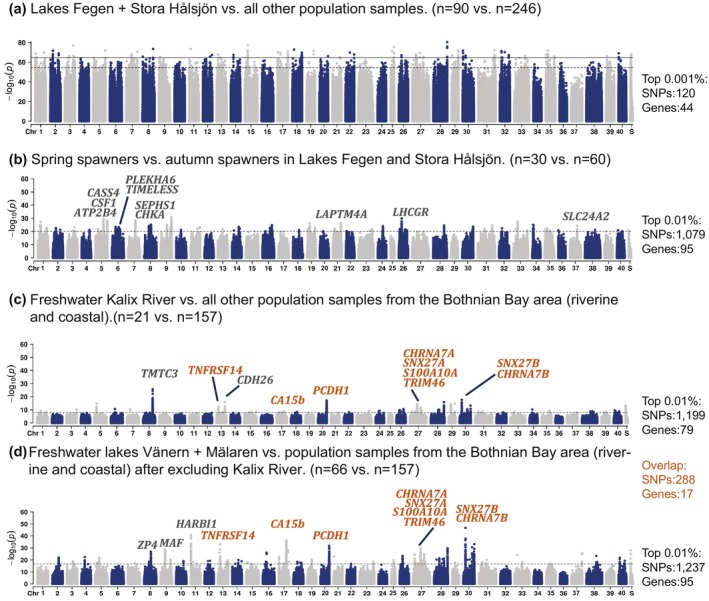
Genetic differentiation among European cisco populations revealed by genome‐wide SNP‐by‐SNP analysis. (a) Lakes Fegen + Stora Hålsjön vs. all other population samples. (b) Spring‐ vs. autumn‐spawners in Lakes Fegen and Stora Hålsjön. (c) Freshwater Kalix River vs. all other population samples from the Bothnian Bay area (riverine and coastal). (d) Freshwater lakes Vänern + Mälaren vs. population samples from the Bothnian Bay area (riverine and coastal) after excluding Kalix River. To the right we report the number of SNPs and associated genes exceeding the significance thresholds (data in orange refer to the overlap between (c) and (d)). The horizontal dashed black lines represent the top 0.01% of SNPs. The 0.01% threshold in panel (a) includes a lot of noise signals, so a 0.001% threshold, represented by solid black lines, was used instead. Detailed information on the −log_10_(*p*) thresholds for each contrast is summarised in Figure S1. ChrS represents unplaced scaffolds. The −log_10_(*p*) values were derived from genotype likelihoods in genome‐wide screen. Orange‐coloured gene symbols in (c) and (d) represent shared signals in the two contrasts.

The contrast between samples from Lake Fegen/Stora Hålsjön vs. all other population samples revealed strong genetic differentiation across the entire genome (Figure 3a and Figure S2), as expected from the PCA (Figure 2a). We recorded outlier loci reaching statistical significance after excluding regions consisting of a single SNP (Figure 3a; all highly differentiated SNPs are listed in File S1). The regions include a total of 120 high differentiation SNPs (−log_10_(*p*) > 64.5, top 0.001% of SNPs). We concluded that the two major groups of European cisco in Sweden are too differentiated across the whole genome to reveal clear signatures of selection for distinct genomic regions when analysed in a pair‐wise manner.

#### 3.4.2 Spring Spawners vs. Autumn Spawners in Lakes Fegen and Stora Hålsjön

To explore the genetic architecture underlying spawning time in European cisco, we formed super‐pools representing the genetically distinct spring‐ and autumn‐spawning populations. The contrast involved two autumn‐spawning populations from Lake Fegen and Stora Hålsjön and one spring‐spawning population from Fegen. There was a relatively high genome‐wide genetic differentiation between spawning ecotypes; however, a number of loci stood out as more genetically differentiated than the genomic background. We noted 1079 high‐differentiation SNPs (−log_10_(*p*) > 20.1; top 0.01% of SNPs), corresponding to at least 81 independent signals, located in the vicinity of 95 genes (Figure 3b and Figure S3).

One of the most significantly differentiated signals occurred on Chr37 between 39.3 and 39.4 Mb and contained a total of six significant SNPs in a region harbouring two genes, *SLC24A2* (solute carrier family 4 member 2) and *NANS* (*N*‐acetylneuraminic acid phosphate synthase) gene (Figure 3b and Figure S16). Another region on Chr37, around 37.5 Mb) approaching genome‐wide significance overlaps the *MTNR1A* (*Melatonin Receptor 1A*) gene. The top SNP is located in an intron of this gene (Chr37: 37,520,346 bp, −log_10_(*p*) = 19.3) (Figure S17). Melatonin plays a well‐defined role in regulating the circadian rhythm in vertebrates, as well as in photoperiodic regulation of reproduction (Slaugenhaupt et al. 1995; Takahashi and Ogiwara 2021).

**FIGURE 4:**
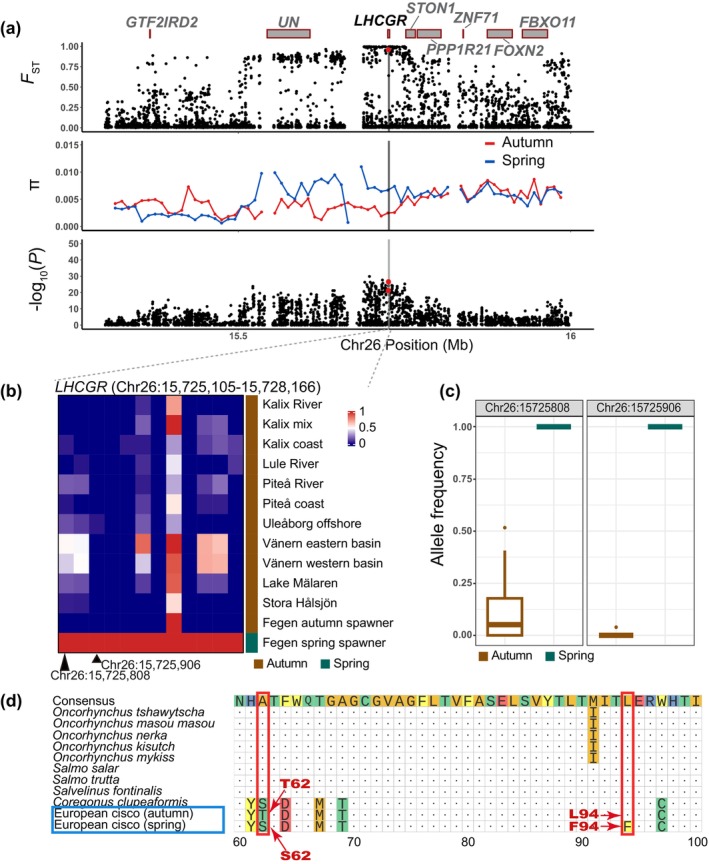
Examples of highly differentiated genes between spring spawners vs. autumn spawners in Lakes Fegen and Stora Hålsjön in European cisco—*LHCGR*. (a) Genome‐wide diversity statistics *F*_ST_, *π* and −log_10_(*p*) across the *LHCGR* locus on Chr26. *F*_ST_ and −log_10_(*p*) represent single SNP data while *π* is calculated for 10‐kb windows. The boxes with red borders indicate the genes surrounding the signals. The highlighted gene is the one closest to the most significant SNPs among the genes. ‘*UN*’ denotes an unannotated gene. The red dots in the −log_10_(*p*) plot indicate missense mutations. (b) Heatmap of allele frequencies across the *LHCGR* region. (c) Allele frequency for the two spawning groups at the missense mutation sites. (d) Amino acid sequence alignment of LHCGR. European cisco populations are highlighted in a blue box. The red dot positions in (a) are marked as the positions highlighted in (b) and (d).

Another highly divergent locus in the spring‐spawning vs. autumn‐spawning comparison occurs in a 10 kb region on Chr26 harbouring the *LHCGR* (luteinizing hormone/choriogonadotropin receptor) gene (Figures 3b and 4a). This signal includes two missense mutations (T62S and L94F), located in exon 3 of *LHCGR* (Chr26: 15,725,808 bp, −log_10_(*p*) = 21.3 and Chr26: 15,725,906 bp, −log_10_(*p*) = 26.6) (Figure 4a). The F94 allele is fixed in the single spring‐spawning population and occurs at a low frequency (0%–4%) in all autumn‐spawning populations (Figure 4b,c).

We performed a Gene Ontology (GO) enrichment analysis using the online tool KOBAS (http://bioinfo.org/kobas) of the 95 genes associated with genetic differentiation between spring‐ and autumn‐spawning populations, and noted significant enrichments (corrected *p* value < 0.01) for several GO categories (File S7). The visual perception category included five genes: *DRAM2* (Damage Regulated Autophagy Modulator 2) on chromosome 5, two copies of the *GM2* gene (Gamma‐crystallin M2) on chromosome 31, *GS‐1* (Gamma‐crystallin S‐1) on chromosome 31, and *SLC24A2* on chromosome 37 (Figure 3b and Figure S16). The circadian rhythm category is particularly interesting and included four genes, *TIMELESS* on chromosome 6 (Figure 3b and Figure S18), *BHLHE40* (*Basic Helix–Loop–Helix Family Member E40*) on chromosome 6, and two copies of *CPT1A* (*Carnitine Palmitoyltransferase 1A*) on chromosomes 7 and 8.

In addition to the typical bell‐shaped selective sweep signals, we noted three block‐like signals, showing a sharp change in divergence at the borders (highlighted with orange boxes in Figure S3). These block‐like patterns, suggesting suppressed recombination, are consistent with the presence of inversions or other structural rearrangements. All putative inversions are listed in Table 3.

**TABLE 3:** List of putative inversions in different contrasts between populations of European cisco.

Putative inversion	Contrast	Chromosome	Start (Mb)	End (Mb)	Size (Mb)
1	(b)	8	35.2	36.7	1.5
2	(b)	9	50.5	59.0	8.5
3	(b)	19	42.7	43.7	1.0
4	(c)	20	45.2	46.7	1.5
5	(c)	26	37.1	38.7	1.6
6	(d)	20	45.2	46.7	1.5
7	(d)	28	38.0	47.2	9.2

*Note:* (i) Contrast (b): Spring spawners vs. autumn spawners in Lakes Fegen and Stora Hålsjön. Contrast (c): freshwater Kalix River vs. all other population samples from the Bothnian Bay area (riverine and coastal). Contrast (d): freshwater lakes Vänern + Mälaren vs. population samples from the Bothnian Bay area (riverine and coastal) after excluding Kalix River. (ii) Putative inversions are highlighted with orange boxes in Figures S3–S5.

#### 3.4.3 Fresh vs. Brackish Water

This analysis involved two different contrasts—(c): freshwater Kalix River vs. all other population samples from the Bothnian Bay area (riverine and coastal) and (d): freshwater lakes Vänern + Mälaren vs. population samples from the Bothnian Bay area (riverine and coastal) after excluding Kalix River. The signals of genetic differentiation in the closely located freshwater Kalix River vs. Bothnian Bay contrast (Figures 1a and 3c) are cleaner than the contrast involving the freshwater lakes Vänern + Mälaren (Figure 3d), most likely because the latter contrast involves larger ecological differences and more genetic drift due to geographic isolation. In both these contrasts, an examination chromosome by chromosome shows that the signals of genetic differentiation are well above the background noise due to drift (Figures S4 and S5).

**FIGURE 5:**
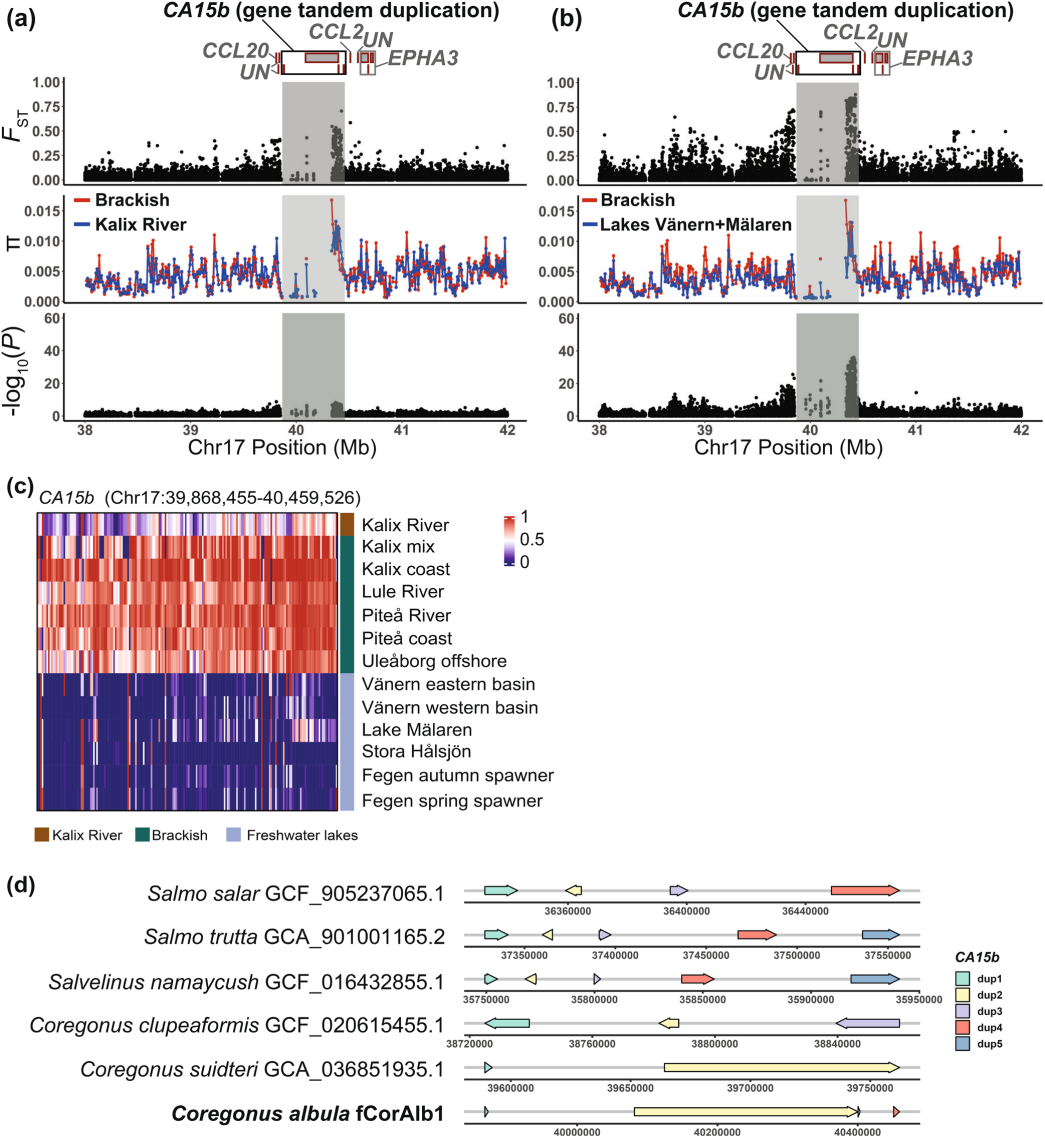
Example of shared signal of genetic differentiation in the vicinity of the *CA15b* locus in the two contrasts between Kalix River vs. Bothnian Bay and Lakes Vänern + Mälaren vs. Bothnian Bay in European cisco. (a, b) Genome‐wide diversity statistics *F*_ST_, *π* and −log_10_(*p*) across the *CA15b* locus on Chr17. *F*_ST_ and −log_10_(*p*) represent single SNP data while *π* is calculated for 10‐kb windows. The boxes with red borders indicate the genes surrounding the signals. ‘*UN*’ denotes unannotated genes. The highlighted gene is the ones closest to the most significant SNPs. (a) Freshwater Kalix River vs. all other population samples from the Bothnian Bay area (riverine and coastal). (b) Freshwater lakes Vänern + Mälaren vs. population samples from the Bothnian Bay area (riverine and coastal) after excluding Kalix River. (c) Heatmap of allele frequencies across the *CA15b* region. (d) Gene structure of *CA15b* tandem duplications in salmonids. European cisco ( *Coregonus albula* ) is highlighted in bold. Arrow direction represents the gene orientation. Visualisation of the gene structure was performed using R package ‘gggenes’ (Wilkins 2025). The gene structure information of other salmonids was retrieved from NCBI (https://www.ncbi.nlm.nih.gov/).

The contrast (c) freshwater Kalix River vs. all other population samples from the Bothnian Bay area (riverine and coastal) yielded 1199 high‐differentiation SNPs (−log_10_(*p*) > 7.8, top 0.01% of SNPs), corresponding to at least 75 independent signals, and associated with 79 genes (Figure 3c; all high‐differentiation SNPs and corresponding genes are listed in File S3). The contrast (d) freshwater lakes Vänern + Mälaren vs. population samples from the Bothnian Bay area (riverine and coastal) after excluding Kalix River yielded 1237 high‐differentiation SNPs (−log_10_(*p*) > 16.7, top 0.01% of SNPs), corresponding to at least 90 independent signals, and associated with 95 genes (Figure 3d; File S4). As many as 288 of these SNPs reached significance in both comparisons, a highly significant, non‐random overlap considering ~20 million SNPs were analysed in total (*P*_binomial test_ = 0). The 17 genes associated with these 288 overlapping SNPs are the best candidates for adaptation in the fresh vs. brackish water contrast (Files S5 and S6; Table S3). A prime example of such a locus is *CA15b* (*Carbonic Anhydrase15b*) on chromosome 17 (Figure 5). This gene shows a copy number expansion in salmonids, and we noted four *CA15b* copies in European cisco. This likely explains the reduced sequence coverage observed for part of this gene region (Figure 5a,b). One missense mutation was identified in copy 2 of *CA15b*, but this amino acid change occurred exclusively in a very short transcript isoform. In all other annotated transcripts, the variant was located in non‐coding regions. *Carbonic Anhydrase 15b* is required for the migration of primordial germ cells in developing embryos in zebrafish (Wang et al. 2013).

Other highly significant loci in the two fresh vs. brackish water contrasts include a putative inversion on Chr20 harbouring *PCDH1* (Protocadherin 1) and several other genes (Table 3, Figures S4, S5 and S19), and the closely linked genes *SNX27A* (Sorting Nexin 27A) and *CHRNA7A* (Cholinergic Receptor, Neuronal Nicotinic, Alpha Polypeptide 7B) on Chr27 (Figure S20) and their paralogs *SNX27B* and *CHRNA7B* on Chr30 (Figure S21), highlighted in Figure 3c,d. Further, *CHRNA7* has been predicted to be a target gene for a miRNA showing differential expression under salinity stress in sea cucumbers (Tian et al. 2019).

**FIGURE 6:**
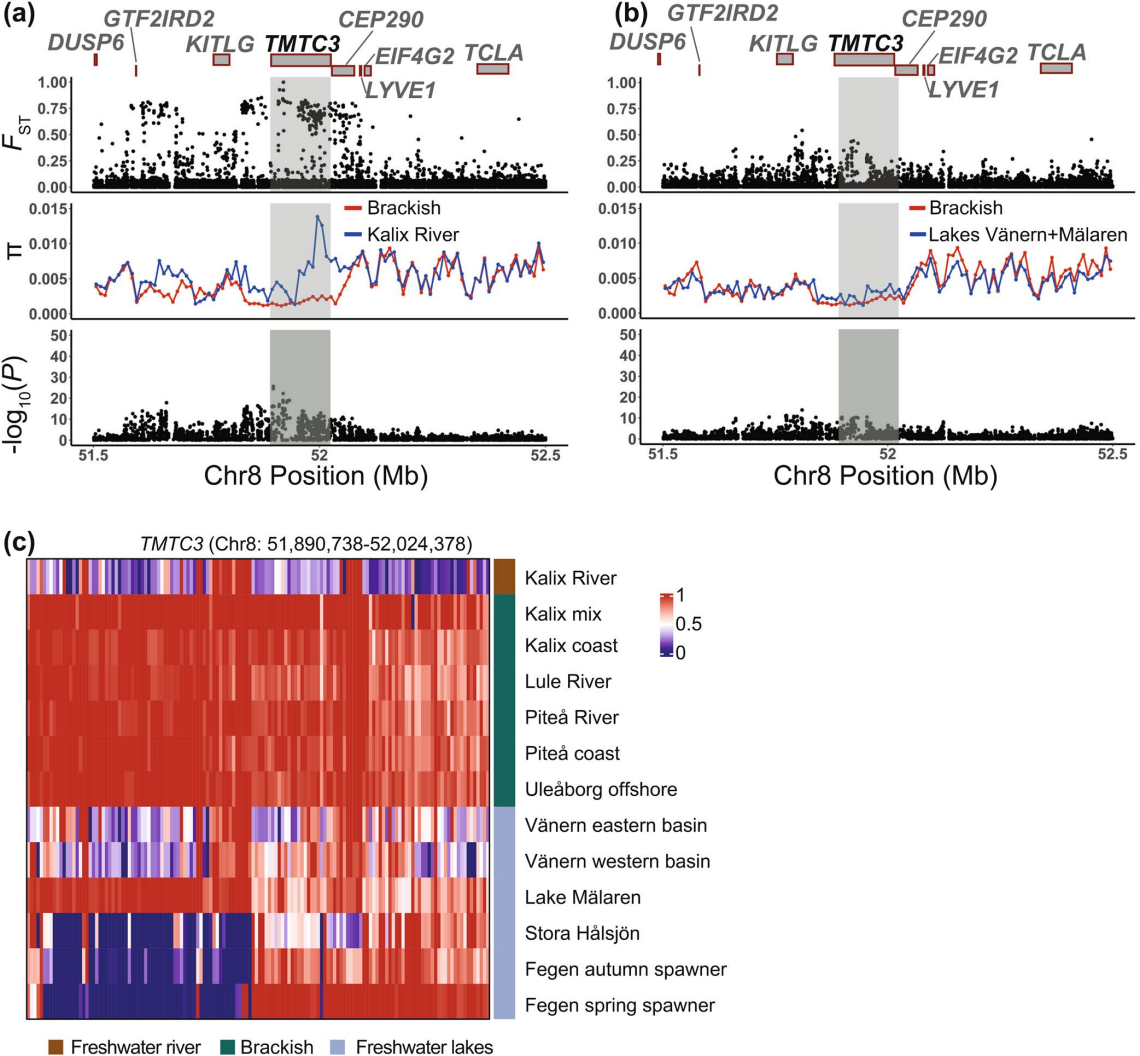
Examples of highly differentiated genes between contrast_c: Freshwater Kalix River vs. all other population samples from the Bothnian Bay area (riverine and coastal) in European cisco—*TMTC3*. (a) Genome‐wide diversity statistics *F*_ST_, *π* and −log_10_(*p*) across the *TMTC3* locus on Chr8. *F*_ST_ and −log_10_(*p*) represent single SNP data while *π* is calculated for 10‐kb windows. The boxes with red borders indicate the genes surrounding the signals. ‘*UN*’ denotes unannotated genes. The highlighted gene is the one closest to the most significant SNPs among the genes. (b) Heatmap of allele frequencies across the *TMTC3* region.

The loci that do not replicate in the two subsets are unlikely to be false positives but rather represent adaptation to local ecological conditions. A striking example of such a locus is the *TMTC3* (Transmembrane and tetratricopeptide repeat domains‐containing protein 3) locus on Chr8 that shows no genetic differentiation in the contrast (c) freshwater lakes Vänern + Mälaren vs. population samples from the Bothnian Bay area (Figures 3d and 6), but very strong genetic differentiation in the contrast (d) freshwater Kalix River vs. all other population samples from the Bothnian Bay area (riverine and coastal) (Chr8: 51,898,019; −log_10_(*p*) = 25.7; Figures 3c and 6). This signal is located within the intronic and 3′ untranslated regions (UTR) of *TMTC3*. *TMTC3* has an important role during saltmarsh adaptation in birds and an adaptive role in response to brackish water in fish (Dennenmoser et al. 2017; Walsh et al. 2018). Besides *TMTC3*, we noted several other highly significant loci only identified in the contrast (c) freshwater Kalix River vs. all other population samples from the Bothnian Bay area. The most significant SNP on Chr13 (−log_10_(*p*) = 16.0; Figure 3c) is located in the intronic region of Cadherin 26 (*CDH26*) (Figure 3c and Figure S22), a gene that has been associated with salt tolerance in treefrog (Albecker et al. 2021). Another strongly associated locus is a cluster of non‐coding SNPs located in a gene desert on Chr11 (−log_10_(*p*) = 40.6; Figure 3d and Figure S23), in the vicinity of one out of 116 copies of the *HARBI1* (Harbinger transposase derived 1) transposon in the European cisco genome. This signal was only identified in the contrast (d) freshwater lakes Vänern + Mälaren vs. population samples from the Bothnian Bay area (riverine and coastal) after excluding Kalix River.

We aimed to test if the signals of genetic differentiation between fresh and brackish water populations reflected recent strong selective sweeps, which may be revealed as a drastic reduction of nucleotide diversity in one type of populations. However, a comparison of the 11 genomic regions, which are the overlapping regions between the two ‘fresh vs. brackish water’ contrasts, did not indicate any clear trend in this direction (Table S3). This is also illustrated in the zoom‐in plots presented in Figures S19–S21.

A GO analysis revealed several statistically significant GO categories (corrected *p* value < 0.01) in the fresh vs. brackish water contrast (Files S8–S10). ‘Calcium ion binding’ was one of the significant enrichment terms in the ‘Kalix river vs. brackish’ contrast. The associated genes include *S100A10A* on chromosome 27 (Figure 3c and Figure S20), it's paralog *S100A11A* on chromosome 30 (Figure 3c and Figure S21), *CDH26* on chromosome 13 (Figure 3c and Figure S22), and *PCDH1* on chromosome 20 (Figure 3c and Figure S19).

## 4 Discussion

Here, we have shown that the European cisco is a species with moderately high genetic variability, with an overall nucleotide diversity estimated at 0.49%. We found a major subdivision between (i) populations from Lake Fegen and Stora Hålsjön and (ii) populations from the large freshwater lakes Vänern and Mälaren, and populations from the Bothnian Bay area (riverine and coastal) (Figure 1).

### 4.1 Genetic Population Structure

The current results are consistent with previous findings based on mtDNA and microsatellite variation documenting the existence of two genetically differentiated population groups within Sweden (Delling et al. 2014). The earlier studies revealed levels of nuclear genetic variation within and between populations that align well with the present genomic results based on a significantly larger number of loci. One of the population groups identified earlier (‘Group I’) appears to be restricted to a small area in south‐central Sweden, including Lakes Fegen and Stora Hålsjön, which lie above the highest historic shoreline of the Baltic basin. During the early stages of deglaciation, much of this region was covered by ice‐dammed lakes, collectively known as the South Swedish ice lake complex, which coexisted with the Baltic Ice Lake (Lundqvist and Nilsson 1959; Donner 2005). This suggests that Group I, with sympatric spring and autumn spawners, likely colonised the area relatively early after the ice retreated.

The second group (‘Group II’) appears to have subsequently colonised the remaining regions of the species' current distribution. This second ‘wave’ likely arrived during the freshwater Ancylus stage of the Baltic basin, while possible remnants of Group I ciscoes at lower altitudes in the Baltic Ice Lake may have been lost during the preceding marine Yoldia stage. Notably, no mtDNA haplotypes appear to be shared between these two groups; Group I haplotypes are closely related to those in least cisco ( *Coregonus sardinella* ) from North America, whereas Group II has mtDNA very similar to least ciscoes from the more proximate Siberia (Delling et al. 2014). These findings, which indicate a complex evolutionary history of Northern Hemisphere ciscoes, align with the frequent treatment of European cisco and least cisco as a single Holarctic species (e.g., Borovikova and Artamonova 2021).

### 4.2 Footprints of Selection

This study has revealed candidate loci underlying genetic adaptation to (i) spawning season and (ii) brackish water conditions. It illustrates how limited genetic drift, as indicated by average *F*_ST_ values ranging from 1.0% to 2.3%, provided optimal conditions for detecting loci involved in ecological adaptation, such as spawning in fresh versus brackish waters (Figure 3c,d and Figure S15). In contrast, when extensive genetic drift has occurred, such as between populations in Lakes Fegen and Stora Hålsjön and all other populations where the average *F*_ST_ has reached 16.3%, it is challenging to distinguish drift from selection when a small number of populations are analysed (Figure 3a). Similarly, the power to detect loci showing genetic differentiation between spring‐ and autumn‐spawners was limited by spring‐spawners being represented by a single sampled population (Lake Fegen). However, the contrast between this population and the autumn‐spawning populations from Lake Fegen and Stora Hålsjön resulted in the detection of more than 10 genomic regions showing genetic differentiation between spawning types, well exceeding the background differentiation due to genetic drift (Figure 3b and Figure S3). Several of these regions contain genes with documented roles in photoperiodic regulation of reproduction in vertebrates (Chen et al. 2020). This pathway responds to changes in day length, and the release of melatonin is considered a critical initial step in this pathway. Interestingly, a melatonin receptor gene (*MTNR1AA*) is located in a genomic region on chromosome 37 showing differentiation between spring‐ and autumn‐spawning ciscoes, approaching genome‐wide significance (Figure S17). Melatonin signalling in a specific region of the brain controlling photoperiodic regulation of reproduction results in the release of thyroid‐stimulating hormone (TSH) that activates the TSH receptor (TSHR) (Chen et al. 2020). TSHR signalling, in turn, leads to the release of luteinizing hormone (LH) and follicle‐stimulating hormone (FSH), which are of critical importance for reproduction. Thus, our population genomic screen revealed another strong positional candidate gene for controlling the timing of reproduction in the European cisco, *luteinizing hormone/choriogonadotropin receptor* (*LHCGR*) on chromosome 26 (Figures 3b and 4). There are two missense mutations (T62S and L94F) in *LHCGR* that show near‐complete fixation for different alleles in the spring‐ and autumn‐spawning populations in Lake Fegen (Figure 4c,d). The L94F mutation is particularly interesting because the L94 allele is the dominant variant in other salmonid fishes, all of which reproduce in autumn or winter (Figure 4d). This pattern suggests that L94F is a candidate causal mutation that may contribute to the difference in spawning time between spring‐and autumn‐spawning populations of European cisco. This hormone receptor has an essential role in gonadal maturation and reproduction (Ascoli et al. 2002).

A GO enrichment analysis revealed four more genes: two copies of *CPT1A*, *BHLHE40* and *TIMELESS*, associated with circadian rhythm biology (File S7). Of these, *BHLHE40* and *TIMELESS* are particularly interesting positional candidate genes for explaining genetic differentiation between spring‐ and autumn‐spawning populations in European cisco. BHLHE40 (alias Dec1) has been reported to be a regulator of the mammalian molecular clock by regulating the expression of Per1 (Honma et al. 2002). TIMELESS is a light‐sensitive clock protein first discovered in *Drosophila* that has a role in regulating photoperiodism (Abrieux et al. 2020). It is worth noticing that there is an indication that spring‐spawning cisco populations may spawn and occur in deeper water than autumn‐spawning forms (see Delling and Palm 2019), which implies that genetic differences in circadian genes may also reflect differences in light availability at different water depths. In fact, our GO analysis revealed a significant enrichment of genes in the visual perception category showing genetic differentiation between spring‐ and autumn‐spawning populations (File S7).

Our analysis of genetic adaptation to brackish water conditions included two distinct contrasts: Kalix River vs. Bothnian Bay and Lake Vänern/Mälaren vs. Bothnian Bay. The two groups of freshwater populations are geographically well separated, making recent gene flow between them unlikely (Figure 1). Our genetic analysis also shows that the Kalix River population is more closely related to the Bothnian Bay population than to the populations in Lake Mälaren and Vänern (Figure 2). Thus, the fact that almost 25% of all SNPs (288 out of 1199) detected in the contrast between Kalix River and Bothnian Bay (Figure 3c) were also outlier SNPs in the contrast between Lake Mälaren and Vänern vs. Bothnian Bay is notable, taking into account that the analysis involved about 20 million SNPs in total. We interpret the signals of selection detected in these two contrasts as primarily reflecting adaptation to spawning in the brackish water conditions in the Bothnian Bay (salinity 2‰–3‰), because the European cisco is a species that usually occurs as freshwater and anadromous forms that both spawn in freshwater. Thus, the Bothnian Bay population is unique by spawning in brackish water, whereas anadromous forms are exposed to brackish conditions as adults but are spawning in freshwater. However, the analysis of nucleotide diversities at loci showing genetic differentiation did not indicate the presence of recent hard selective sweeps causing reduced nucleotide diversities at these loci in brackish populations (Table S3).

In addition to the strong statistical support for genetic differentiation between populations adapted to freshwater or brackish conditions, the biological significance is supported by the fact that several of the genes detected in this study (*CDH26* and *TMTC3*) have previously been associated with salt tolerance in fish or other vertebrate species (Dennenmoser et al. 2017; Walsh et al. 2018; Albecker et al. 2021; Campo et al. 2022). Three of the genes (*CDH26*, *CA15b* and *PCDH1*) detected in the contrast between Kalix River and the Bothnian Sea were also identified in a previous RAD‐seq study using partially the same material (López et al. 2022), and the present study confirms these loci as some of the major outlier loci in this comparison.

Some of the most convincing loci showing genetic differentiation between freshwater populations and the Bothnian Bay population concern loci that have not previously been associated with salt tolerance directly, but have an important role during early development in zebrafish. This is interesting because our interpretation is that the differentiation we observe is related to the ability to spawn in freshwater or brackish water. One example is the two paralogs *SNX27A* and *SNX27B* on chromosomes 27 and 30, respectively, that both are replicated in both contrasts (Figure 3c,d; and Figures S20, S21 and S24). *SNX27* genes encode nexin proteins important for endosome trafficking and signalling; the single *SNX27* gene in zebrafish is critical for normal neuronal growth and brain development (Yong et al. 2021). A second particularly interesting example concerns *CA15b* on chromosome 17, also associated with both contrasts between freshwater populations and the Bothnian Bay samples (Figure 3c,d). *CA15b* is one of many genes in fish encoding carbonic anhydrases that are essential for the regulation of acid‐salt balance. *CA15b* has evolved by duplication from the *CA4* gene (Aspatwar et al. 2022; Dichiera et al. 2023) and is expressed in primordial germ cells during early development in zebrafish (Wang et al. 2013). *CA15b* shows copy number expansion in salmonids and occurs as a tandem cluster of 4–5 genes in these species (Figure 5e). These duplicated genes show concerted evolution since the different copies are more similar to other copies in the same species or closely related species than to copies in more distantly related salmonids (Figure S25).

For some of the signals of genetic differentiation between freshwater and Bothnian Bay populations, allele frequencies in Lake Mälaren were more similar to those of brackish water populations than to other freshwater populations. This was particularly pronounced for the *TMTC3* locus on chromosome 8 (Figure 6c). *TMTC3*, the most striking signal in contrast c (Kalix River vs. all other population samples from the Bothnian Bay area), has previously been associated with salt adaptation in birds and fish (Dennenmoser et al. 2017; Walsh et al. 2018). This brackish‐like pattern for Lake Mälaren is also noted for a cluster of genes on chromosome 27 (*CHRNA7A*, *SNX27A*, *S100A10A* and *TRIM46*), which are shared between the two contrasts between ‘freshwater’ and ‘Bothnian Bay’ populations. The average frequency of the ‘brackish’ haplotype for this region was as follows: Kalix River = 0.50, Bothnian Bay populations = 0.90, Lake Mälaren = 0.61 in contrast to other freshwater lakes = 0.15 (Figure S20). It is worth noting that while these signals suggest a pattern similar to brackish water populations, most other signals indicate that Lake Mälaren follows the same pattern as other freshwater lakes (Figure 5; and Figures S18 and S22). Lake Mälaren, situated just west of Stockholm, outflows into the Baltic Sea to the east (Figure 1). This geographic connection to the Baltic Sea suggests potential gene flow between Lake Mälaren and Baltic Sea populations. To further investigate this hypothesis, additional analysis incorporating more coastal populations is needed. It may further be noted that Lake Mälaren constituted a bay of the Baltic Sea until relatively recently (c. 1000 years before present), suggesting a potential common origin with ciscoes presently found in the brackish waters of the Stockholm Archipelago.

This study has resulted in a number of strong signals of genetic differentiation related to variation in seasonal reproduction and adaptation to fresh vs. brackish water conditions. The results also highlight the need for broader screening of genetic variation across multiple populations. It would be particularly rewarding to include additional population samples of spring‐spawning European cisco since the current study only included a single population of this type. Unfortunately, with the exception of Fegen, all Swedish spring‐spawning populations have gone extinct, while a few such populations still exist in Germany and Finland (e.g., Delling and Palm 2019).

### 4.3 Implications for Fishery Management

The roe from European cisco spawning in the Bothnian Bay is considered a delicacy and sustains an important commercial fishery (Bergenius et al. 2013; Lehtonen et al. 2023). Sustainable exploitation of any fish stock requires accurate knowledge about the population structure. Our results indicated low genetic differentiation among the population samples from the Bothnian Bay, but a genetically differentiated population is spawning in the freshwater Kalix River, which is one of the few unexploited rivers in Sweden with regards to hydropower. The Kalix River cisco samples were collected some distance upstream, relatively isolated compared to the other river samples caught closer to the river mouth. A further indication of genetic heterogeneity was evident based on two outlier individuals from the Lule River, which together with the Kalix River population suggests that lower reaches of the large rivers in the Bothnian Bay may support freshwater spawning cisco populations which are genetically differentiated from coastal spawning populations, but with some migration of individuals between spawning locations. This genetic heterogeneity needs to be further explored in follow‐up studies and, if confirmed, should be considered in future fishery management plans.

## Author Contributions

L.A. conceived the study. K.L.‐T. contributed to the conceptualisation of the project. J.H. and M.‐B.M. performed nucleic acid extractions for reference genome sequencing. I.B., R.‐A.O., M.P., L.S. and H.L. were responsible for genome assembly and annotation. O.V.P. have designed and supervised reference genome sequence generation. E.E. advised, managed and supervised the HiC sequencing experiment. A.C. generated the Tn5 libraries for whole genome resequencing. Q.D. was responsible for the population genomics analysis. J.G. and M.E.P. contributed to the population genomics analysis. B.D., A.V., S.P. and M.B.N. contributed with sample collections and with knowledge of European cisco biology. Q.D. and L.A. wrote the paper with input from other authors. All authors approved the paper before submission.

## Conflicts of Interest

The authors declare no conflicts of interest.

## Supporting information


**File S1:** List of SNPs showing strong genetic differentiation between super‐pools of Southern Swedish Lakes Fegen and Stora Hålsjön (*n* = 90) vs. all other European cisco populations (*n* = 246).
**File S2:** List of SNPs showing strong genetic differentiation between spring spawners (*n* = 30) and autumn spawners in European cisco (*n* = 60).
**File S3:** List of SNPs showing strong genetic differentiation between super‐pools of Kalix River (*n* = 21) and all other population samples from the Bothnian Bay area (riverine and coastal) (*n* = 157).
**File S4:** List of SNPs showing strong genetic differentiation between super‐pools of freshwater lakes Vänern + Mälaren (*n* = 66) and population samples from the Bothnian Bay area (riverine and coastal) after excluding Kalix River (*n* = 157).
**File S5:** List of overlapping SNPs showing strong genetic differentiation between contrast_c: freshwater Kalix River vs. all other population samples from the Bothnian Bay area (riverine and coastal) and contrast_d: freshwater lakes Vänern + Mälaren vs. population samples from the Bothnian Bay area (riverine and coastal) after excluding Kalix River in European cisco.
**File S6:** List of overlapping genes showing strong genetic differentiation between contrast_c: freshwater Kalix River vs. all other population samples from the Bothnian Bay area (riverine and coastal) and contrast_d: freshwater lakes Vänern + Mälaren vs. population samples from the Bothnian Bay area (riverine and coastal) after excluding Kalix River in European cisco.
**File S7:** GO enrichment analysis of genes showing high differentiation in contrast_b spring spawners vs. autumn spawners in Lakes Fegen and Stora Hålsjön.
**File S8:**. GO enrichment analysis of genes showing high differentiation in contrast_c: freshwater Kalix River vs. all other population samples from the Bothnian Bay area (riverine and coastal).
**File S9:**. GO enrichment analysis of genes showing high differentiation in contrast_d: freshwater lakes Vänern + Mälaren vs. population samples from the Bothnian Bay area (riverine and coastal) after excluding Kalix River.
**File S10:**. GO enrichment analysis of overlapping genes showing high differentiation between contrast_c: freshwater Kalix River vs. all other population samples from the Bothnian Bay area (riverine and coastal) and contrast_d: freshwater lakes Vänern + Mälaren vs. population samples from the Bothnian Bay area (riverine and coastal) after excluding Kalix River.

**Figure S1:** Histogram of −log_10_(*p*) value in European cisco populations. Arrows indicate proportions of SNPs within the respective −log_10_(*p*) brackets, with the number of SNPs shown above each arrow. (a) Lakes Fegen + Stora Hålsjön vs. all other population samples. (b) Spring spawners vs. autumn spawners in Lakes Fegen and Stora Hålsjön. (c) Freshwater Kalix River vs. all other population samples from the Bothnian Bay area (riverine and coastal). (d) freshwater lakes Vänern + Mälaren vs. population samples from the Bothnian Bay area (riverine and coastal) after excluding Kalix River. (e) Bonferroni and −log_10_(*p*) threshold in each contrast. The blue proportions in the histogram (a, b) represent the threshold used in Figure 3. The 0.01% threshold in panel (a) includes a lot of noise signals, so a 0.001% threshold, was used instead.
**Figure S2:** Genome‐wide screen of genetic differentiation for each chromosome in the contrast (a) Lakes Fegen + Stora Hålsjön vs. all other population samples. The horizontal dashed black lines represent the top 0.01% of SNPs, and the solid black lines represent the top 0.001% of SNPs.
**Figure S3:** Genome‐wide screen of genetic differentiation for each chromosome in the contrast (b) Spring spawners vs. autumn spawners in Lakes Fegen and Stora Hålsjön. The horizontal dashed black lines represent the top 0.01% of SNPs. A total of 1079 highly differentiated SNPs were detected, corresponding to 75 independent signals, located around 79 genes. Putative inversions on Chr8, 9 and 19 are highlighted with orange boxes.
**Figure S4:** Genome‐wide screen of genetic differentiation for each chromosome in the contrast (c) freshwater Kalix River vs. all other population samples from the Bothnian Bay area (riverine and coastal). The horizontal dashed black lines represent the top 0.01% of SNPs. A total of 1199 highly differentiated SNPs were detected, corresponding to 75 independent signals, located around 79 genes. Putative inversions on Chr20 and 26 are highlighted with orange boxes.
**Figure S5:** Genome‐wide screen of genetic differentiation for each chromosome in the contrast (d) freshwater lakes Vänern + Mälaren vs. population samples from the Bothnian Bay area (riverine and coastal) after excluding Kalix River. The horizontal dashed black lines represent the top 0.01% of SNPs. A total of 1237 highly differentiated SNPs were detected, corresponding to 90 independent signals, located around 95 genes. Putative inversions on Chr20 and 28 are highlighted with orange boxes.
**Figure S6:** Hi‐C contact map of the curated scaffolds of European cisco (*Coregonus albula*), visualised in HiGlass.
**Figure S7:** Manhattan plot showing read depth across the genome. Average read depth is calculated in 50 kb non‐overlapping windows and plotted for each chromosome. Chromosomes 034, 36 and 37 exhibit markedly higher coverage compared to the rest of the genome.
**Figure S8:** Circos plot comparing the structure of European cisco (*Coregonus albula*) (left) and *Coregonus sp. ‘Balchen’* (right, https://doi.org/10.1111/1755‐0998.13187) chromosomes—created with JupiterPlot (https://github.com/JustinChu/JupiterPlot, and the following arguments minBundleSize = 400,000, gScaff = 1, maxGap = 400,000, ng = 0, labels = both). Dashed lines within the chromosomes represent gaps and dashed lines linking chromosomes represent the borders of contiguous aligned regions (alignment blocks). Most alignments suggest a good match between both assemblies (32 chromosomes show the same structure with exactly one corresponding ‘Balchen’ chromosome and 8 chromosomes show structural differences).
**Figure S9:** Circos plot comparing the structure of European cisco (*Coregonus albula*) (left) and the *Coregonus clupeaformis* (right, https://doi.org/10.1111/mec.16468) chromosomes—created with JupiterPlot (https://github.com/JustinChu/JupiterPlot, and the following arguments: minBundleSize = 400,000, gScaff = 1, maxGap = 400,000, ng = 0, labels = both). Dashed lines within the chromosomes represent gaps and dashed lines linking chromosomes represent the borders of contiguous aligned regions (alignment blocks). Most alignments suggest a good match between both assemblies (35 chromosomes show the same structure with exactly one corresponding *C. clupeaformis* chromosome. Chromosomes RL31, RL34, RL36 and RL37 have very sparse alignments and show the biggest differences).
**Figure S10:** Circos plot comparing the structure of European cisco (*Coregonus albula*) (left) and *Coregonus artedi* (right, https://doi.org/10.1038/s42003‐024‐06503‐z) chromosomes—created with JupiterPlot (https://github.com/JustinChu/JupiterPlot, and the following arguments: minBundleSize = 400,000, gScaff = 1, maxGap = 400,000, ng = 0, labels = both). Dashed lines within the chromosomes represent gaps and dashed lines linking chromosomes represent the borders of contiguous aligned regions (alignment blocks). Most alignments suggest a good match between both assemblies (32 chromosomes show the same structure with exactly one corresponding *C. artedi* chromosome. Chromosomes RL31, RL34, RL36 and RL37 have very sparse alignments and show the biggest differences).
**Figure S11:** Gene size distribution in the European cisco (*Coregonus albula*) genome annotation. (a) Gene size distribution of the 51,040 genes present in the annotation of European cisco. (b) Size distribution of genes with lengths between 0 and 20,000 base pairs, based on the same genome annotation data.
**Figure S12:** Read mapping in European cisco. (a) Summary of sequencing data analysis for 336 samples. (b) Relationship between mean read depthand median insert size. Each point in the scatter plot represents a data sample, and the red regression line indicates a positive correlation between the two variables. (c) Median insert size across different populations. (d) Mean read depthacross different populations. In panels c and d, error bars represent the interquartile range (IQR) of the data.
**Figure S13:** Histogram of sequencing depth across all 336 samples. The red dashed line indicates the average depth. Blue lines represent the depth filter thresholds. Depth filter thresholds were set to average depth ±50% (from 0.5× to 1.5× the mean depth). Note that all sites with depth > 2000 were grouped into a single bin.
**Figure S14:** Admixture analysis of European cisco populations. (a) Based on all populations. (b) Using only samples from Lakes Fegen and Stora Hålsjön. (c) All other samples after excluding those from Lakes Fegen and Stora Hålsjön. (d) Using only Bothnian Bay samples. (e) Using all Bothnian bay samples, after excluding 21 samples from Kalix River that stand out in Figure 2d and two samples from Lule River (upper right corner in Figure 2d).
**Figure S15:** Average *F*_ST_ and dxy between populations with different ecological adaptations in European cisco. The average *d*_xy_ and *F*_ST_ values are summarised in the table. Contrast_a: Lakes Fegen + Stora Hålsjön vs. all other population samples. Contrast_b: Spring spawners vs. autumn spawners in Lakes Fegen and Stora Hålsjön. Contrast_c: freshwater Kalix River vs. all other population samples from the Bothnian Bay area (riverine and coastal). Contrast_d: freshwater lakes Vänern + Mälaren vs. population samples from the Bothnian Bay area (riverine and coastal) after excluding Kalix River.
**Figure S16:** Examples of highly differentiated genes in the contrast spring spawners vs. autumn spawners in Lakes Fegen and Stora Hålsjön in European cisco—*SLC24A2* and *NANS*. (a) Genome‐wide diversity statistics F_ST_, π and −log10(p) across the *SLC24A2* and *NANS* locus on Chr37. F_ST_ and −log10(p) represent single SNP data while π is calculated for 10‐kb windows. The boxes with red borders indicate the genes surrounding the signals. The highlighted gene is the one closest to the most significant SNPs among the genes. ‘UN’ denotes an unannotated gene. (b) Heatmaps of allele frequencies across the *SLC24A2* and *NANS*.
**Figure S17:** Examples of highly differentiated genes in the contrast spring spawners vs. autumn spawners in Lakes Fegen and Stora Hålsjön in European cisco—*MTNR1AA*. (a) Genome‐wide diversity statistics F_ST_, π and −log10(p) across the *MTNR1AA* locus on Chr37. F_ST_ and −log10(p) represent single SNP data while π is calculated for 10‐kb windows. The boxes with red borders indicate the genes surrounding the signals. The highlighted gene is the one closest to the most significant SNPs among the genes. ‘UN’ denotes an unannotated gene. (b) Heatmap of allele frequencies across the *MTNR1AA* region.
**Figure S18:** Examples of highly differentiated genes in the contrast spring spawners vs. autumn spawners in Lakes Fegen and Stora Hålsjön in European cisco—*TIMELESS*. (a) Genome‐wide diversity statistics F_ST_, π and −log10(p) across the *TIMELESS* locus on Chr6. F_ST_ and −log10(p) represent single SNP data while π is calculated for 10‐kb windows. The boxes with red borders indicate the genes surrounding the signals. The highlighted gene is the one closest to the most significant SNPs among the genes. ‘UN’ denotes an unannotated gene. (b) Heatmap of allele frequencies across the *TIMELESS* region.
**Figure S19:** Example of shared signal of genetic differentiation in the vicinity of the putative inversion 6 in contrast_c: freshwater Kalix River vs. all other population samples from the Bothnian Bay area (riverine and coastal) and contrast_d: freshwater lakes Vänern + Mälaren vs. population samples from the Bothnian Bay area (riverine and coastal) after excluding Kalix River in European cisco. (a, b) Genome‐wide diversity statistics F_ST_, π and −log10(p) across the putative inversion 6 locus on Chr20. F_ST_ and −log10(p) represent single SNP data while π is calculated for 10‐kb windows. The boxes with red borders indicate the genes surrounding the signals. The highlighted gene *PCDH1* appears as a shared signal of genetic differentiation in both contrasts. ‘UN’ denotes an unannotated gene. (a) Freshwater Kalix River vs. all other population samples from the Bothnian Bay area (riverine and coastal). (b) Freshwater lakes Vänern + Mälaren vs. population samples from the Bothnian Bay area (riverine and coastal) after excluding Kalix River. (c) Heatmap of allele frequencies across the putative inversion 6 locus.
**Figure S20:** Example of shared signal of genetic differentiation in the vicinity of the *CHRNA7A*, *SNX27A*, *S100A10A* and *TRIM46* loci in contrast_c: freshwater Kalix River vs. all other population samples from the Bothnian Bay area (riverine and coastal) and contrast_d: freshwater lakes Vänern + Mälaren vs. population samples from the Bothnian Bay area (riverine and coastal) after excluding Kalix River in European cisco. (a, b) Genome‐wide diversity statistics F_ST_, π and −log10(p) across the *CHRNA7A*, *SNX27A*, *S100A10A* and *TRIM46* loci on Chr27. F_ST_ and −log10(p) represent single SNP data while π is calculated for 10‐kb windows. The boxes with red borders indicate the genes surrounding the signals. ‘UN’ denotes unannotated genes. The highlighted gene is the ones closest to the most significant SNPs. (a) Freshwater Kalix River vs. all other population samples from the Bothnian Bay area (riverine and coastal). (b) Freshwater lakes Vänern + Mälaren vs. population samples from the Bothnian Bay area (riverine and coastal) after excluding Kalix River. (c) Heatmap of allele frequencies across the shared signal of genetic differentiation on Chr27.
**Figure S21:** Example of shared signal of genetic differentiation in the vicinity of the *SNX27B* and *CHRNA7B* loci in contrast_c: freshwater Kalix River vs. all other population samples from the Bothnian Bay area (riverine and coastal) and contrast_d: freshwater lakes Vänern + Mälaren vs. population samples from the Bothnian Bay area (riverine and coastal) after excluding Kalix River in European cisco. (a, b) Genome‐wide diversity statistics F_ST_, π and −log10(p) across the *SNX27B* and *CHRNA7B* loci on Chr30. F_ST_ and −log10(p) represent single SNP data while π is calculated for 10‐kb windows. The boxes with red borders indicate the genes surrounding the signals. ‘UN’ denotes unannotated genes. The highlighted gene is the ones closest to the most significant SNPs. (a) Freshwater Kalix River vs. all other population samples from the Bothnian Bay area (riverine and coastal). (b) Freshwater lakes Vänern + Mälaren vs. population samples from the Bothnian Bay area (riverine and coastal) after excluding Kalix River. (c) Heatmap of allele frequencies across the shared signal of genetic differentiation on Chr30.
**Figure S22:** Examples of highly differentiated genes between contrast_c: freshwater Kalix River vs. all other population samples from the Bothnian Bay area (riverine and coastal) in European cisco—*CDH26*. (a) Genome‐wide diversity statistics F_ST_, π and −log10(p) across the *CDH26* locus on Chr13. F_ST_ and −log10(p) represent single SNP data while π is calculated for 10‐kb windows. The boxes with red borders indicate the genes surrounding the signals. The highlighted gene is the one closest to the most significant SNPs among the genes. (b) Heatmap of allele frequencies across the *CDH26* region.
**Figure S23:** Examples of highly differentiated genes in the contrast_d: freshwater lakes Vänern + Mälaren vs. population samples from the Bothnian Bay area (riverine and coastal) after excluding Kalix River—downstream of HARBI1. (a, b) Genome‐wide diversity statistics F_ST_, π and −log10(p) across the *HARBI1* downstream locus on Chr11. F_ST_ and −log10(p) represent single SNP data while π is calculated for 10‐kb windows. The boxes with red borders indicate the genes surrounding the signals. The highlighted gene is the one closest to the most significant SNPs among the genes. ‘UN’ denotes an unannotated gene. (a) Freshwater Kalix River vs. all other population samples from the Bothnian Bay area (riverine and coastal). (b) Freshwater lakes Vänern + Mälaren vs. population samples from the Bothnian Bay area (riverine and coastal) after excluding Kalix River. (c) Heatmap of allele frequencies across the *HARBI1* downstream locus.
**Figure S24:** Phylogenetic tree of *SNX27*, *CHRNA7* and *S100A* nucleotide sequences. The tree was constructed using the Neighbour‐Joining (NJ) method. Sequences from European cisco are highlighted in blue boxes.
**Figure S25:** Phylogenetic tree based on *CA15* nucleotide sequences in salmonids. The tree was constructed using the Neighbour‐Joining (NJ) method. The tree includes the *CA15a*, *CA15b* and *CA15c* sequences from zebrafish, highlighted in orange. *CA15b* copies from European cisco are highlighted in blue boxes.*CA4* in Lopez et al. (2022) was blasted against the reference genome of *Coregonus clupeaformis* and is highlighted in green in our tree, where it also clusters with zebrafish *CA15b*.

**Table S1:** Population samples of European cisco included in the study and their average genome‐wide nucleotide diversity (*π*).
**Table S2:** Summary of gene annotations for the *Coregonus albula* reference genome, fCorAlb1.
**Table S3:** Locus‐specific nucleotide diversity (*π*) of overlapping signals of genetic differentiation between contrast (c): freshwater Kalix River vs. all other population samples from the Bothnian Bay area (riverine and coastal) and (d): freshwater lakes Vänern + Mälaren vs. population samples from the Bothnian Bay area (riverine and coastal) after excluding Kalix River.

## Data Availability

Genome assembly for *Coregonus albula* , fCorAlb1 (accession ID: CAWUPP020000000.2) is available at https://www.ebi.ac.uk/ena/browser/view/CAWUPP020000000. PacBio long read data and HiC sequencing data from this study are available through https://www.ebi.ac.uk/ena/browser/view/PRJEB62720. Low coverage, short read data for the population study are available through https://www.ncbi.nlm.nih.gov/bioproject/PRJNA1278710. Code availability: All code used to analyse sequence data are available at https://github.com/LeifAnderssonLab/Cisco_PopSeq.
