# Supplementary material for: Genetic Adaptation to Brackish Water and Spawning Season in European Cisco
Source: Mol Ecol. 2025 Sep 3;34(20):e70094. doi: 10.1111/mec.70094 (PMC12530279; doi:10.1111/mec.70094)
Supplement: Supplementary file 2 — Figure S1: Histogram of −log10(p) value in European cisco populations. Arrows indicate proportions of SNPs within the respective −log10(p) brackets, with the number of SNPs shown above each arrow. (a) Lakes Fegen + Stora Hålsjön vs. all other population samples. (b) Spring spawners vs. autumn spawners in Lakes Fegen and Stora Hålsjön. (c) Freshwater Kalix River vs. all other population samples from the Bothnian Bay area (riverine and coastal). (d) freshwater lakes Vänern + Mälaren vs. population samples from the Bothnian Bay area (riverine and coastal) after excluding Kalix River. (e) Bonferroni and −log10(p) threshold in each contrast. The blue proportions in the histogram (a, b) represent the threshold used in Figure 3. The 0.01% threshold in panel (a) includes a lot of noise signals, so a 0.001% threshold, was used instead. Figure S2: Genome‐wide screen of genetic differentiation for each chromosome in the contrast (a) Lakes Fegen + Stora Hålsjön vs. all other population samples. The horizontal dashed black lines represent the top 0.01% of SNPs, and the solid black lines represent the top 0.001% of SNPs. Figure S3: Genome‐wide screen of genetic differentiation for each chromosome in the contrast (b) Spring spawners vs. autumn spawners in Lakes Fegen and Stora Hålsjön. The horizontal dashed black lines represent the top 0.01% of SNPs. A total of 1079 highly differentiated SNPs were detected, corresponding to 75 independent signals, located around 79 genes. Putative inversions on Chr8, 9 and 19 are highlighted with orange boxes. Figure S4: Genome‐wide screen of genetic differentiation for each chromosome in the contrast (c) freshwater Kalix River vs. all other population samples from the Bothnian Bay area (riverine and coastal). The horizontal dashed black lines represent the top 0.01% of SNPs. A total of 1199 highly differentiated SNPs were detected, corresponding to 75 independent signals, located around 79 genes. Putative inversions on Chr20 and 26 are highlight [file MEC-34-e70094-s001.pdf]

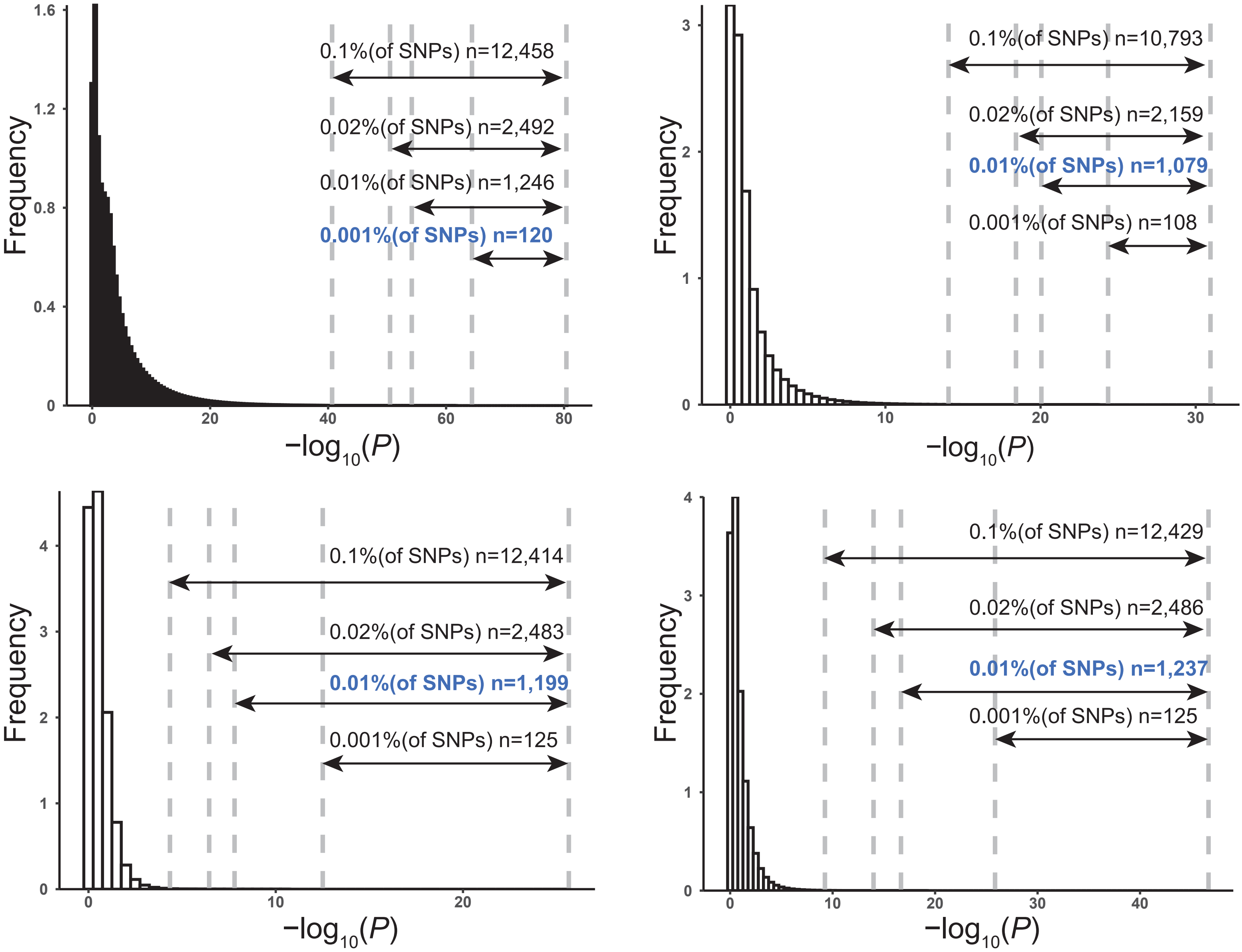

(e)

Contrast	Bonferroni threshold (α=0.05)	$-\log_{10}(P)$ threshold 0.01%	$-\log_{10}(P)$ threshold 0.001%
a	8.4	54.3	64.5
b	8.3	20.1	24.4
c	8.4	7.8	12.5
d	8.4	16.7	25.8

Figure S1. Histogram of $-\log_{10}(P)$ value in European cisco populations. Arrows indicate proportions of SNPs within the respective $-\log_{10}(P)$ brackets, with the number of SNPs shown above each arrow. (a) Lakes Fegen + Stora Hålsjön vs. all other population samples. (b) Spring spawners vs. autumn spawners in Lakes Fegen and Stora Hålsjön. (c) Freshwater Kalix River vs. all other population samples from the Bothnian Bay area (riverine and coastal). (d) freshwater lakes Vänern + Mälaren vs. population samples from the Bothnian Bay area (riverine and coastal) after excluding Kalix River. (e) Bonferroni and $-\log_{10}(P)$ threshold in each contrast. The blue proportions in the histogram (a-b) represent the threshold used in Figure 3. The 0.01% threshold in panel (a) includes a lot of noise signals, so a 0.001% threshold, was used instead.

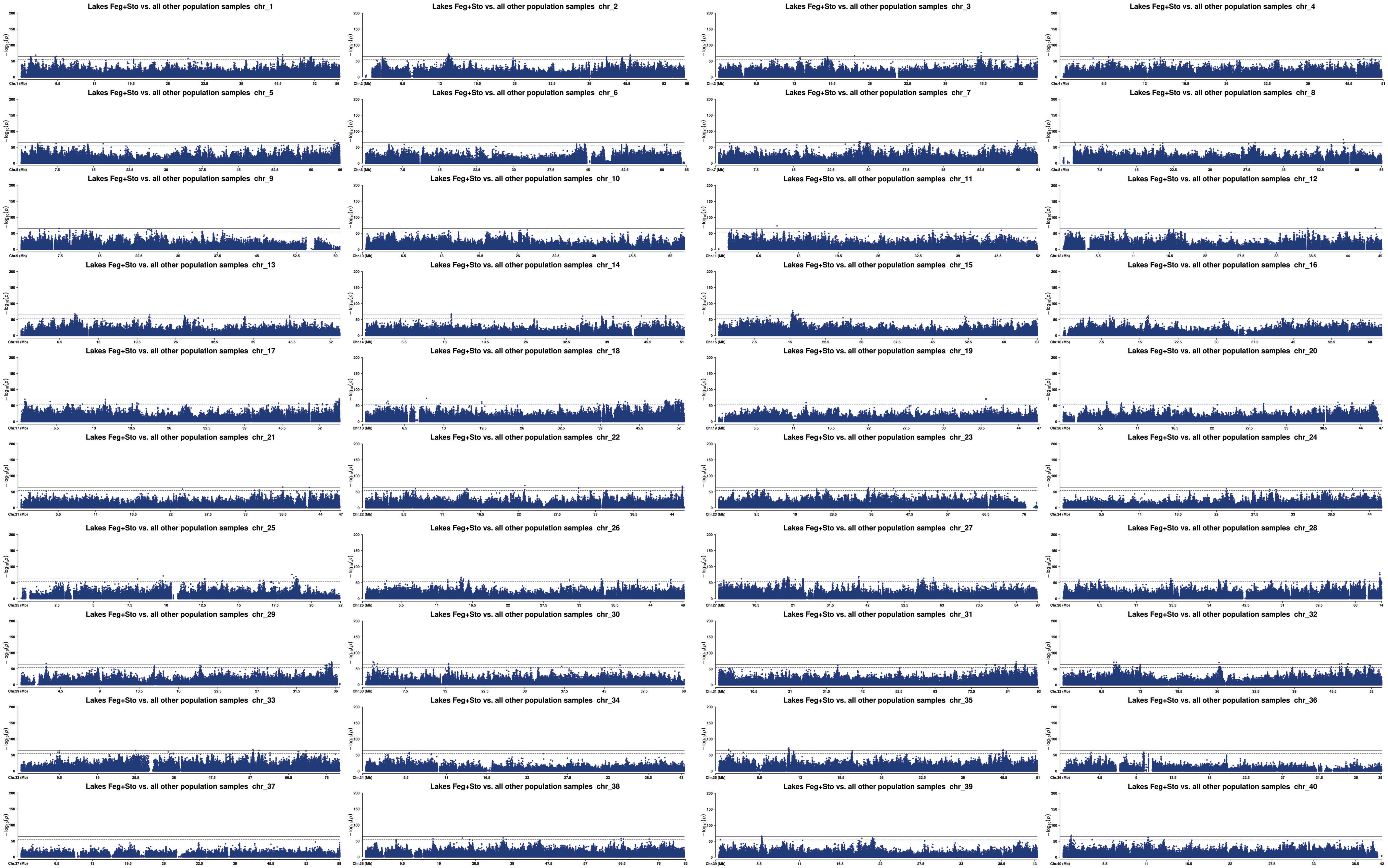

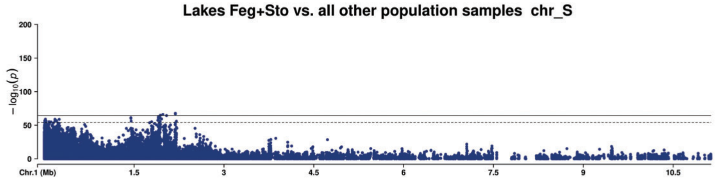

Figure S2. Genome-wide screen of genetic differentiation for each chromosome in the contrast (a) Lakes Fegen + Stora Hålsjön vs. all other population samples. The horizontal dashed black lines represent the top 0.01% of SNPs, and the solid black lines represent the top 0.001% of SNPs.

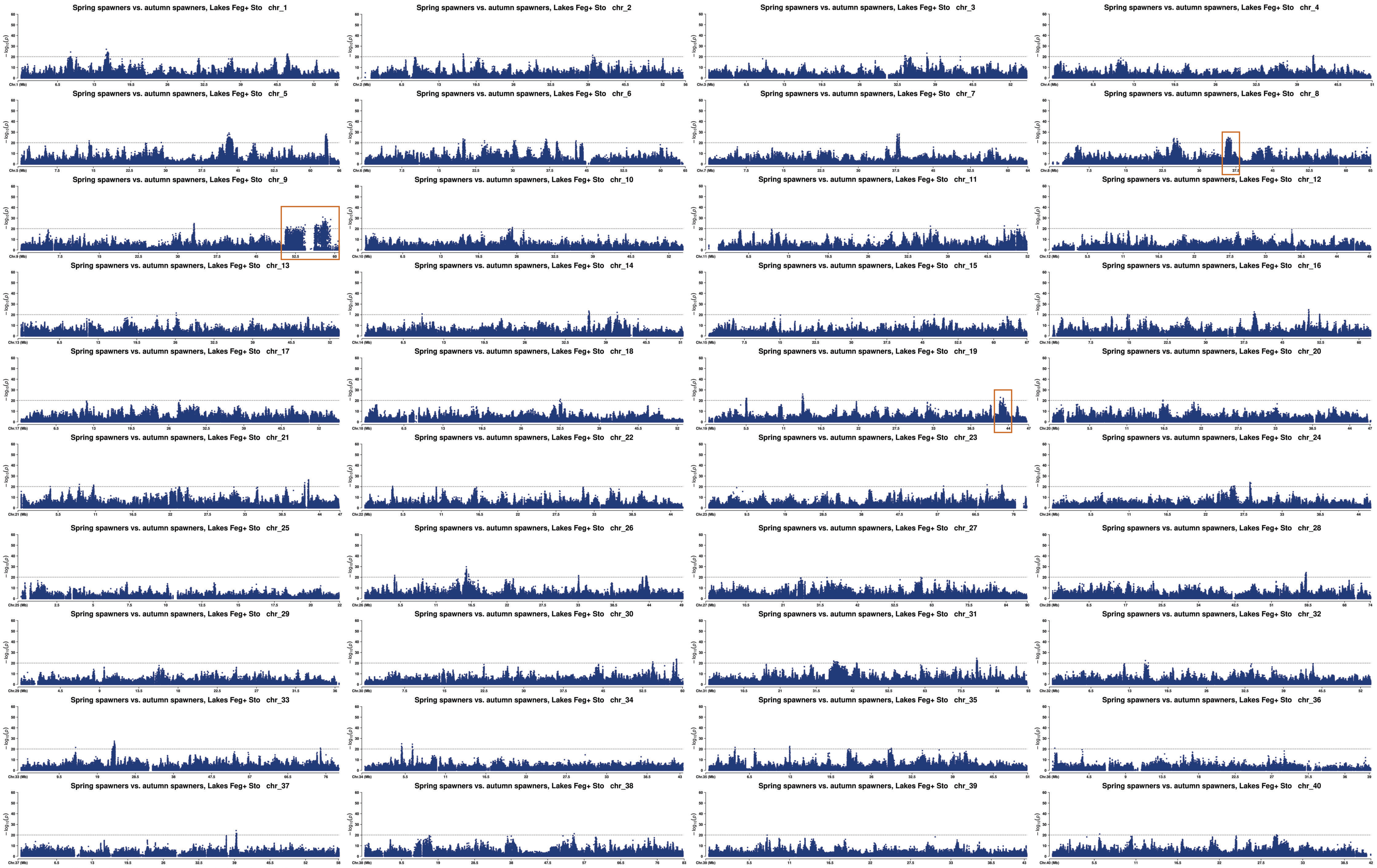

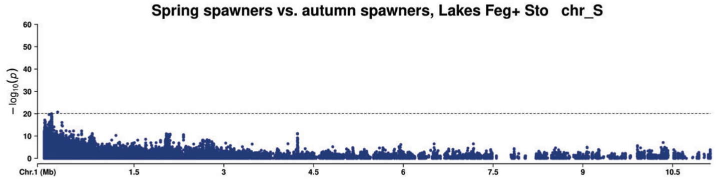

Figure S3. Genome-wide screen of genetic differentiation for each chromosome in the contrast (b) Spring spawners vs. autumn spawners in Lakes Fegen and Stora Hålsjön. The horizontal dashed black lines represent the top 0.01% of SNPs. A total of 1,079 highly differentiated SNPs were detected, corresponding to 75 independent signals, located around 79 genes. Putative inversions on Chr8, 9, and 19 are highlighted with orange boxes.

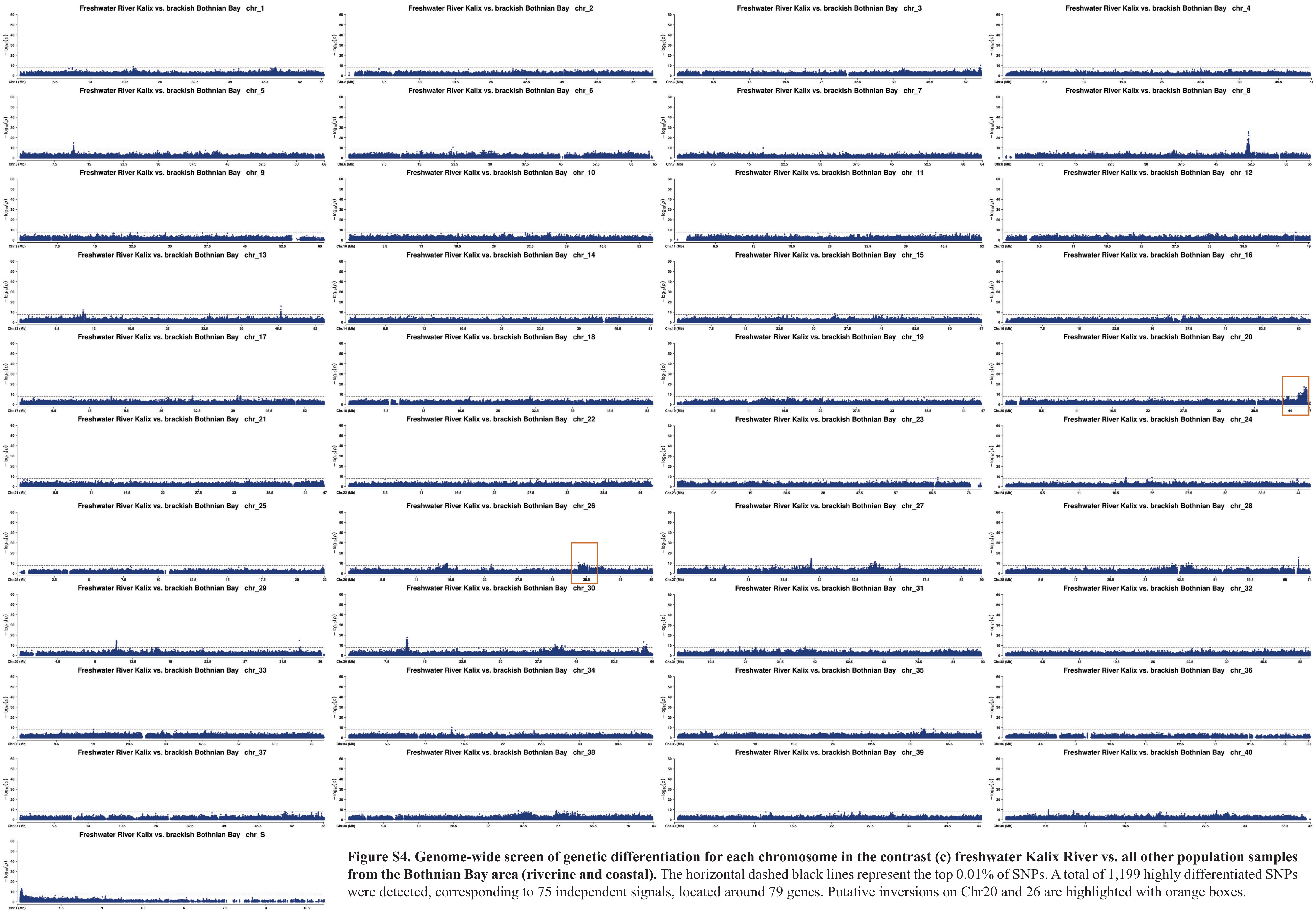

Figure S4. Genome-wide screen of genetic differentiation for each chromosome in the contrast (c) freshwater Kalix River vs. all other population samples from the Bothnian Bay area (riverine and coastal). The horizontal dashed black lines represent the top 0.01% of SNPs. A total of 1,199 highly differentiated SNPs were detected, corresponding to 75 independent signals, located around 79 genes. Putative inversions on Chr20 and 26 are highlighted with orange boxes.

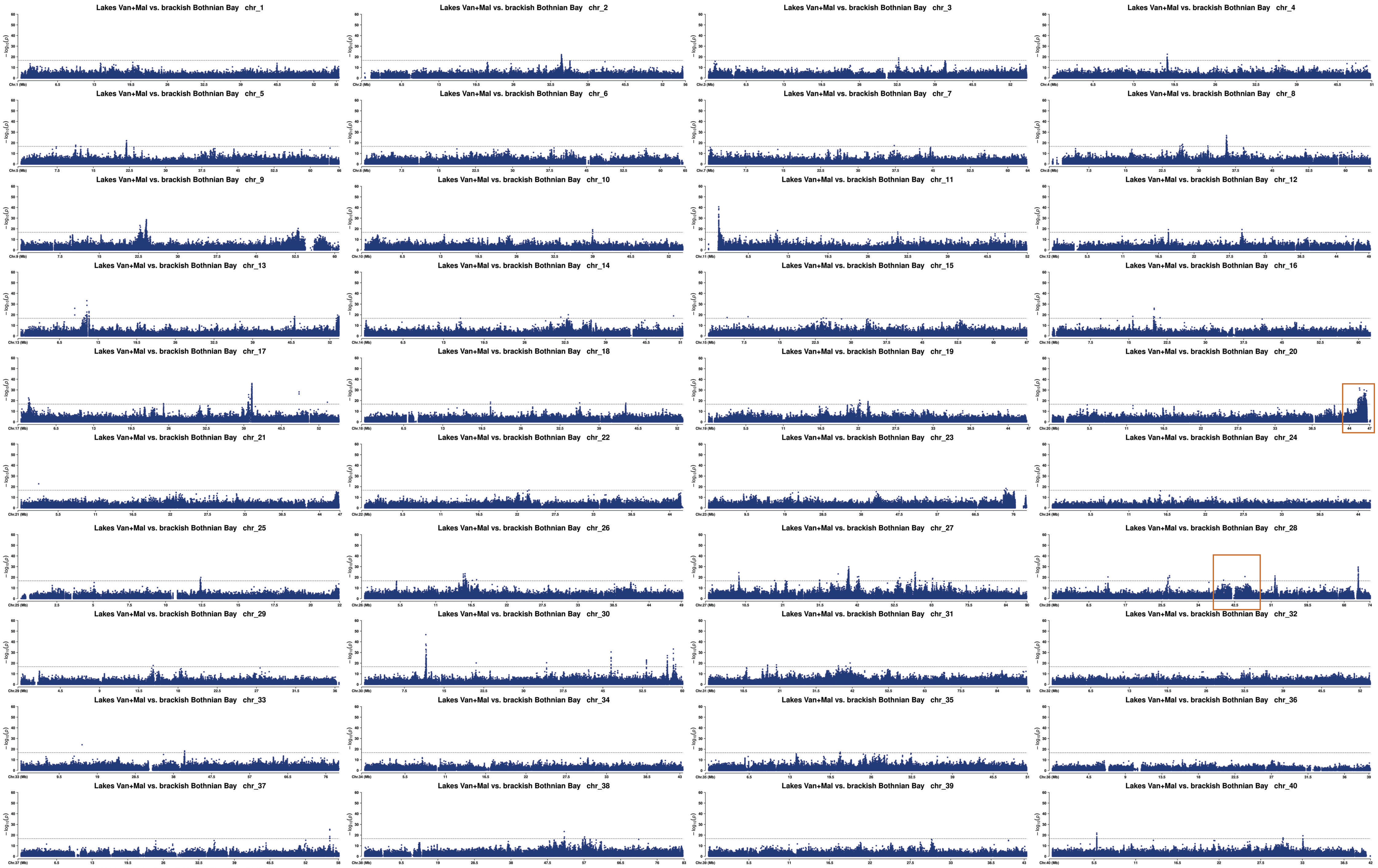

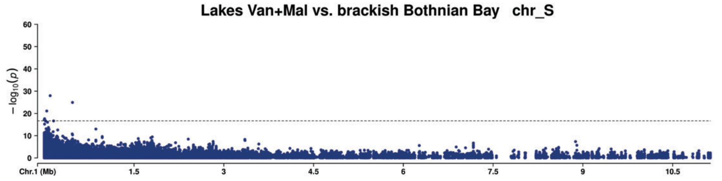

Figure S5. Genome-wide screen of genetic differentiation for each chromosome in the contrast (d) freshwater lakes Vänern + Mälaren vs. population samples from the Bothnian Bay area (riverine and coastal) after excluding Kalix River. The horizontal dashed black lines represent the top 0.01% of SNPs. A total of 1,237 highly differentiated SNPs were detected, corresponding to 90 independent signals, located around 95 genes. Putative inversions on Chr20 and 28 are highlighted with orange boxes.

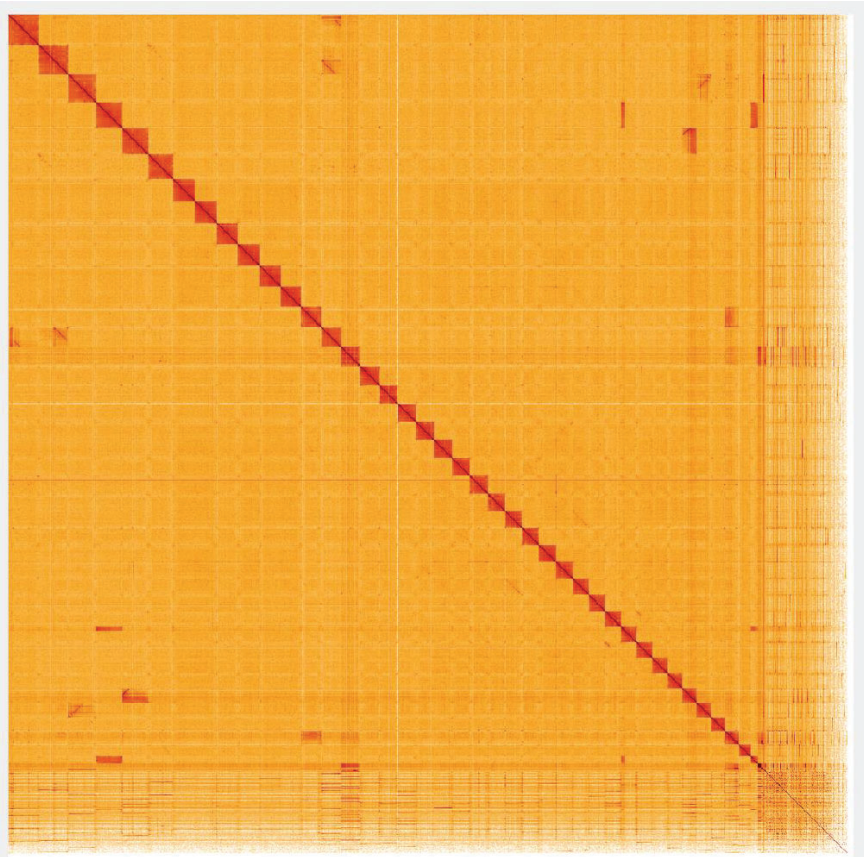

Figure S6. Hi-C contact map of the curated scaffolds of European cisco (*Coregenus albula)*, visualized in HiGlass.

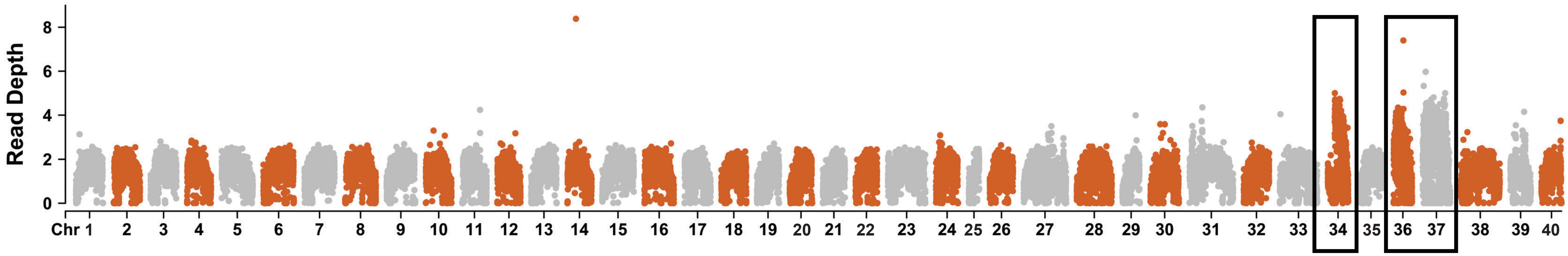

Figure S7. Manhattan plot showing read depth across the genome. Average read depth is calculated in 50 kb non-overlapping windows and plotted for each chromosome.Chromosomes 34, 36, and 37 exhibit markedly higher coverage compared to the rest of the genome.

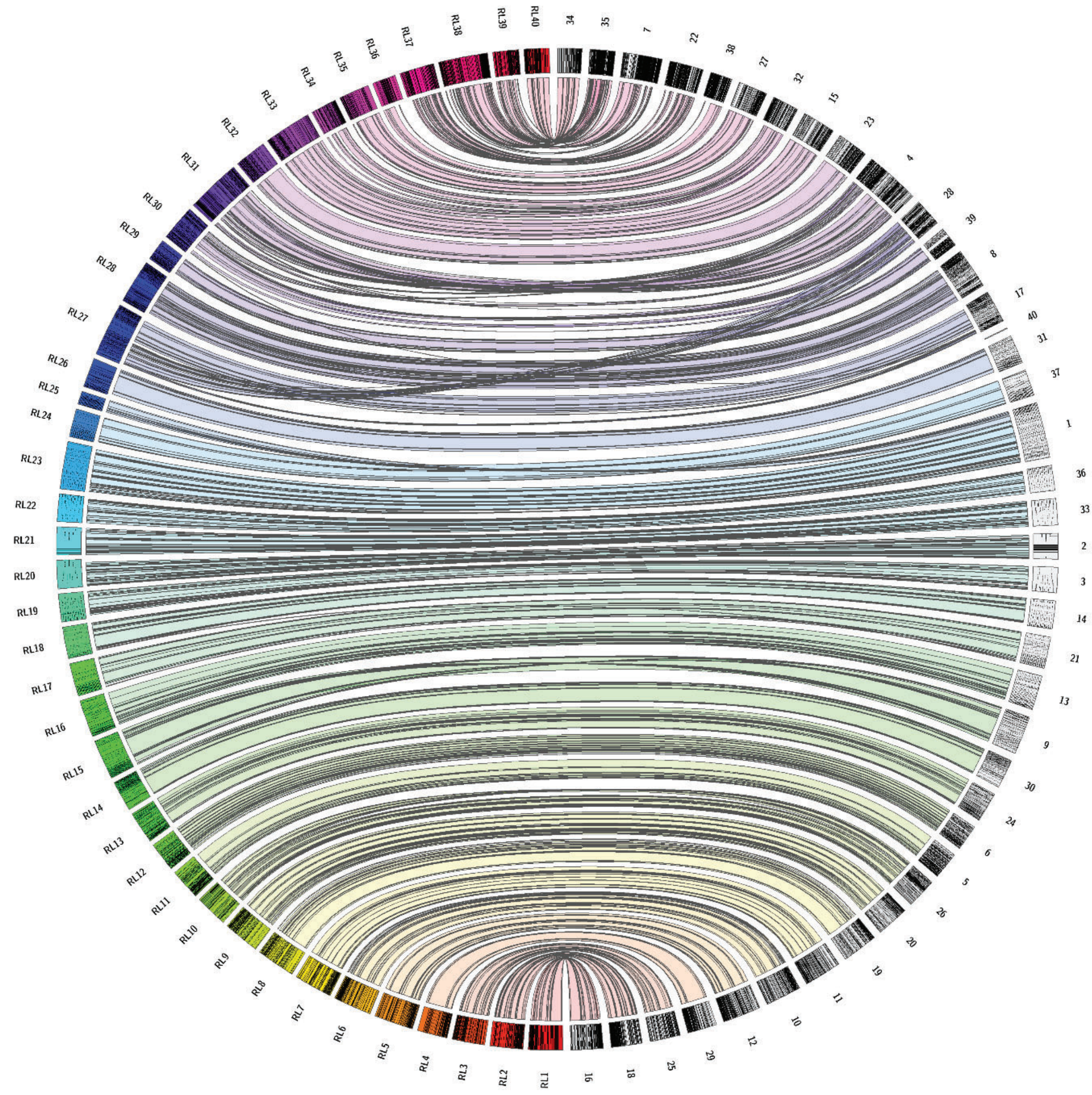

Figure S8. Circos plot comparing the structure of European cisco (*Coregonus albula*) (left) and *Coregonus* sp. "*Balchen*" (right, https://doi.org/10.1111/1755-0998.13187) chromosomes – created with JupiterPlot (https://github.com/JustinChu/JupiterPlot, and the following arguments minBundleSize=400000, gScaff=1, maxGap=400000, ng=0, labels=both). Dashed lines within the chromosomes represent gaps and dashed lines linking chromosomes represent the borders of contiguous aligned regions (alignment blocks). Most alignments suggest a good match between both assemblies (32 chromosomes show the same structure with exactly one corresponding ´*Balchen*´ chromosome and 8 chromosomes show structural differences)

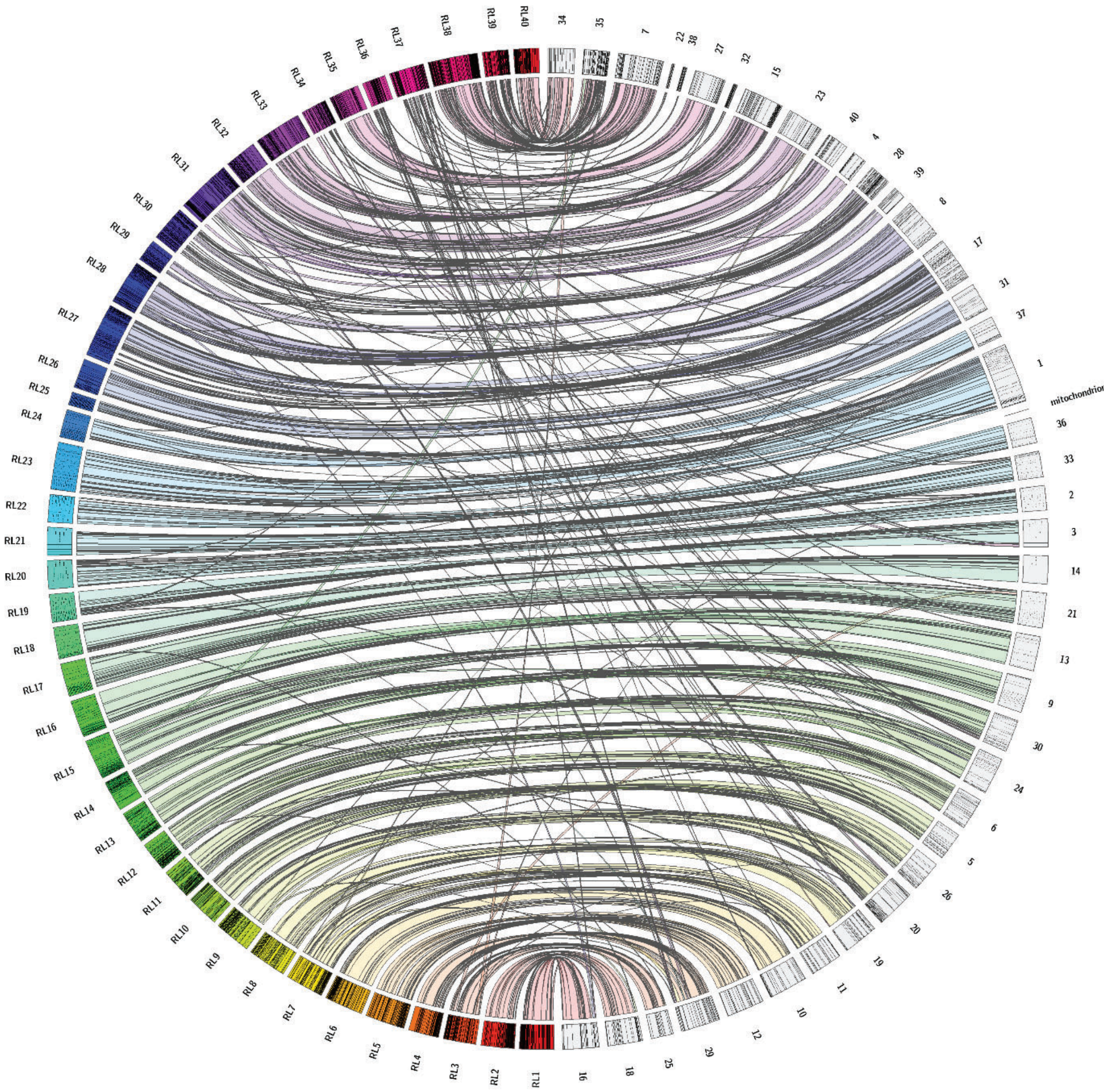

Figure S9. Circos plot comparing the structure of European cisco (*Coregonus albula*) (left) and the *Coregonus clupeaformis* (right, https://doi.org/10.1111/mec.16468) chromosomes – created with JupiterPlot (https://github.com/JustinChu/JupiterPlot, and the following arguments: minBundleSize=400000, gScaff=1, maxGap=400000, ng=0, labels=both). Dashed lines within the chromosomes represent gaps and dashed lines linking chromosomes represent the borders of contiguous aligned regions (alignment blocks). Most alignments suggest a good match between both assemblies (35 chromosomes show the same structure with exactly one corresponding *C. clupeaformis* chromosome. Chromosomes RL31, RL34, RL36 and RL37 have very sparse alignments and show the biggest differences)

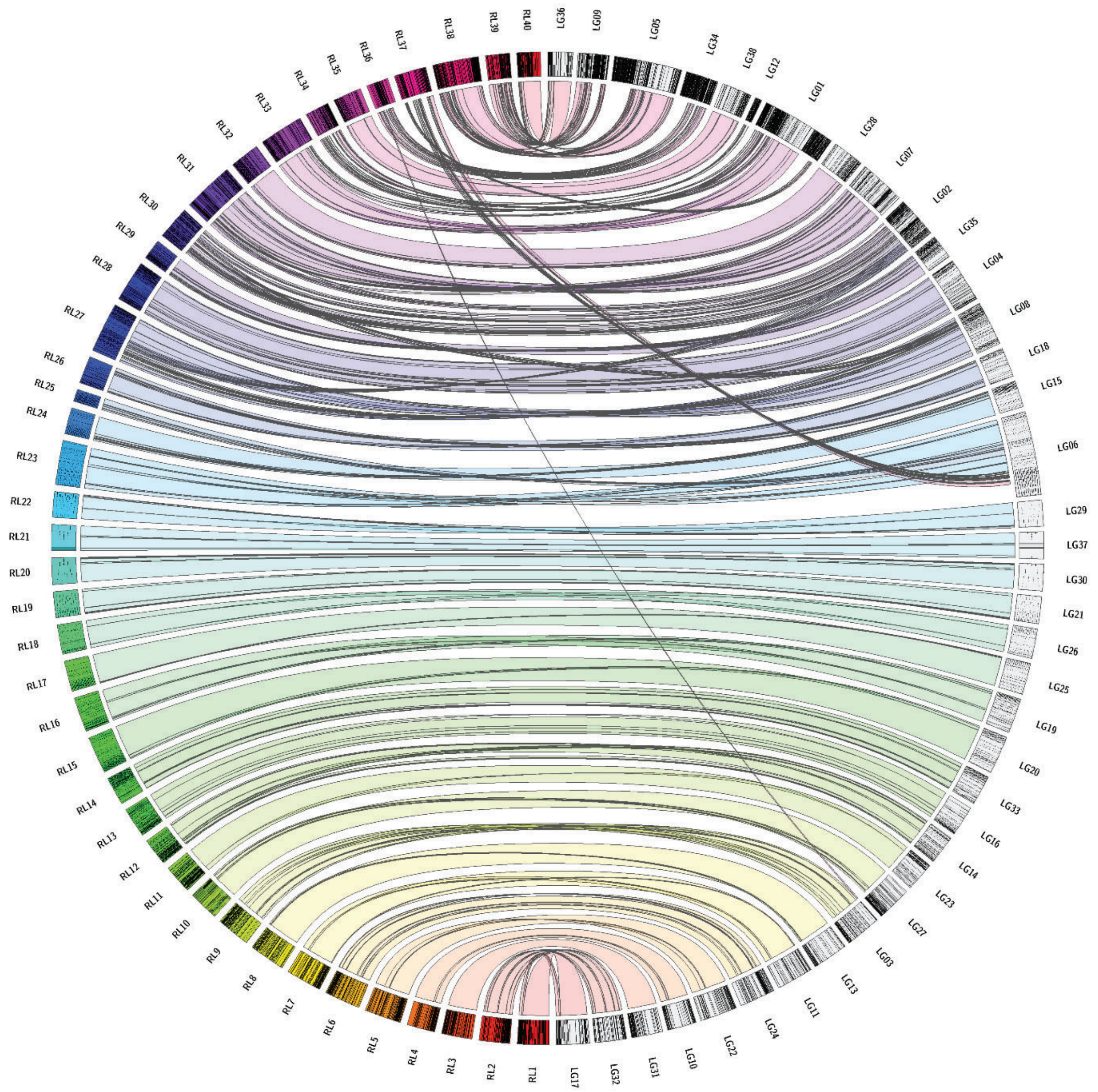

Figure S10. Circos plot comparing the structure of European cisco (*Coregonus albula*) (left) and *Coregonus artedi* (right, https://doi.org/10.1038/s42003-024-06503-z) chromosomes– created with JupiterPlot (https://github.com/JustinChu/JupiterPlot, and the following arguments: minBundleSize=400000, gScaff=1, maxGap=400000, ng=0, labels=both). Dashed lines within the chromosomes represent gaps and dashed lines linking chromosomes represent the borders of contiguous aligned regions (alignment blocks). Most alignments suggest a good match between both assemblies (32 chromosomes show the same structure with exactly one corresponding *C. artedi* chromosome. Chromosomes RL31, RL34, RL36 and RL37 have very sparse alignments and show the biggest differences).

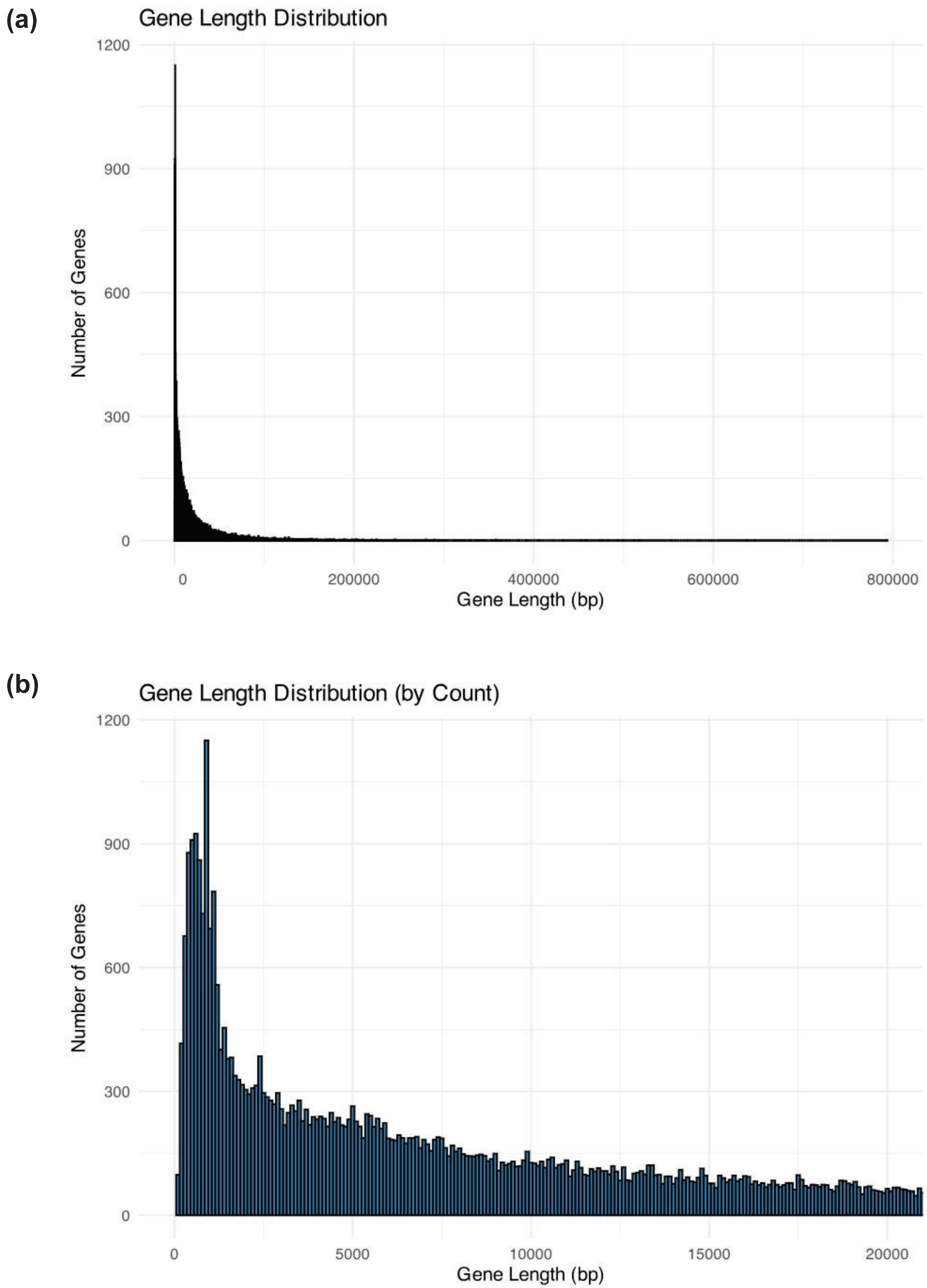

Figure S11. Gene size distribution in the European cisco (*Coregonus albula*) genome annotation. (a) Gene size distribution of the 51040 genes present in the annotation of European cisco. (b) Size distribution of genes with lengths between 0 and 20,000 base pairs, based on the same genome annotation data.

(a)

Summary	
Number of samples	336
Total number of mapped reads	7,056,477,028
Average genome read depth	1.08x
Average GC-content	45.69%
Average mapping quality	31.15
Average insert size	281.94 bp

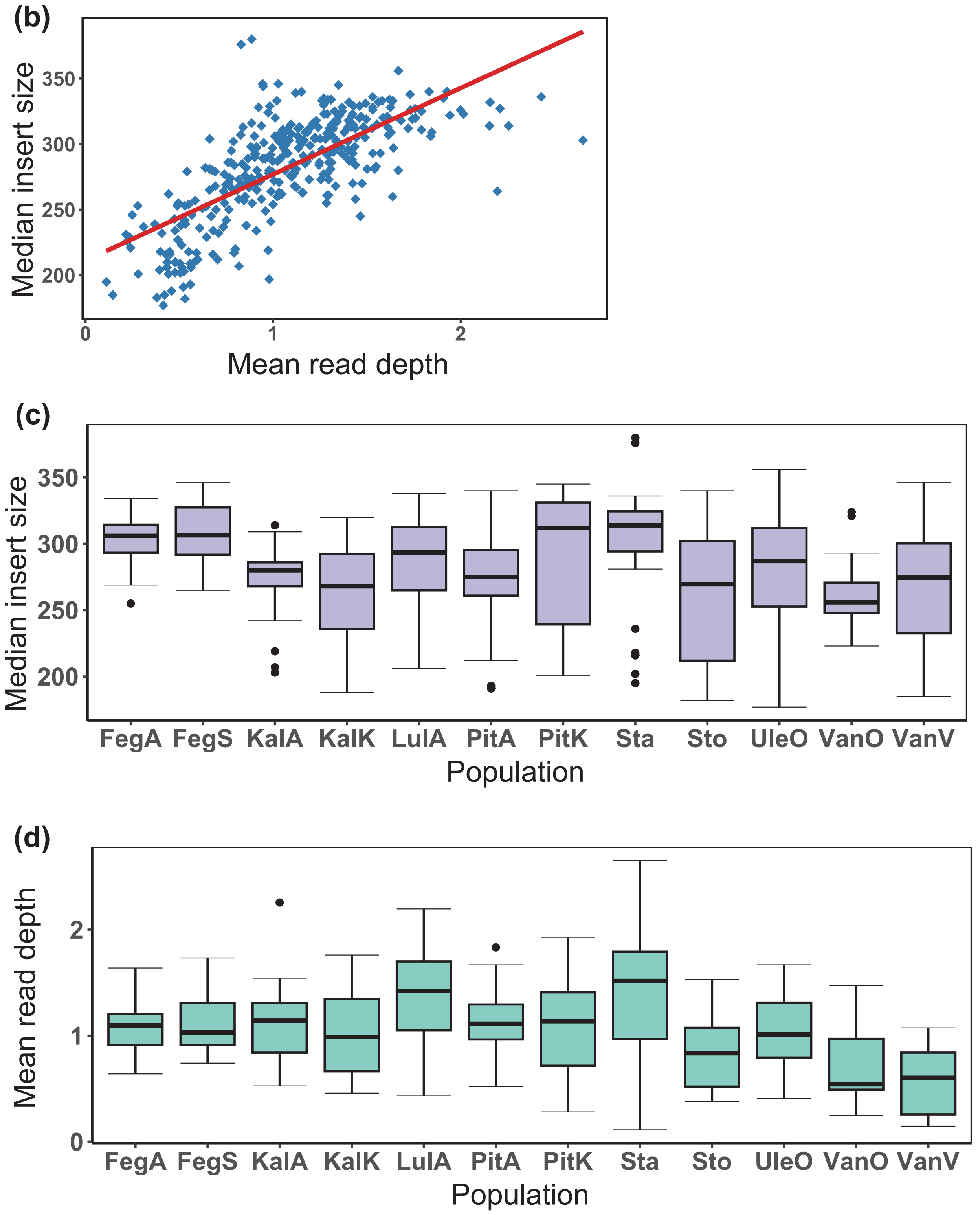

Figure S12. Read mapping in European cisco. (a) Summary of sequencing data analysis for 336 samples. (b) Relationship between mean read depth and median insert size. Each point in the scatter plot represents a data sample, and the red regression line indicates a positive correlation between the two variables. (c) Median insert size across different populations. (d) Mean read depth across different populations. In panels c and d, error bars represent the interquartile range (IQR) of the data.

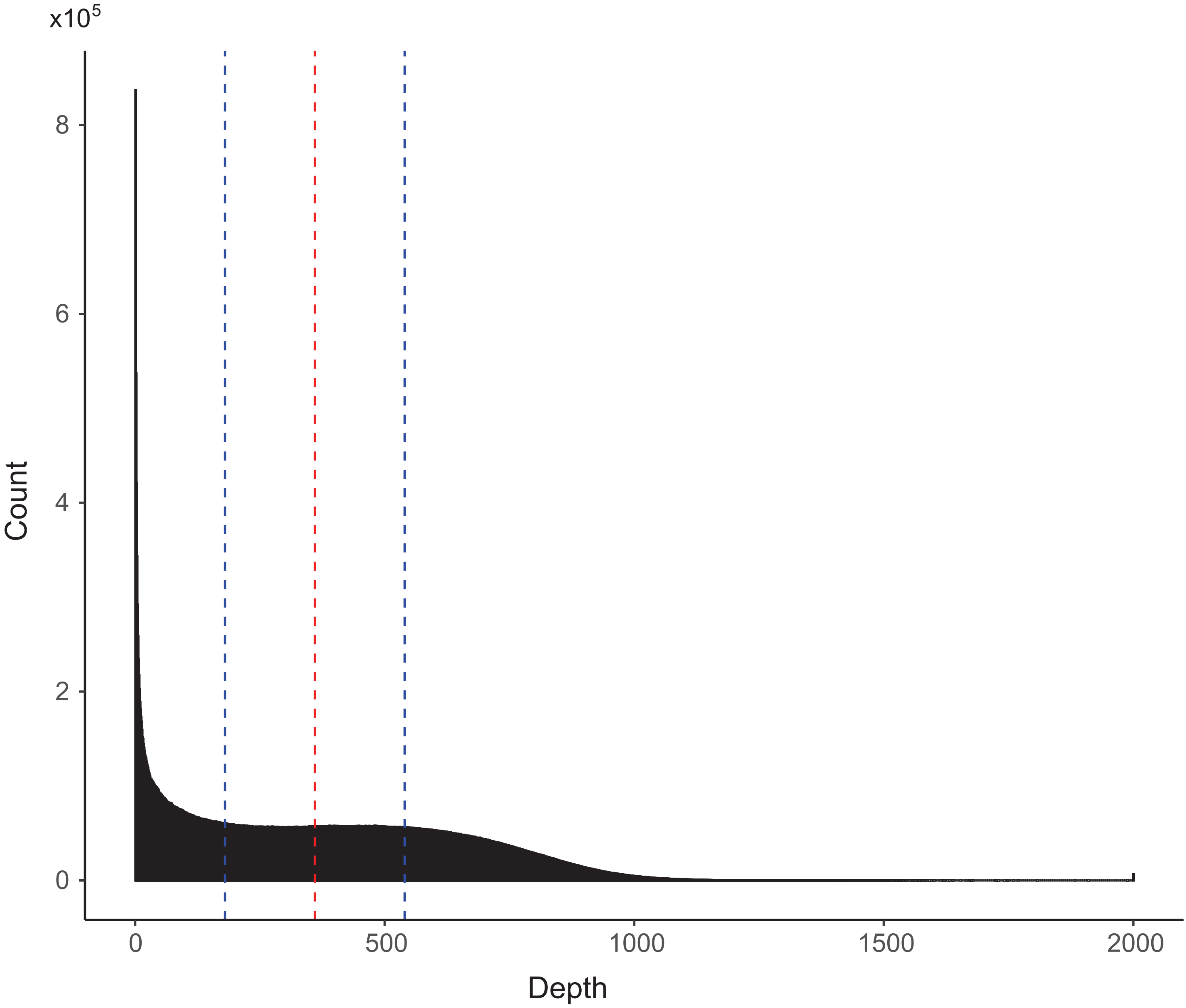

Figure S13. Histogram of sequencing depth across all 336 samples. The red dashed line indicates the average depth. Blue lines represent the depth filter thresholds. Depth filter thresholds were set to average depth ± 50% (from 0.5× to 1.5× the mean depth). Note that all sites with depth > 2000 were grouped into a single bin.

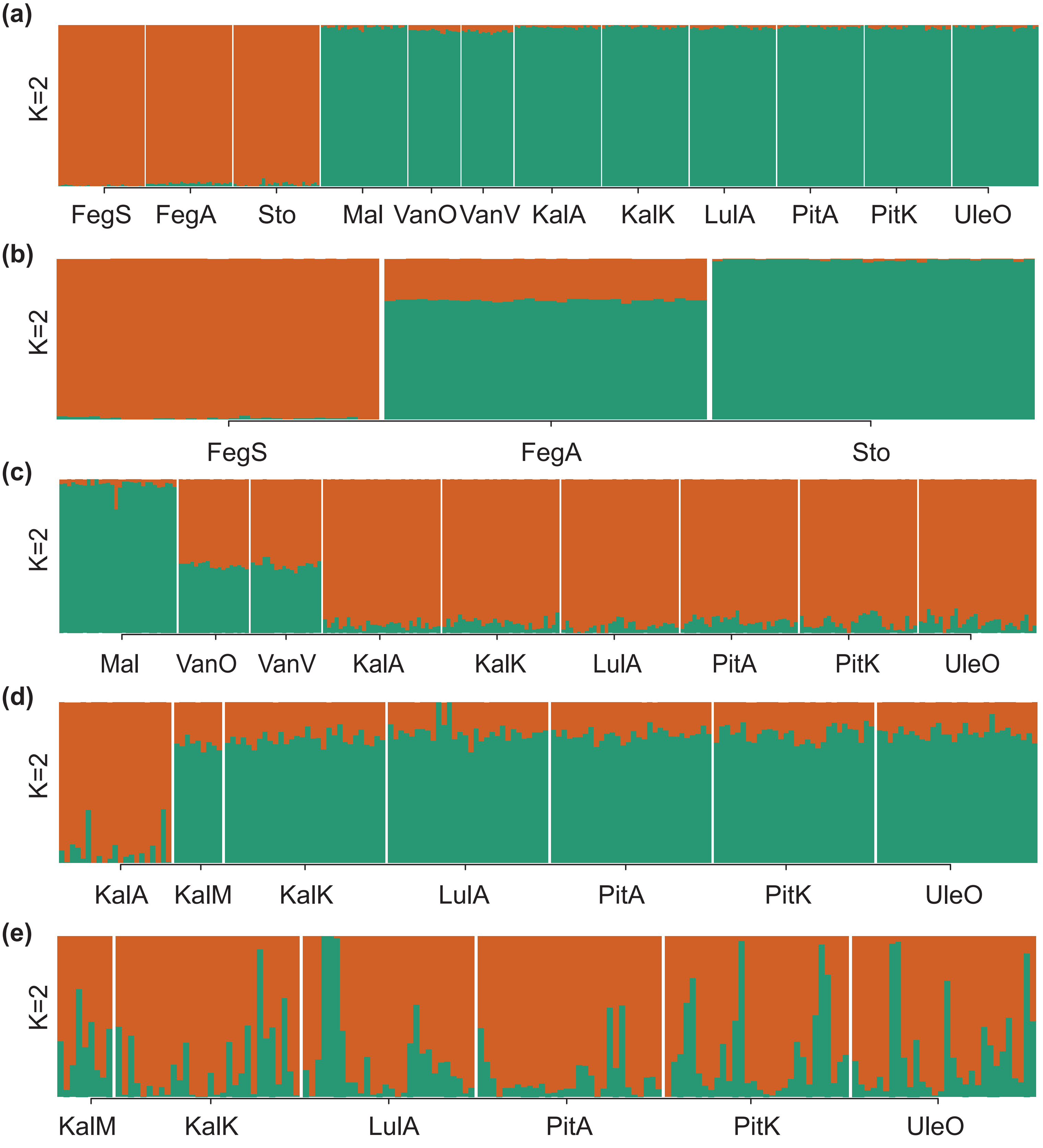

Figure S14. Admixture analysis of European cisco populations. (a) Based on all populations. (b) Using only samples from Lakes Fegen and Stora Hålsjön. (c) All other samples after excluding those from Lakes Fegen and Stora Hålsjön. (d) Using only Bothnian Bay samples. (e) Using all Bothnian bay samples, after excluding 21 samples from Kalix River that stand out in Figure 2d and two samples from Lule River (upper right corner in Figure 2d).

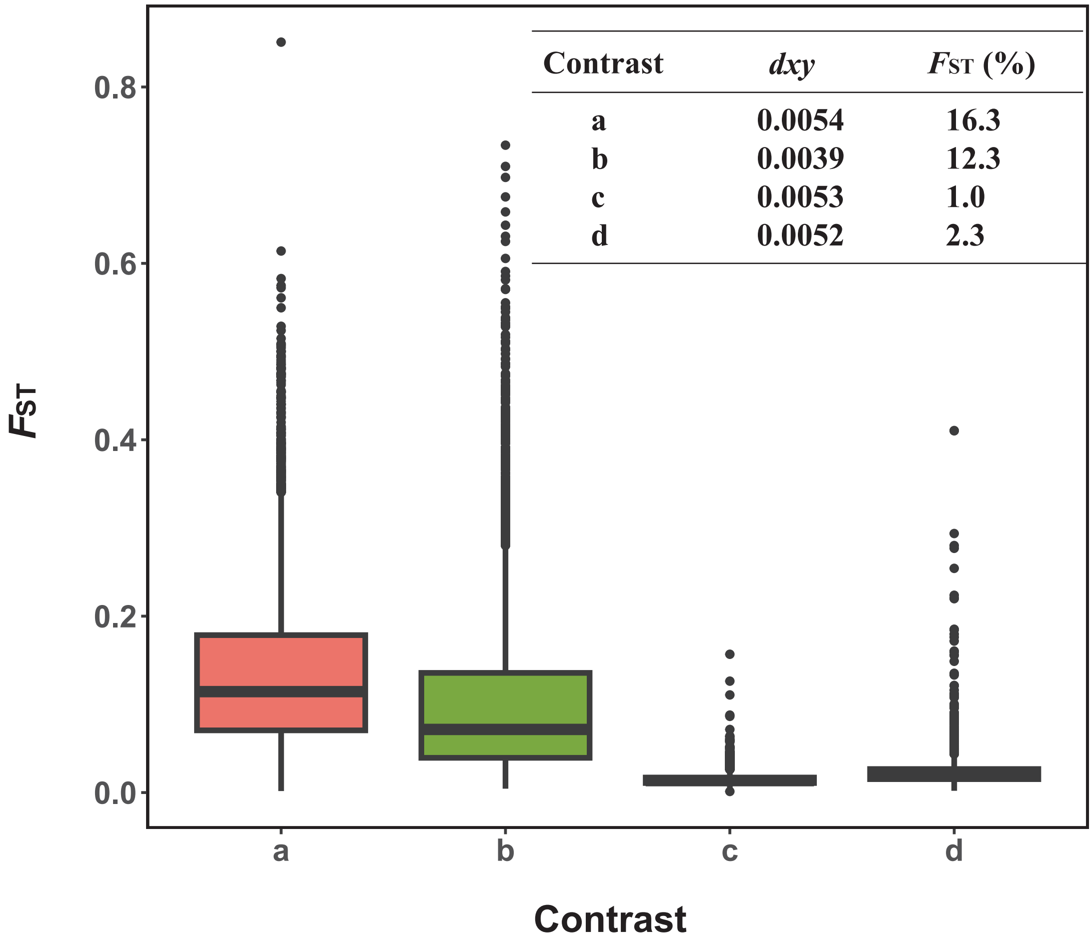

Figure S15. Average F_{ST} and *dxy* between populations with different ecological adaptations in European cisco. The average *dxy* and F_{ST} values are summarized in the table. Contrast_a: Lakes Fegen + Stora Hålsjön vs. all other population samples. Contrast_b: Spring spawners vs. autumn spawners in Lakes Fegen and Stora Hålsjön. Contrast_c: freshwater Kalix River vs. all other population samples from the Bothnian Bay area (riverine and coastal). Contrast_d: freshwater lakes Vänern + Mälaren vs. population samples from the Bothnian Bay area (riverine and coastal) after excluding Kalix River.

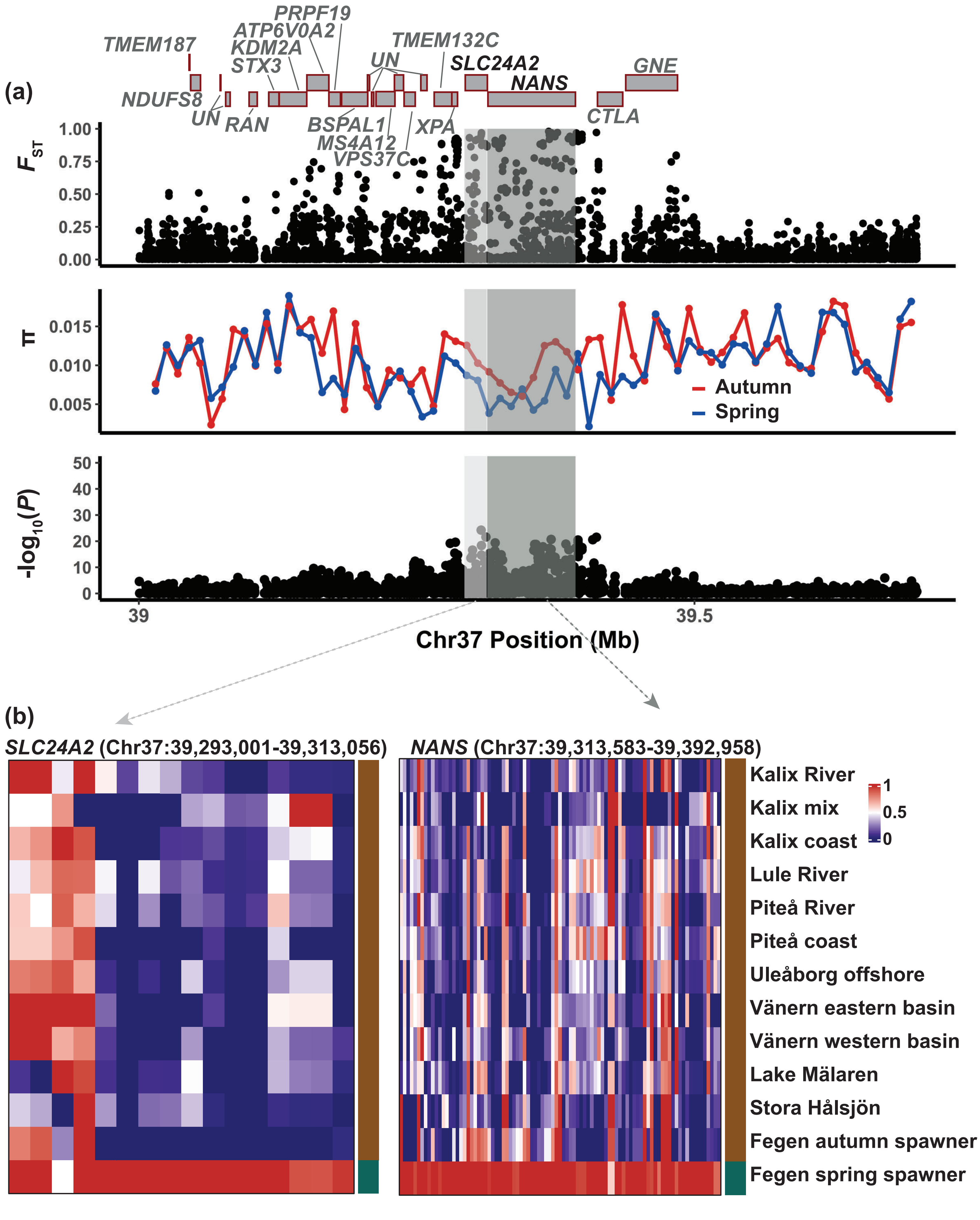

Figure S16. Examples of highly differentiated genes in the contrast spring spawners vs. autumn spawners in Lakes Fegen and Stora Hålsjön in European cisco - *SLC24A2 and NANS*. (a) Genome-wide diversity statistics F_{ST}, π and $-\log_{10}(P)$ across the *SLC24A2* and *NANS* locus on Chr37. F_{ST} and $-\log_{10}(P)$ represent single SNP data while π is calculated for 10-kb windows. The boxes with red borders indicate the genes surrounding the signals. The highlighted gene is the one closest to the most significant SNPs among the genes. '*UN*' denotes an unannotated gene. (b) Heatmaps of allele frequencies across the *SLC24A2* and *NANS*.

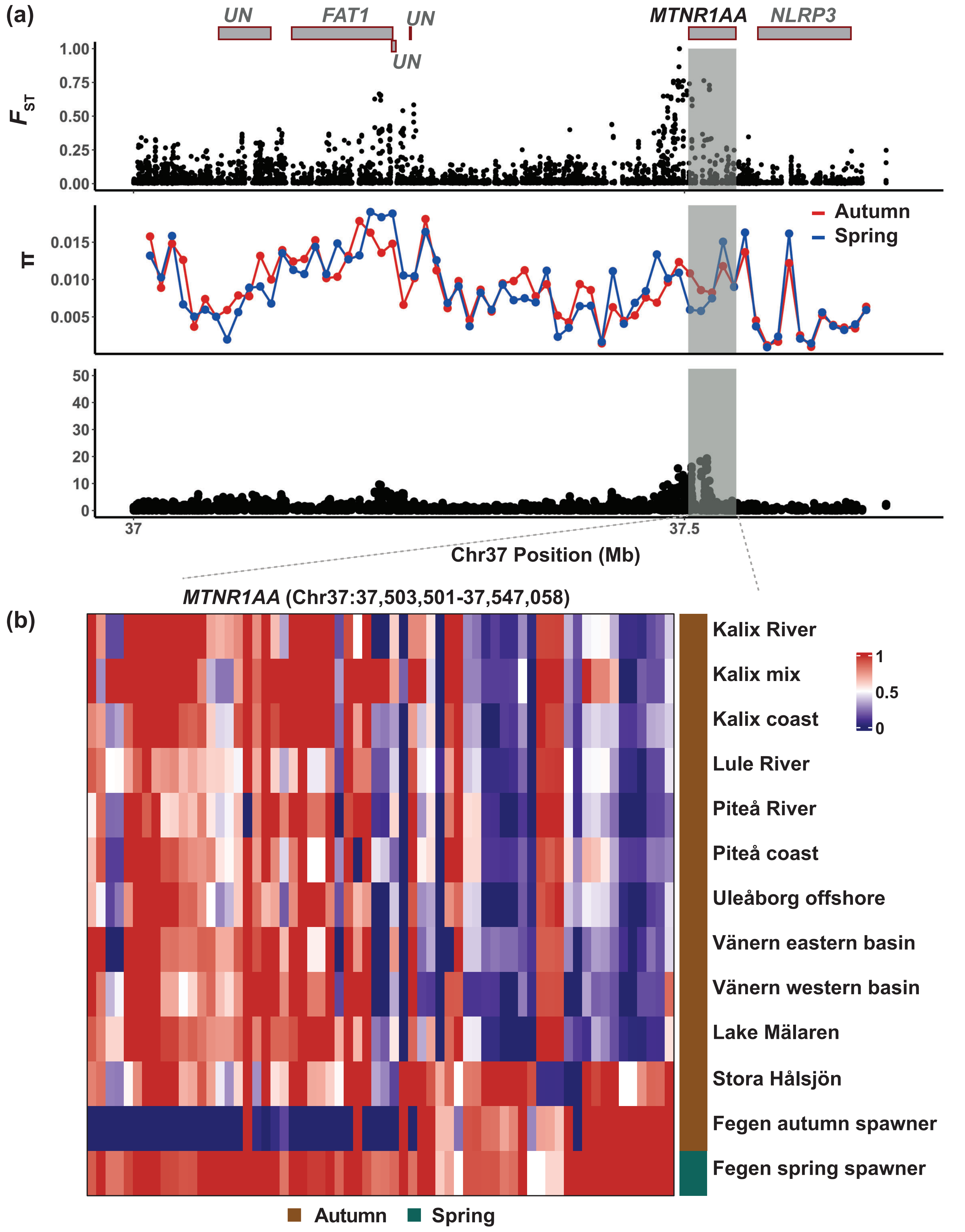

Figure S17. Examples of highly differentiated genes in the contrast spring spawners vs. autumn spawners in Lakes Fegen and Stora Hålsjön in European cisco - *MTNR1AA*. (a) Genome-wide diversity statistics F_{ST}, π and -log$_{10}$(*P*) across the *MTNR1AA* locus on Chr37. F_{ST} and -log$_{10}$(*P*) represent single SNP data while π is calculated for 10-kb windows. The boxes with red borders indicate the genes surrounding the signals. The highlighted gene is the one closest to the most significant SNPs among the genes. '*UN*' denotes an unannotated gene. (b) Heatmap of allele frequencies across the *MTNR1AA* region.

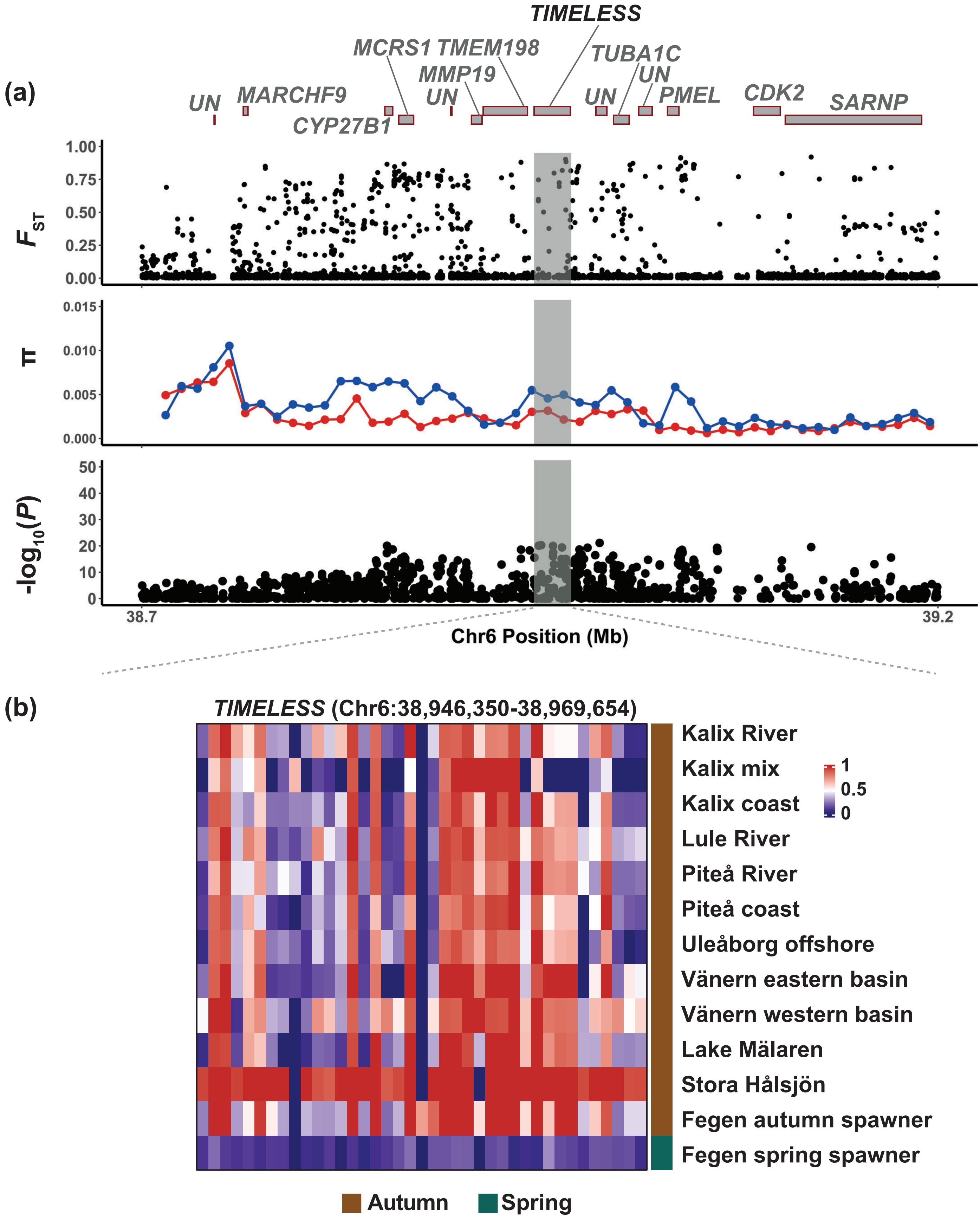

Figure S18. Examples of highly differentiated genes in the contrast spring spawners vs. autumn spawners in Lakes Fegen and Stora Hålsjön in European cisco - *TIMELESS*. (a) Genome-wide diversity statistics F_{ST}, π and -$\log_{10}(P)$ across the *TIMELESS* locus on Chr6. F_{ST} and -$\log_{10}(P)$ represent single SNP data while π is calculated for 10-kb windows. The boxes with red borders indicate the genes surrounding the signals. The highlighted gene is the one closest to the most significant SNPs among the genes. '*UN*' denotes an unannotated gene. (b) Heatmap of allele frequencies across the *TIMELESS* region.

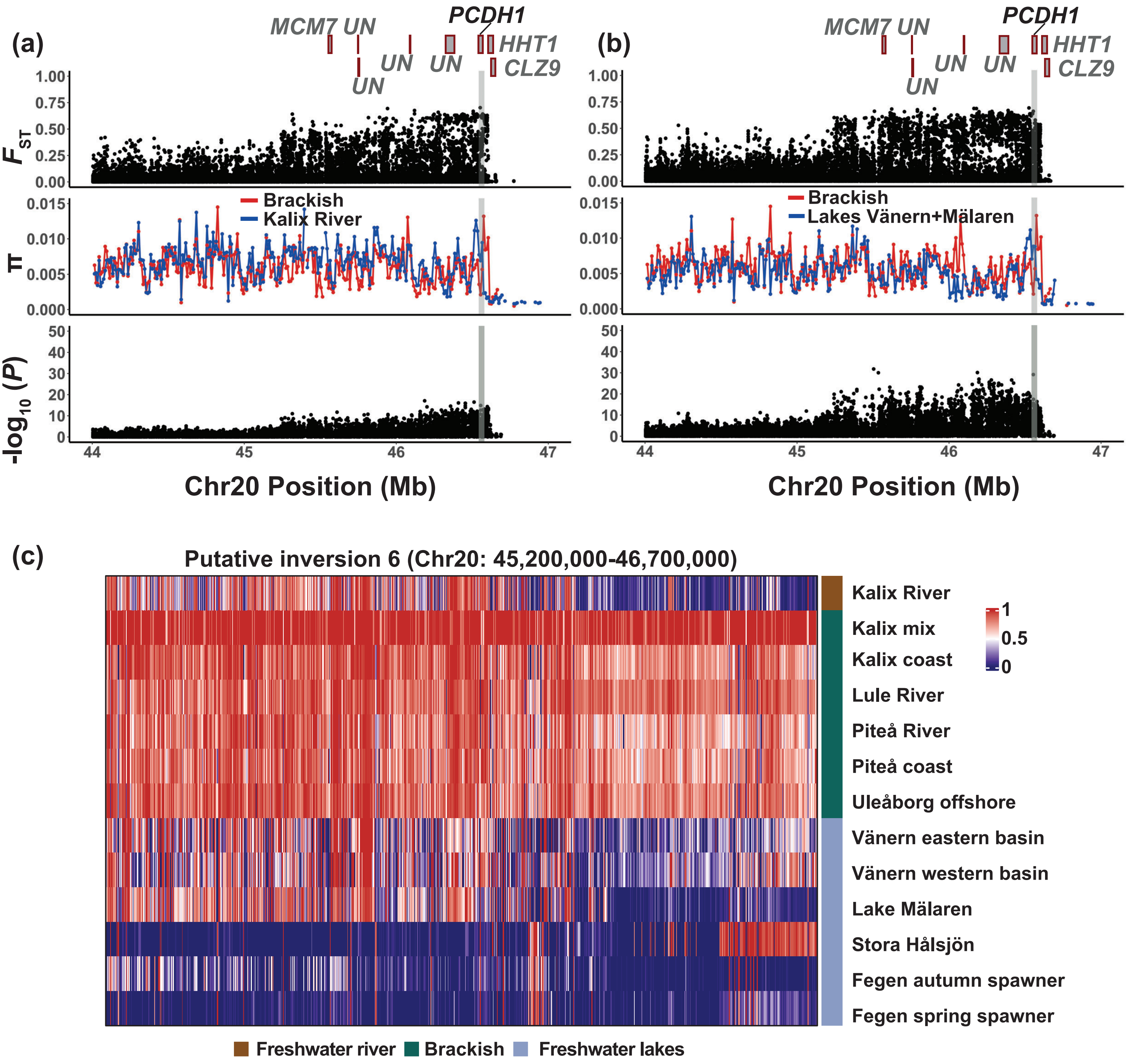

Figure S19. Example of shared signal of genetic differentiation in the vicinity of the putative inversion 6 in contrast_c: freshwater Kalix River vs. all other population samples from the Bothnian Bay area (riverine and coastal) and contrast_d: freshwater lakes Vänern + Mälaren vs. population samples from the Bothnian Bay area (riverine and coastal) after excluding Kalix River in European cisco. (a,b) Genome-wide diversity statistics F_{ST}, π and $-\log_{10}(P)$ across the putative inversion 6 locus on Chr20. F_{ST} and $-\log_{10}(P)$ represent single SNP data while π is calculated for 10-kb windows. The boxes with red borders indicate the genes surrounding the signals.The highlighted gene *PCDH1* appears as a shared signal of genetic differentiation in both contrasts. '*UN*' denotes unannotated genes. (a) Freshwater Kalix River vs. all other population samples from the Bothnian Bay area (riverine and coastal). (b) Freshwater lakes Vänern + Mälaren vs. population samples from the Bothnian Bay area (riverine and coastal) after excluding Kalix River. (c) Heatmap of allele frequencies across the putative inversion 6 locus.

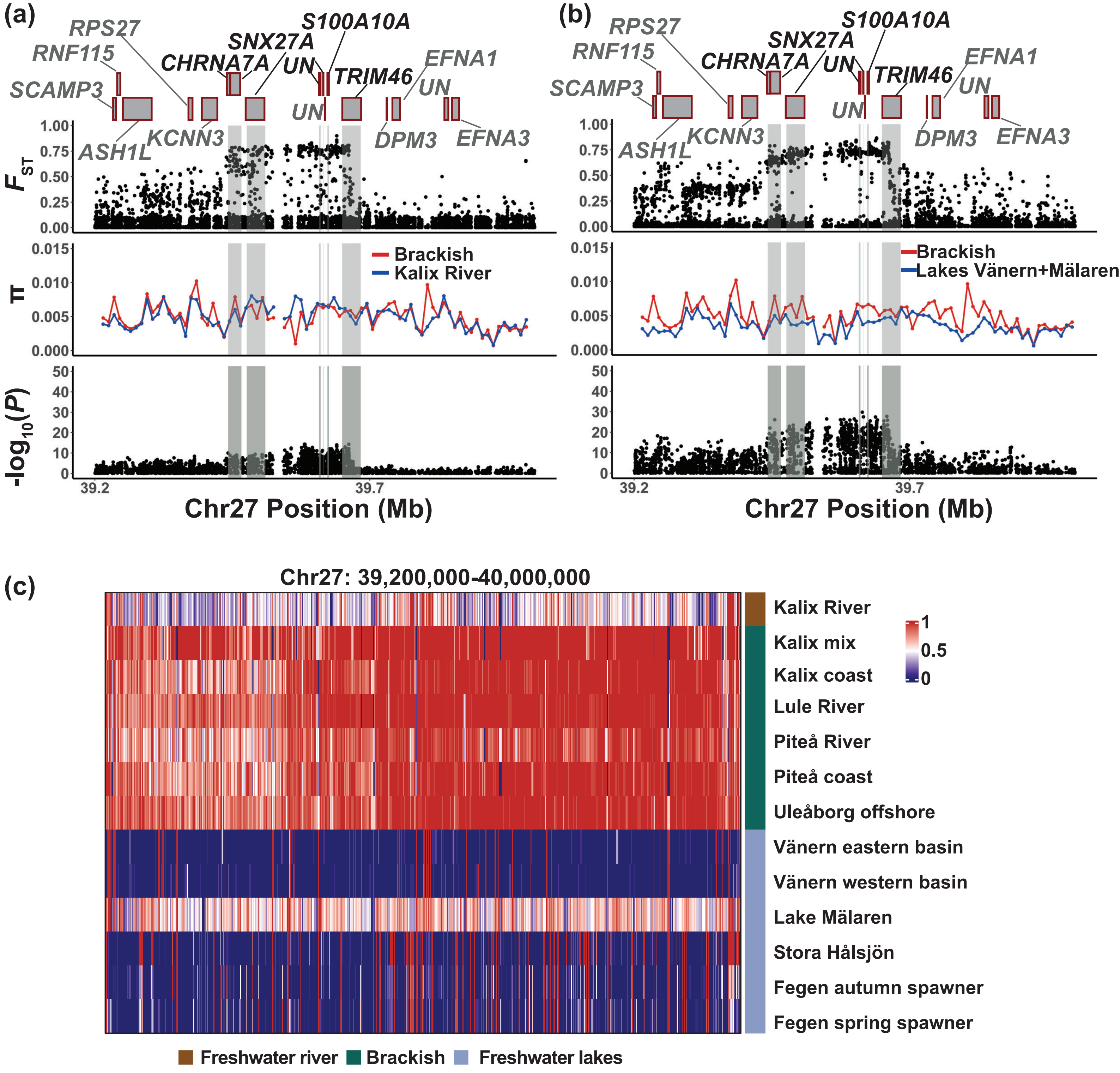

Figure S20. Example of shared signal of genetic differentiation in the vicinity of the *CHRNA7A, SNX27A, S100A10A,* and *TRIM4* loci in contrast_c: freshwater Kalix River vs. all other population samples from the Bothnian Bay area (riverine and coastal) and contrast_d: freshwater lakes Vänern + Mälaren vs. population samples from the Bothnian Bay area (riverine and coastal) after excluding Kalix River in European cisco. (a,b) Genome-wide diversity statistics F_{ST}, π and $-\log_{10}(P)$ across the *CHRNA7A, SNX27A, S100A10A,* and *TRIM46* loci on Chr27. F_{ST} and $-\log_{10}(P)$ represent single SNP data while π is calculated for 10-kb windows. The boxes with red borders indicate the genes surrounding the signals. '*UN*' denotes unannotated genes. The highlighted gene is the ones closest to the most significant SNPs. (a) Freshwater Kalix River vs. all other population samples from the Bothnian Bay area (riverine and coastal). (b) Freshwater lakes Vänern + Mälaren vs. population samples from the Bothnian Bay area (riverine and coastal) after excluding Kalix River. (c) Heatmap of allele frequencies across the shared signal of genetic differentiation on Chr27.

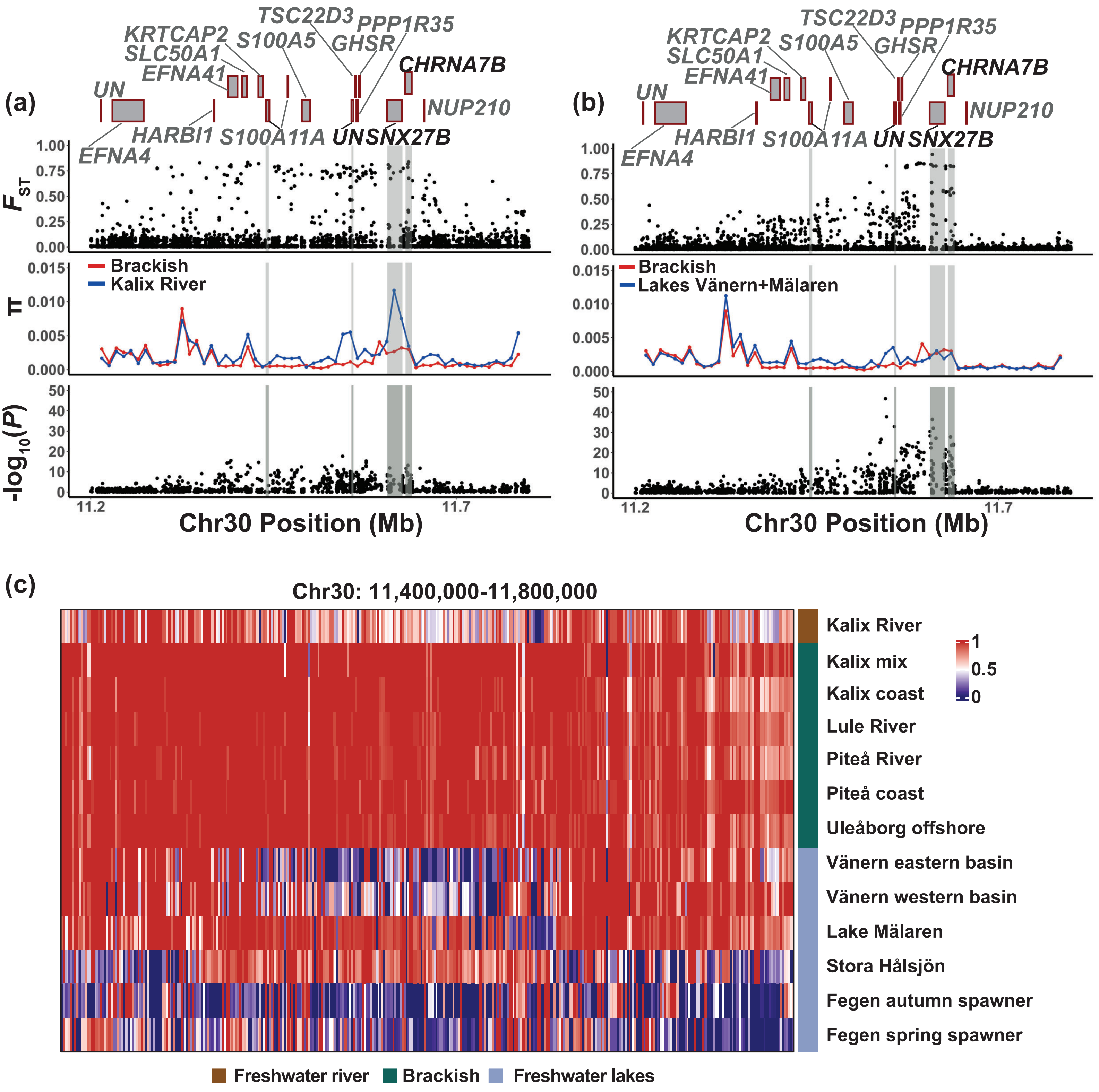

Figure S21. Example of shared signal of genetic differentiation in the vicinity of the *SNX27B* and *CHRNA7B* loci in contrast_c: freshwater Kalix River vs. all other population samples from the Bothnian Bay area (riverine and coastal) and contrast_d: freshwater lakes Vänern + Mälaren vs. population samples from the Bothnian Bay area (riverine and coastal) after excluding Kalix River in European cisco. (a,b) Genome-wide diversity statistics F_{ST}, π and $-\log_{10}(P)$ across the *SNX27B* and *CHRNA7B* loci on Chr30. F_{ST} and $-\log_{10}(P)$ represent single SNP data while π is calculated for 10-kb windows. The boxes with red borders indicate the genes surrounding the signals. '*UN*' denotes unannotated genes. The highlighted gene is the ones closest to the most significant SNPs. (a) Freshwater Kalix River vs. all other population samples from the Bothnian Bay area (riverine and coastal). (b) Freshwater lakes Vänern + Mälaren vs. population samples from the Bothnian Bay area (riverine and coastal) after excluding Kalix River. (c) Heatmap of allele frequencies across the shared signal of genetic differentiation on Chr30.

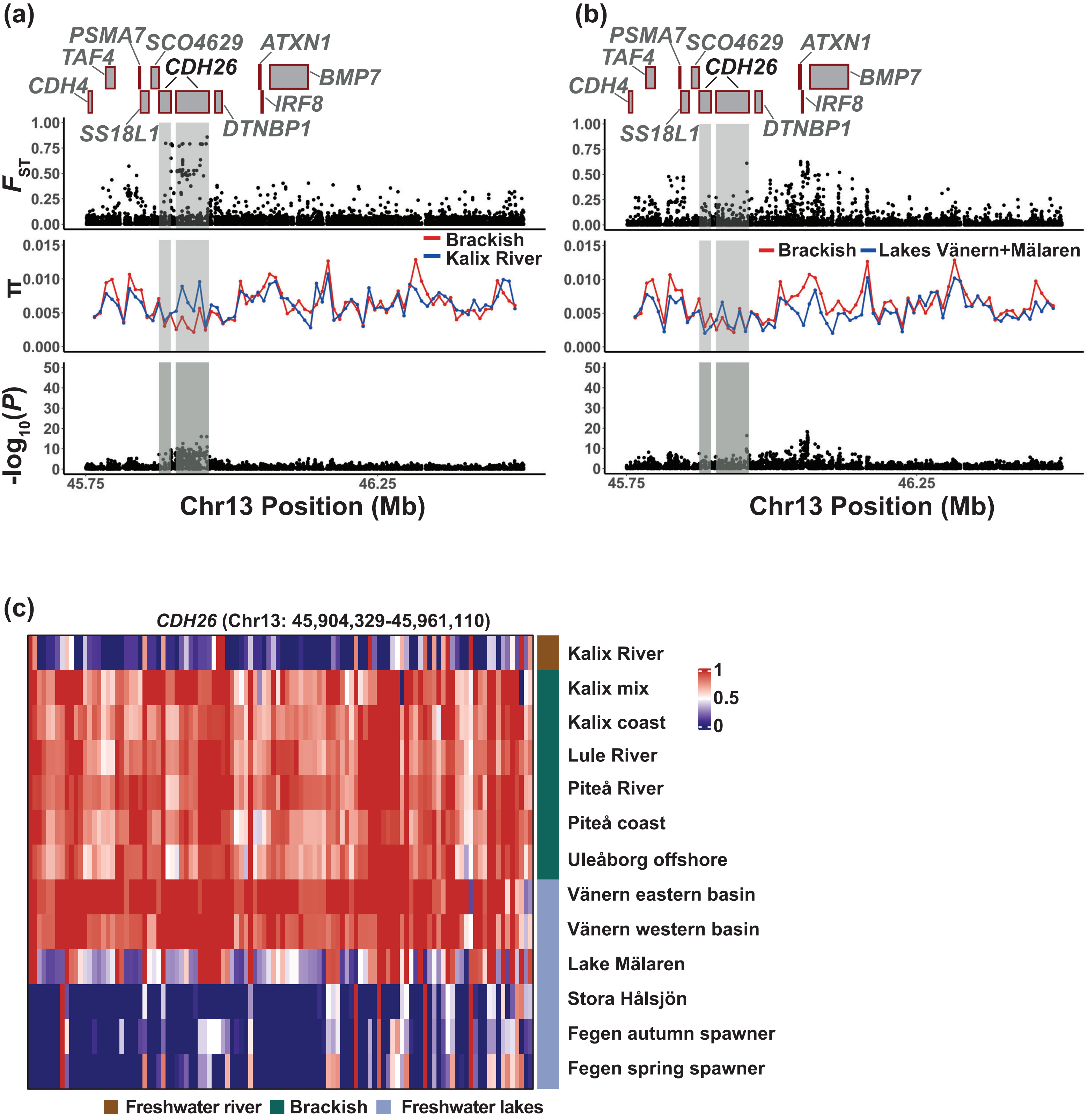

Figure S22. Examples of highly differentiated genes between contrast freshwater Kalix River vs. Bothnian Bay in European cisco - *CDH26*. (a) Genome-wide diversity statistics F_{ST}, π and -log$_{10}$(P) across the *CDH26* locus on Chr13. F_{ST} and -log$_{10}$(P) represent single SNP data while π is calculated for 10-kb windows. The boxes with red borders indicate the genes surrounding the signals. The highlighted gene is the one closest to the most significant SNPs among the genes. (b) Heatmap of allele frequencies across the *CDH26* region.

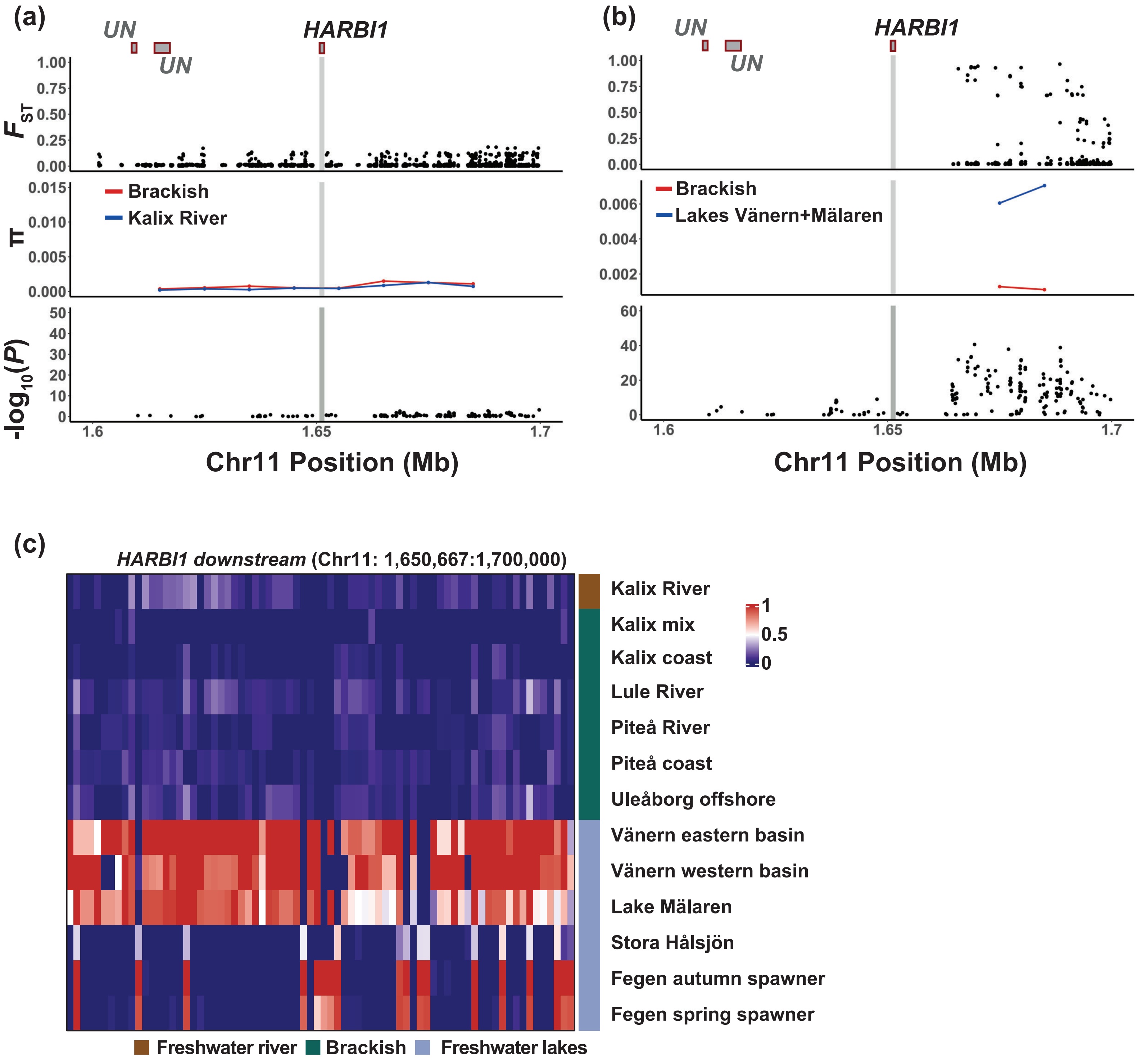

Figure S23. Examples of highly differentiated genes in the contrast_d: freshwater lakes Vänern + Mälaren vs. population samples from the Bothnian Bay area (riverine and coastal) after excluding Kalix River - downstream of *HARBI1*. (a, b) Genome-wide diversity statistics F_{ST}, π and $-\log_{10}(P)$ across the *HARBI1* downstream locus on Chr11. F_{ST} and $-\log_{10}(P)$ represent single SNP data while π is calculated for 10-kb windows. The boxes with red borders indicate the genes surrounding the signals. The highlighted gene is the one closest to the most significant SNPs among the genes. '*UN*' denotes an unannotated gene. (a) Freshwater Kalix River vs. all other population samples from the Bothnian Bay area (riverine and coastal). (b) Freshwater lakes Vänern + Mälaren vs. population samples from the Bothnian Bay area (riverine and coastal) after excluding Kalix River.(c) Heatmap of allele frequencies across the *HARBI1* downstream locus.

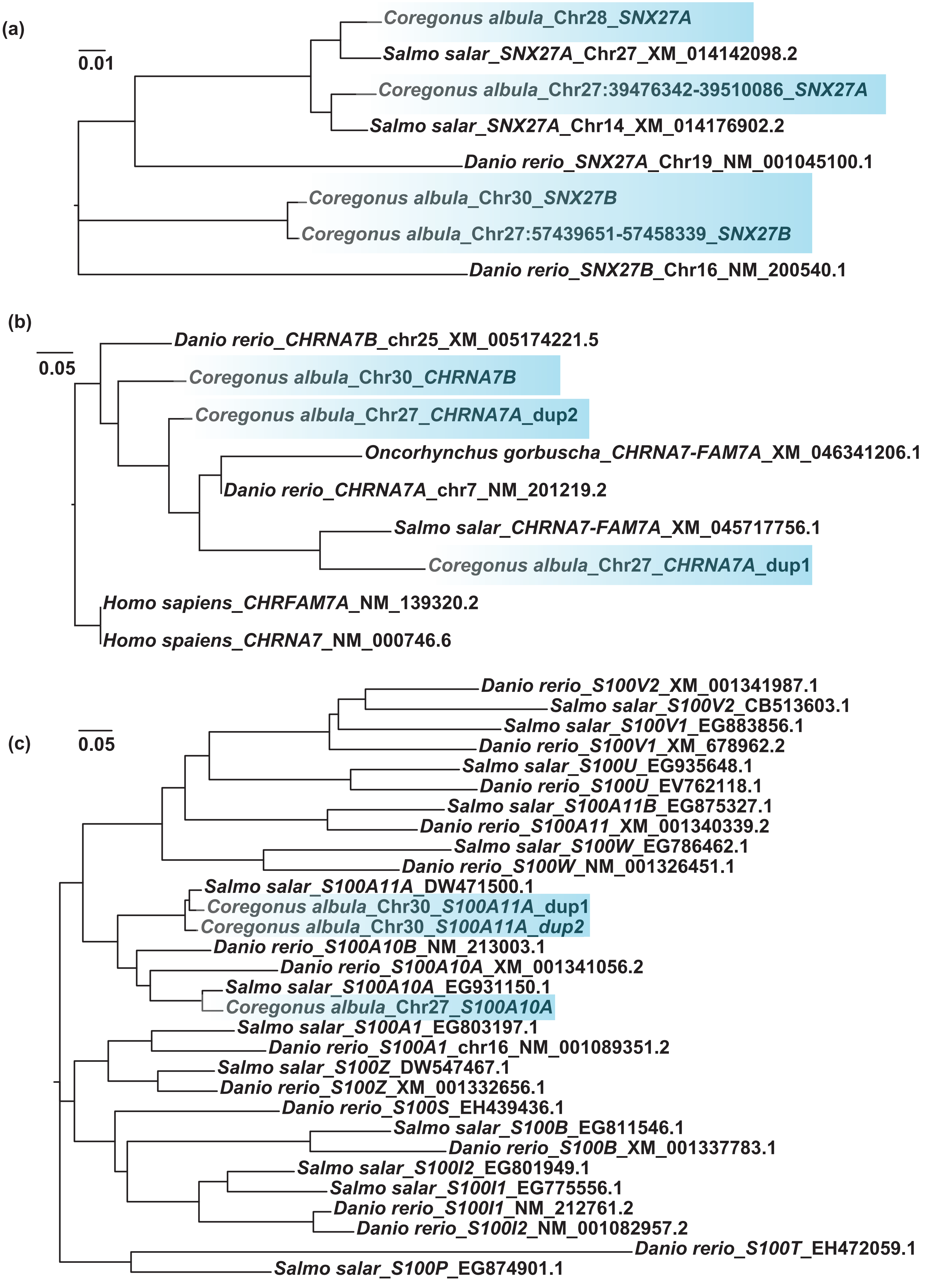

Figure S24. Phylogenetic tree of *SNX27*, *CHRNA7*, and *S100A* nucleotide sequences. The tree was constructed using the Neighbor-Joining (NJ) method. Sequences from European cisco are highlighted in blue boxes.

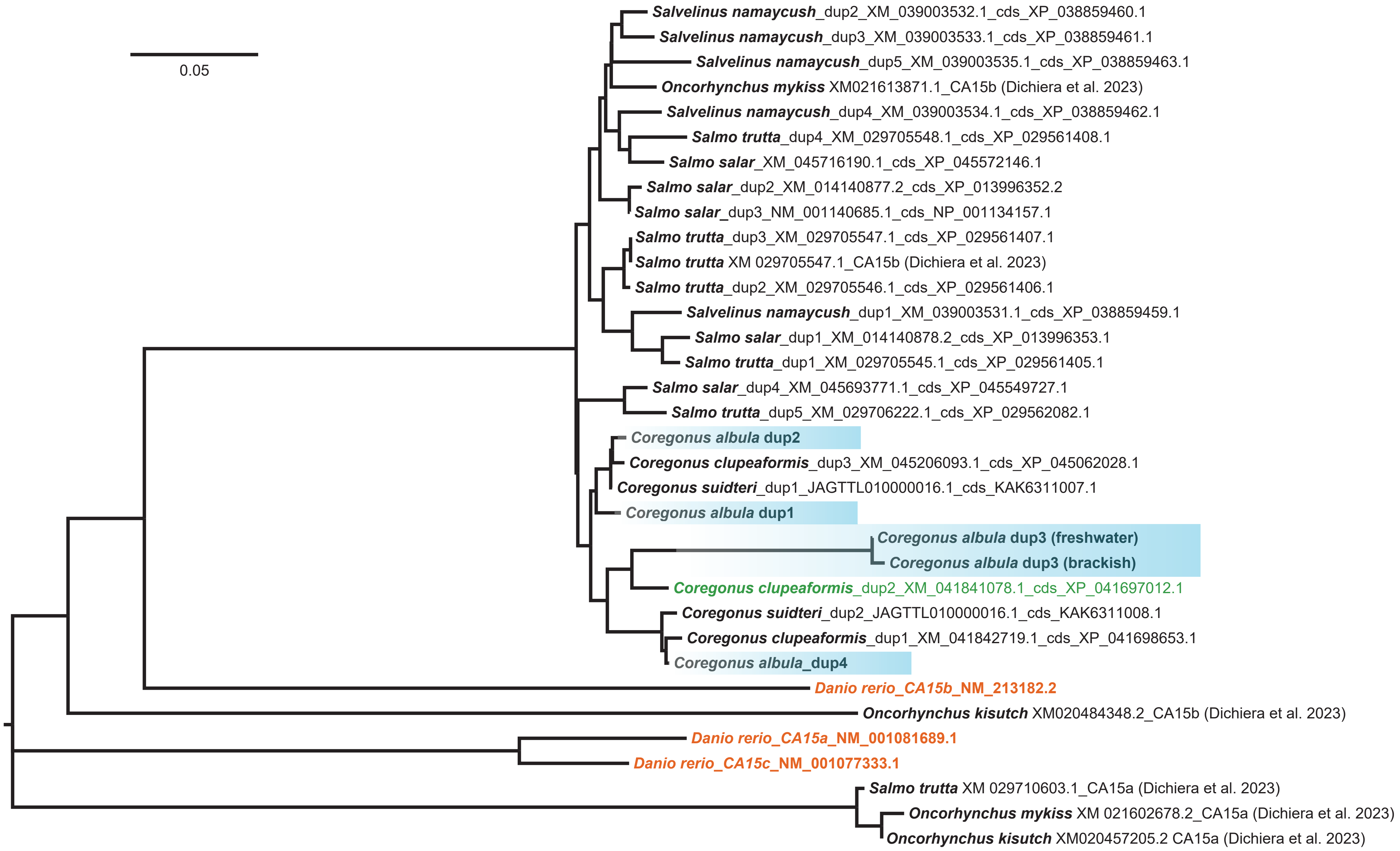

Figure S25. Phylogenetic tree based on *CA15* nucleotide sequences in salmonids. The tree was constructed using the Neighbor-Joining (NJ) method. The tree includes the *CA15a*, *CA15b*, and *CA15c* sequences from zebrafish, highlighted in orange. *CA15b* copies from European cisco are highlighted in blue boxes. *CA4* in Lopez et al. (2022) was blasted against the reference genome of *Coregonus clupeaformis* and is highlighted in green in our tree, where it also clusters with zebrafish *CA15b*.
