# Supplementary material for: Genetic Adaptation to Brackish Water and Spawning Season in European Cisco
Source: Mol Ecol. 2025 Sep 3;34(20):e70094. doi: 10.1111/mec.70094 (PMC12530279; doi:10.1111/mec.70094)
Supplement: Supplementary file 3 — Table S1: Population samples of European cisco included in the study and their average genome‐wide nucleotide diversity (π). Table S2: Summary of gene annotations for the Coregonus albula reference genome, fCorAlb1. Table S3: Locus‐specific nucleotide diversity (π) of overlapping signals of genetic differentiation between contrast (c): freshwater Kalix River vs. all other population samples from the Bothnian Bay area (riverine and coastal) and (d): freshwater lakes Vänern + Mälaren vs. population samples from the Bothnian Bay area (riverine and coastal) after excluding Kalix River. [file MEC-34-e70094-s002.pdf]

Table S1. Population samples of European cisco included in the study and their average genome-wide nucleotide diversity (π).

Population	Abbreviation	Mean depth (genome-wide)	Mean depth (filter-passed)	Depth range (filter-passed)	Water body	Spawning time	n	π
Fegen autumn-spawner	FegA	1.08	2.50	2.15~3.24	Freshwater lake	Autumn	30	0.0030
Fegen spring-spawner	FegS	1.12	2.51	2.07~3.18	Freshwater lake	Spring	30	0.0031
Stora Hålsjön	Sto	0.85	2.23	1.70~3.02	Freshwater lake	Autumn	30	0.0028
Lake Mälaren	Mal	1.41	2.98	1.26~4.76	Freshwater lake	Autumn	30	0.0042
Vänern eastern basin	VanO	0.75	2.11	1.50~3.13	Freshwater lake	Autumn	18	0.0039
Vänern western basin	VanV	0.59	1.85	1.33~2.35	Freshwater lake	Autumn	18	0.0038
Piteå coast	PitK	1.08	2.58	1.59~3.61	Brackish	Autumn	30	0.0049
Piteå River	PitA	1.14	2.63	1.87~3.38	Brackish	Autumn	30	0.0048
Lule River	LulA	1.40	2.92	1.76~3.99	Brackish	Autumn	30	0.0049
Kalix coast	KalK	1.01	2.42	1.72~3.35	Brackish	Autumn	30	0.0047
Kalix mix	KalM	1.18	2.33	1.87~2.77	Brackish	Autumn	9	0.0043
Kalix River	KalA	1.14	2.61	2.12~3.82	Freshwater river	Autumn	21	0.0047
Uleåborg offshore	UleO	1.03	2.45	1.71~3.29	Brackish	Autumn	30	0.0048

[†]Note: n=number of individuals.

Table S2 Summary of gene annotations for the *Coregonus albula* reference genome, fCorAlb1.

Annotation category	Total no	With functional annotation	With gene name
Genes annotated	51,040	50,487 (99%)	42,326 (83%)
mRNAs annotated	125,922	123,114 (98%)	109,959 (87%)

Table S3 Locus-specific nucleotide diversity (π) of overlapping signals of genetic differentiation between contrast (c): freshwater Kalix River vs. all other population samples from the Bothnian Bay area (riverine and coastal) and (d): freshwater lakes Vänern + Mälaren vs. population samples from the Bothnian Bay area (riverine and coastal) after excluding Kalix River.

Signal	Position	π			Associated gene	Gene position
		Brackish	Kalix River	Vänern + Mälaren		
1	Chr13:11100242-11143518	0.0013	0.0018	0.0017	*TNFRSF14*	Chr13:11,046,090-11,145,369
2	Chr17:40098943-40399211	0.0063	0.0092	0.0056	*CA15b*	Chr17:39,868,455-40,459,526
3	Chr20:45267422-45290260	0.0053	0.0080	0.0054	*MCM7*	Chr20:45,265,774-45,292,853
4	Chr20:46548530-46579066	0.0049	0.0058	0.0070	*PCDH1*	Chr20:46,542,541-46,579,775
5	Chr27:39445275-39664796	0.0019	0.0059	0.0036	*CHRNA7A*<br>*CHRNA7A*<br>*SNX27A*<br>*S100A1A*<br>*TRIM46*	Chr27:39,442,552-39,448,787<br>Chr27:39,448,727-39,466,594<br>Chr27:39,476,342-39,510,086<br>Chr27:39,623,070-39,625,650<br>Chr27:39,649,852-39,683,692
6	Chr27:58321079-58447769	0.0011	0.0010	0.0009	*MYH10*<br>*NPTX2*<br>*BAIAP2*	Chr27:58,316,895-58,361,253<br>Chr27:58,409,519-58,432,228<br>Chr27:58,442,308-58,458,846
7	Chr28:71305147-71316314	0.0134	0.0123	0.0104	*OLPB*<br>*CLDND1*	Chr28:71,292,149-71,305,887<br>Chr28:71,316,231-71,320,420
8	Chr30:11566381-11570548	0.0004	0.0006	0.0016	*GHSR*	Chr30:11,566,919-11,568,453
9	Chr30:11605811-11638133	0.0016	0.0047	0.0026	*SNX27B*<br>*CHRNA7B*	Chr30:11,605,784-11,626,573<br>Chr30:11,630,614-11,639,685
Genome-wide		0.0049	0.0046	0.0044		

Locus-specific nucleotide diversity (π) represents the average π value within the peak signal regions (Fst > 0.5). The last row shows genome-wide nucleotide diversity for comparison.
